# p53-mediated control of aspartate-asparagine homeostasis dictates LKB1 activity and modulates cell survival

**DOI:** 10.1038/s41467-020-15573-6

**Published:** 2020-04-09

**Authors:** Longfei Deng, Pengbo Yao, Le Li, Fansen Ji, Shuang Zhao, Chang Xu, Xun Lan, Peng Jiang

**Affiliations:** 1 Tsinghua-Peking Joint Center for Life Sciences, Tsinghua University, 100084 Beijing, China; 2 School of Life Sciences, Tsinghua University, 100084 Beijing, China; 3 School of Medicine, Tsinghua University, 100084 Beijing, China

**Keywords:** Cancer metabolism, Cell signalling

## Abstract

Asparagine synthetase (ASNS) catalyses the ATP-dependent conversion of aspartate to asparagine. However, both the regulation and biological functions of asparagine in tumour cells remain largely unknown. Here, we report that p53 suppresses asparagine synthesis through the transcriptional downregulation of ASNS expression and disrupts asparagine-aspartate homeostasis, leading to lymphoma and colon tumour growth inhibition in vivo and in vitro. Moreover, the removal of asparagine from culture medium or the inhibition of ASNS impairs cell proliferation and induces p53/p21-dependent senescence and cell cycle arrest. Mechanistically, asparagine and aspartate regulate AMPK-mediated p53 activation by physically binding to LKB1 and oppositely modulating LKB1 activity. Thus, we found that p53 regulates asparagine metabolism and dictates cell survival by generating an auto-amplification loop via asparagine-aspartate-mediated LKB1-AMPK signalling. Our findings highlight a role for LKB1 in sensing asparagine and aspartate and connect asparagine metabolism to the cellular signalling transduction network that modulates cell survival.

## Abstract (web summary)

Cancer cells can produce asparagine, yet both the regulation and biological functions of asparagine are unclear. Here the authors show that p53 suppresses asparagine synthesis and disrupts asparagine-aspartate homeostasis contributing to tumor growth inhibition, and that asparagine and aspartate modulates AMPK-mediated p53 activation by physically binding to and regulating LKB1.

## Introduction

The dysregulation of asparagine synthetase (ASNS) expression in childhood acute lymphoblastic leukaemia (ALL) cells is considered to increase cell susceptibility to the toxicity of l-asparaginase (ASNase), a first-line therapy for ALL that breaks down asparagine^1–3^. ASNS inhibition also renders some types of tumour cells more susceptible to glutamine withdrawal-induced apoptosis, and asparagine addition sufficiently reverses this effect independent of TCA cycle anaplerosis^4^. ASNS and asparagine may be crucial for tumour cell proliferation, as the depletion of either can arrest cell proliferation and/or induce apoptosis in some types of tumour cells^2,4,5^. However, the mechanisms underlying these observations are poorly understood.

The tumour suppressor p53 is the most frequently mutated gene in human cancers^6^. Consistent with this, p53-knockout mice are highly prone to the spontaneous development of different tumours^7,8^. However, ~70% of spontaneous tumours arising in p53-deficient mice are lymphomas^9,10^, with the underlying mechanisms unknown. The activation of p53 is able to induce a range of antiproliferative responses, including cell apoptosis, senescence and differentiation, and metabolic regulation appears to be central to the tumour-suppressive function of p53^11,12^. However, in addition to the induction of permanent proliferation arrest or cell death, under some mild metabolic stresses, such as transient nutrient starvation, p53 activation confers adaptation to stress and helps cells survive^12–15^. This survival-supporting ability of p53 is mostly implemented by a successful p21-mediated pause in cell cycle progression, which allows cells to repair DNA lesions and/or maintain metabolic homeostasis^12,16^.

Here, we report that p53 plays a role in regulating asparagine metabolism by repressing the expression of ASNS. High levels of ASNS or asparagine maintain cell survival and promote tumour cell proliferation via stifling AMPK-mediated p53 activation. Also, to generalise how asparagine may affect tumours in a broader context, we extended our study into various human cell lines besides mouse lymphoma. Moreover, by studying these factors, we found that LKB1 is a natural sensor of cellular asparagine-aspartate homeostasis, uncovering a role for asparagine as a signalling molecule in tumour growth.

## Results

### p53 deficiency supports cell proliferation though Asn

**Fig. 1:**
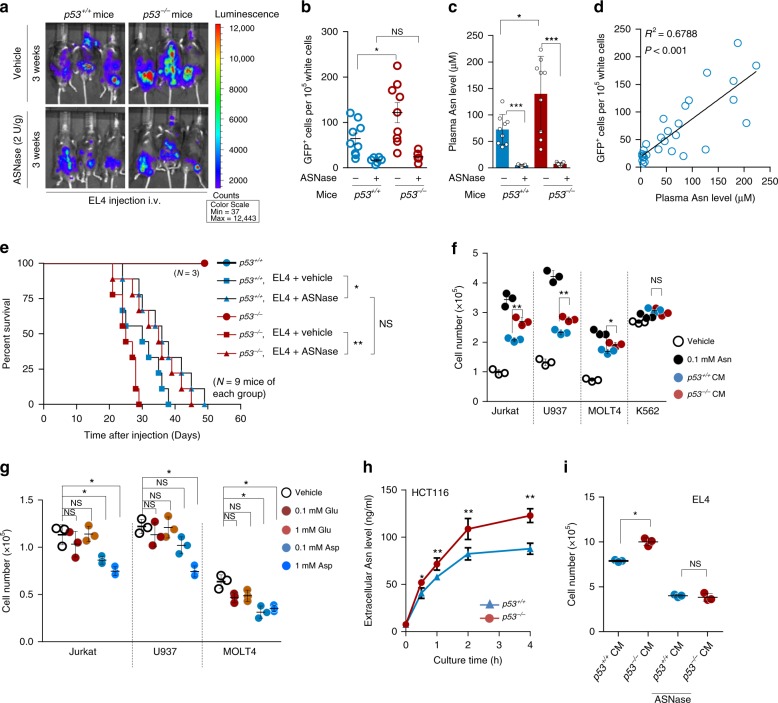
p53 deficiency increases asparagine secretion to promote lymphoma proliferation. **a** *p53*^+/+^ and *p53*^−/−^ C57BL/6J mice injected i.v. with 5 × 10^5^ EL4-Luc-GFP cells were treated i.p. with vehicle or ASNase (2 U/g) every 3 days for 3 weeks. Whole-mouse bioluminescence analysis and representative images are shown. The colour scale represents the intensity of emitted luminescence. Each group included 9 mice. i.v., intravenously; i.p., intraperitoneally. **b** Flow cytometry analysis of the frequencies of EL4-Luc-GFP cells (GFP^+^) in peripheral blood in recipient mice as treated in **a**. Peripheral blood from mice without injection/treatment was used as a control. **c** *p53*^+/+^ and *p53*^−/−^ C57BL/6J mice were treated as in **a**, plasma Asn levels were determined by LC-MS. **d** Linear regression analysis of the concentrations of mouse plasma Asn and the number of EL4-Luc-GFP (GFP^+^) cells in peripheral blood. *n* = 36 mice. **e** Kaplan–Meier survival curves of *p53*^+/+^ and *p53*^−/−^ C57BL/6J mice treated as in **a**. Mice without EL4 injection/treatment were used as controls (*n* = 3). The significant differences among groups were determined by the log-rank (Mantel-Cox) test. **f** Proliferation of indicated cell lines cultured for 48 h in DMEM containing no or 0.1 mM Asn or cultured in *p53*^*+/+*^ or *p53*^*−/−*^ HCT116 cell conditioned-medium (CM) as indicated. **g** Proliferation of cells cultured in DMEM containing 0, 0.1 or 1 mM glutamate (Glu) or aspartate (Asp) for 48 h. **h** Asparagine levels in culture medium from *p53*^*+/+*^ or *p53*^*−/−*^ HCT116 cells at the indicated culture time points were measured. **i** The cultured medium from *p53*^*+/+*^ or *p53*^*−/−*^ HCT116 cells cultured for 24 h was used for culturing EL4 cells with 0 or 2 IU/ml ASNase for another 48 h. Cell proliferation was measured. Data are mean ± s.d., unpaired two-tailed Student’s *t*-test, *p < 0.05, **p < 0.01, ***p < 0.001, NS not significant. Source data are provided as a Source Data file.

p53-null mice predominantly develop and succumb to lymphomas^9,10,17^. We thus hypothesised that there might be some advantage(s) provided by p53 deficiency that can facilitate tumourigenesis in plasma. To test this hypothesis, we transplanted luciferase- and GFP-labelled p53-wildtype murine lymphoma EL4 cells into mice via the tail vein, and tumour cell proliferation was then monitored by whole-body imaging for luciferase activity and flow cytometry analysis for GFP expression. Strikingly, higher EL4 cell proliferation was observed in *p53*^*−/−*^ mice than in *p53*^*+/+*^ mice (Fig. 1a (top panels), b; and Supplementary Figs. 1a and 11a), suggesting that *p53*^*−/−*^ mouse plasma may provide signals that promote EL4 cell proliferation.

To determine what those signals are, we found that the levels of asparagine (Asn), not glutamate (Glu), were significantly higher in *p53*^*−/−*^ mouse serum than in *p53*^*+/+*^ mouse serum (Fig. 1c and Supplementary Fig. 1b, c). To explore whether asparagine mediates the enhancement of lymphoma cell proliferation, we intraperitoneally injected mice with ASNase to remove plasma asparagine, which consequentially led to the accumulation of aspartate (Fig. 1c and Supplementary Fig. 1d). Notably, the removal of plasma asparagine suppressed EL4 cell proliferation in vivo (Fig. 1a (bottom panels), b; and Supplementary Fig. 1a). Consistently, plasma asparagine levels positively correlated with the EL4 cell proliferative rate (Fig. 1d). Moreover, the transplantation of EL4 cells substantially reduced the lifespan of mice, particularly *p53*^*−/−*^ mice (Fig. 1e). Treatment with ASNase reversed this effect and minimised the difference between *p53*^*+/+*^ and *p53*^*−/−*^ mice (Fig. 1e). The effect of ASNase on lymphoma suppression may not be due to its toxicity, as no changes in body weight were observed (Supplementary Fig. 1e). These findings were further confirmed by subcutaneous cell transplantation assays. *p53*^*−/−*^ mice had obviously larger tumours than did *p53*^*+/+*^ mice, and ASNase supplementation reduced tumour growth and abolished the difference in both tumour sizes and plasma asparagine levels between *p53*^*+/+*^ and *p53*^*−/−*^ mice (Supplementary Fig. 1f–h). Furthermore, a positive correlation between plasma asparagine levels and tumour sizes was found (Supplementary Fig. 1i), in agreement with the intravenous injection data (Fig. 1d). Together, increased lymphoma cell proliferation in *p53*^*−/−*^ mice may be due to elevated plasma asparagine.

Next, we extended these findings by culturing lymphoma cells in vitro. Notably, asparagine addition promoted the proliferation of multiple types of lymphoma cells (Jurkat, U937 and MOLT4 cells) (Fig. 1f). Likewise, tumour-conditioned medium enhanced the proliferation of these cells, with *p53*^*−/−*^ cell-conditioned medium having a more profound effect (Fig. 1f). Consistently, asparagine or tumour-conditioned medium maintained cell survival, and *p53*^*−/−*^ tumour-conditioned medium had a stronger effect (Supplementary Fig. 2a). To assess the generalisability of these findings, we used tumour-conditioned medium from U2OS cells expressing p53 shRNA or control shRNA to culture lymphoma cells and similar results were obtained (Supplementary Fig. 2b). In accordance with these findings, cells treated with asparagine at levels that were found in *p53*^*−/−*^ mouse plasma (0.13 mM) proliferated faster and survived better than those cultured in medium containing asparagine at 0.075 mM, as found in *p53*^*+/+*^ mouse plasma (Fig. 1c and Supplementary Fig. 2c, d). Moreover, physiological levels of asparagine sufficiently enhanced lymphoma growth in soft agar (Supplementary Fig. 2e, f). Asparagine is derived from glutamine or aspartate. In contrast to asparagine, aspartate or glutamate did not promote cell proliferation, whereas aspartate visibly suppressed it (Fig. 1g and Supplementary Fig. 2g). We noticed that K562 cells, which are used as control cells, exhibited resistance to treatment with either asparagine or tumour-conditioned medium (Fig. 1f and Supplementary Fig. 2c), which may be because these cells can de novo produce sufficient asparagine by expressing high levels of ASNS (Supplementary Fig. 2h). Collectively, these data suggest that tumour cells, particularly p53-depleted cells, can fuel the proliferation of surrounding cells by secreting asparagine. To further confirm this, we directly measured asparagine levels in cultured medium. Indeed, asparagine levels elevated rapidly, and p53-depleted cell medium had higher levels of asparagine than did medium from p53-wildtype cells (Fig. 1h and Supplementary Fig. 2i). Consistent with the findings in vivo (Fig. 1a–d), EL4 cells cultured with *p53*^*−/−*^ tumour-conditioned medium proliferated faster than those cultured with *p53*^*+/+*^ tumour-conditioned medium. Conversely, ASNase treatment reduced cell proliferation and minimised the differences between cells cultured with *p53*^*−/−*^ and *p53*^*+/+*^ tumour-conditioned medium (Fig. 1i and Supplementary Fig. 2j). Coculture experiments also revealed that *p53*^*−/−*^ cells markedly increased the proliferation of EL4 cells (Supplementary Fig. 2k), and the further addition of ASNase resulted in cell proliferation inhibition (Supplementary Fig. 2l). Taken together, these findings indicate that tumour cells produce asparagine to promote cell proliferation, which is enhanced by p53 loss.

### Identification of ASNS as a target of p53

**Fig. 2:**
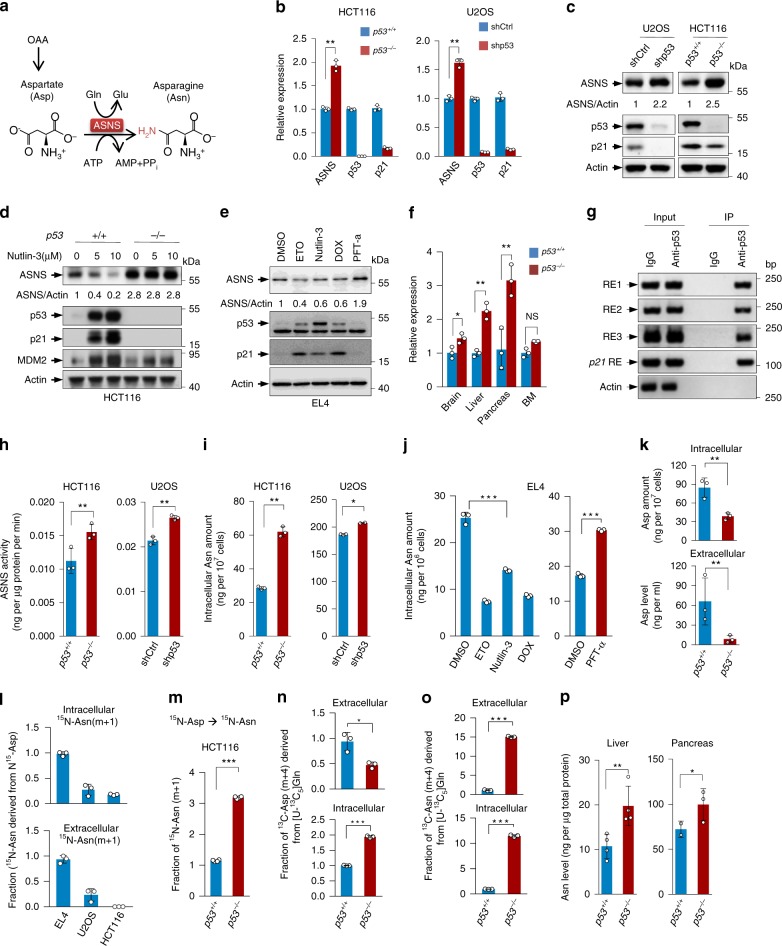
p53 regulates ASNS transcript and asparagine metabolism. **a** Schematic diagram describing the reaction catalysed by ASNS. **b** Quantitative RT-PCR analysis of ASNS expression in *p53*^*+/+*^ and *p53*^*−/−*^ HCT116 cells or in shCtrl and shp53 U2OS cells. **c** Protein expression in *p53*^*+/+*^ and *p53*^*−/−*^ HCT116 cells and in U2OS cells stably expressing control or p53 shRNA. Relative ASNS/Actin ratios are shown**. d** ASNS protein expression in *p53*^*+/+*^ and *p53*^*−/−*^ HCT116 cells treated with 0, 5 or 10 μM Nutlin-3 for 36 hours. **e** Protein expression in EL4 cells treated with the indicated chemical compounds for 24 h. **f** ASNS mRNA levels in the brain, liver, pancreas and bone marrow (BM) from *p53*^+/+^ and *p53*^−/−^ mice. **g** Binding of p53 to the *ASNS* genomic locus in HCT116 cells was analysed by chromatin immunoprecipitation assay using anti-p53 antibody. Isotype mouse IgG was used as a control. Three potential p53 response elements (REs) were individually amplified by PCR. **h**, **i** ASNS activity (**h**) and intracellular amount of Asn (**i**) in *p53*^*+/+*^ and *p53*^*−/−*^ HCT116 cells and in shCtrl and shp53 U2OS cells. **j** Asn levels in EL4 cells treated as in **e**. **k** Asp levels within and outside *p53*^*+/+*^ and *p53*^*−/−*^ HCT116 cells. **l** Intracellular and extracellular labelling fraction of Asn after 24 h of incubation with ^15^N-aspartate. **m** Labelling fraction of Asn after 48 h of incubation with ^15^N-aspartate in *p53*^*+/+*^ and *p53*^*−/−*^ HCT116 cells. **n**, **o** Intracellular and extracellular labelling fraction of Asp or Asn after 24 h of incubation with [U-^13^C_5_]glutamine in *p53*^*+/+*^ and *p53*^*−/−*^ HCT116 cells. **p** Levels of Asn in liver and pancreas tissues from *p53*^+/+^ and *p53*^−/−^ mice. Data are mean ± s.d., unpaired two-tailed Student’s *t*-test, *p < 0.05, **p < 0.01, ***p < 0.001, NS not significant. Source data are provided as a Source Data file.

We next investigated how p53 affects asparagine production. ASNS is critical for maintaining the physiological equilibrium between asparagine and aspartate (Fig. 2a). To study whether ASNS is a physiological target for p53, and also due to the limited transfection efficiency of lymphomas, we examined the effect of p53 on ASNS expression in lymphomas and various human tumour cell lines through different approaches. By comparing the gene expression of *p53*^*+/+*^ and *p53*^*−/−*^ HCT116 cells, we found that ASNS expression was significantly augmented in *p53*^*−/−*^ cells (Fig. 2b, c). Similar findings were observed in U2OS cells (Fig. 2b, c). Conversely, the forced expression of p53 reduced ASNS mRNA levels (Supplementary Fig. 3a). The pharmacological activation of p53 by nutlin-3 decreased ASNS expression (Fig. 2d and Supplementary Fig. 3b, c), and the suppression of p53 by using PFT-α elevated ASNS expression (Supplementary Fig. 3b). This p53-dependent repression was illustrated by the abrogation of ASNS expression following nutlin-3 treatment in p53-null cells (Fig. 2d and Supplementary Fig. 3c). DNA damage signals such as etoposide (ETO) and doxorubicin (DOX) can stabilise p53 protein. Treatment with ETO decreased ASNS expression in *p53*^*+/+*^ cells but not in *p53*^*−/−*^ cells (Supplementary Fig. 3d). Similarly, supplying EL4 cells with Nutlin-3, ETO or DOX resulted in a reduction in ASNS expression, whereas PFT-α treatment increased it (Fig. 2e).

To both elucidate whether the p53-mediated regulation of ASNS is cell type specific and further confirm the specificity of the effect observed with p53 depletion at the basal level, we knocked down p53 in a variety of cell lines expressing endogenous wildtype p53 or mutant p53. The silencing of p53 increased ASNS expression in wildtype cell lines but not in mutant cells (Supplementary Fig. 3e, f). In keeping with this, nutlin-3 treatment failed to alter the expression of ASNS in p53-mutated cell lines (Supplementary Fig. 3g). p53A138V is a tumour-associated, temperature-sensitive mutant p53 (p53-ts) that exhibits wildtype p53 activity at 32 °C but induces transformation at 37 °C^18^. The expression of p53A138V inhibited *ASNS* transcription at the permissive temperature of 32 °C but not at 37 °C (Supplementary Fig. 3h). Taken together, the results show that p53 mutant tumour cells lack the ability to suppress ASNS expression.

ASNS is expressed in various mouse tissues (Supplementary Fig. 3i). Consistent with the observations that plasma from *p53*^*−/−*^ mice had higher levels of asparagine (Fig. 1c), multiple tissues from *p53*^*−/−*^ mice had higher levels of ASNS expression than did those from *p53*^*+/+*^ mice (Fig. 2f and Supplementary Fig. 3j). Intriguingly, no significant enhancement in ASNS mRNA levels was observed in bone marrow from *p53*^*−/−*^ mice, despite a significant increase in ASNS protein (Fig. 2f and Supplementary Fig. 3j), indicating the existence of tissue-specific and transcription-independent mechanism(s) for the regulation of ASNS expression by p53. Nevertheless, these data suggest that ASNS is a physiological target for p53, and its expression is suppressed by p53 at both the genotoxic stress and basal levels.

We next investigated the mechanism for the regulation of ASNS expression by p53. By analysing the *ASNS* gene sequence for potential p53 protein response elements, which share the consensus sequence of 5′-RRRCWWGYYY-(0-13 base pair (bp) spacer)-RRRCWWG YYY-3′ (where R is a purine, Y a pyrimidine, and W an A or T; ref. ^19^), we identified three putative p53 response elements in the *ASNS* gene (Supplementary Fig. 3k). Chromatin immunoprecipitation (ChIP) assays revealed that p53 bound to all these response element regions (Fig. 2g). Moreover, p53 repressed the expression of a luciferase gene driven by a genomic fragment containing these response elements (*ASNS*-RE1, *ASNS*-RE2, or *ASNS*-RE3, Supplementary Fig. 3l). Together, these results suggest that p53 binds to the *ASNS* gene and suppresses ASNS expression.

### p53 regulates Asn-Asp homeostasis

Next, we investigated whether p53 regulates asparagine metabolism by ASNS (Fig. 2a). Notably, the lack of p53 resulted in increased ASNS activities (Fig. 2h). Moreover, intracellular asparagine levels were significantly augmented in p53-depleted cells (Fig. 2i), consistent with the observations that p53 loss enhances extracellular asparagine levels (Fig. 1h and Supplementary Figs. 2i and 4a). In line with the expression data (Fig. 2e), pharmacological treatment of EL4 cells with p53 activators led to decreased intracellular asparagine (Fig. 2j). However, we found that extracellular asparagine levels varied, which could be attributed to the potential side effects of these compounds on asparagine transportation (Supplementary Fig. 4b). Nevertheless, cells treated with PFT-α displayed enhanced asparagine production (Fig. 2j and Supplementary Fig. 4b). p53 loss increased intracellular and extracellular asparagine (Figs. 1h and 2i and Supplementary Figs. 2i and 4a). In contrast, aspartate both within and outside cells declined significantly when p53 was absent (Fig. 2k).

Next, we directly assessed the effect of p53 on natural asparagine synthesis derived from aspartate. Three cell lines (EL4, HCT116 and U2OS) were cultured in medium containing ^15^N-aspartate, and a portion of intracellular ^15^N-aspartate and ^15^N-asparagine was found, suggesting that aspartate could be taken up and converted to asparagine by these cells (Fig. 2l and Supplementary Fig. 4c, d). Remarkably, isotope tracing using ^15^N-aspartate showed that p53 deficiency enhanced asparagine synthesis from aspartate (Fig. 2m), further confirming the data that a lack of p53 enhances ASNS expression and activity (Fig. 2b–h and Supplementary Fig. 3a–f). Similar findings were obtained in cells cultured with ^13^C-labelled glutamine ([U-^13^C_5_]Gln), which could support Asn synthesis by providing carbons through the TCA cycle. While p53 depletion caused an overall decline in aspartate levels (Fig. 2k), cellular [U-^13^C_5_]Gln-derived aspartate increased when p53 was absent (Fig. 2n). Nevertheless, a significant increase in ^13^C-asparagine was found in p53-deficient cells (Fig. 2o).

To verify the cell culture findings in animals, we compared the asparagine levels in tissues from *p53*^*+/+*^ mice and *p53*^*−/−*^ mice. Consistent with the plasma data (Fig. 1c, Supplementary Fig. 1c and h), higher levels of asparagine were found in liver and pancreas tissues from *p53*^*−/−*^ mice than in those from *p53*^*+/+*^ mice (Fig. 2p). These data together demonstrate that p53 regulates ASNS-mediated asparagine-aspartate homeostasis.

### ASNS promotes tumour cell proliferation through Asn

**Fig. 3:**
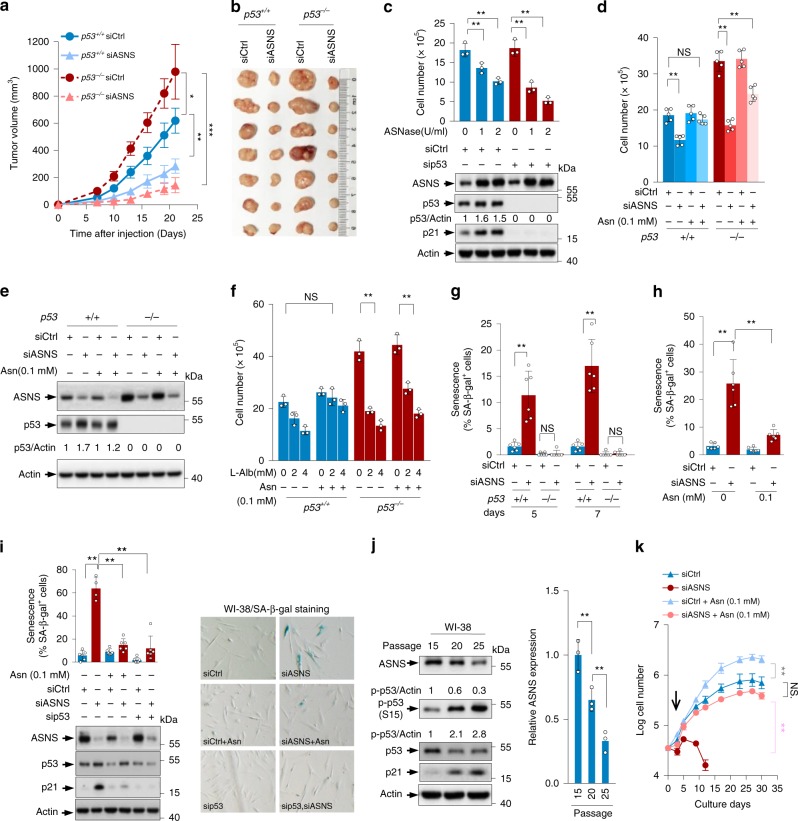
ASNS modulates cell proliferation and senescence via asparagine. **a**, **b** *p53*^*+/+*^ and *p53*^*−/−*^ HCT116 cells expressing control or ASNS siRNA were injected subcutaneously into the dorsal flanks of twenty-eight nude mice. Tumour volumes (*n* = 7 mice in each group) were measured every 3 days after the tumours were visible (**a**). Representative images of xenografted tumours are shown (**b**). **c** HCT116 cells transfected with control or p53 siRNA were treated with increasing amounts of ASNase (0, 1 or 2 U per ml) for 48 h. Cell proliferation and protein expression were analysed. **d**, **e** *p53*^*+/+*^ and *p53*^*−/−*^ HCT116 cells transfected with control or ASNS siRNA were cultured with or without 0.1 mM Asn for 3 days. Proliferation (**d**) and protein expression (**e**) were measured. **f** Proliferation of *p53*^*+/+*^ and *p53*^*−/−*^ HCT116 cells treated with 0, 2 or 4 mM l-albizziine (l-Alb) or 0.1 mM Asn as indicated for 48 h. **g** Percentages of senescence-associated β-galactosidase (SA-β-gal)-positive *p53*^*+/+*^ and *p53*^*−/−*^ HCT116 cells transfected with control or ASNS siRNA for 5 or 7 days. **h** HCT116 cells transfected with control or ASNS siRNA were cultured in medium with or without 0.1 mM Asn for 5 days. Percentages of SA-β-gal-positive cells are shown. **i** WI-38 cells transfected with control, ASNS siRNA and/or p53 siRNA were cultured for 3 days in the presence or absence of 0.1 mM Asn as indicated. Representative images, percentages of SA-β-gal-positive cells and protein expression are shown. **j** Protein expressions in WI-38 cells at different passages were measured. **k** The replicative lifespan of WI-38 cells treated with control, ASNS siRNA and/or 0.1 mM Asn as indicated. Arrow indicates the onset of senescence. Data are mean ± s.d., unpaired two-tailed Student’s *t*-test, *p < 0.05, **p < 0.01, ***p < 0.001, NS not significant. Source data are provided as a Source Data file.

The expression of ASNS is ultimately associated with the resistance of leukaemia cells and leukaemic lymphoblasts to asparagine depletion. However, the physiological effect of ASNS on other somatic tumour cells remains largely unknown. As shown in Supplementary Fig. 5a–c, HCT116 cell proliferation was blocked when ASNS was knocked down. In addition, ASNS depletion exerted a more profound effect on *p53*^*−/−*^ cells than on wildtype control cells. Likewise, the depletion of ASNS impeded the growth of tumours derived from *p53*^*+/+*^ and *p53*^*−/−*^ HCT116 cells (Fig. 3a, b), suggesting that ASNS is important for tumour growth. Similarly, the removal of asparagine from the cell medium by adding ASNase remarkably reduced the proliferation of both p53 knockdown HCT116 cells and their control counterparts (Fig. 3c). Analogously, siRNA-mediated silencing of ASNS led to proliferation arrest (Fig. 3d). The underlying mechanism seems to be mediated by asparagine because the readdition of asparagine restored cell proliferation (Fig. 3d, e). Similar results were obtained using l-albizziine (l-Alb), a competitive inhibitor of ASNS. Treatment with l-Alb led to a dose-dependent inhibition of the proliferation of *p53*^*+/+*^ and *p53*^*−/−*^ cells. Asparagine addition was sufficient to restore the proliferation of *p53*^*+/+*^ cells and partially restore that of *p53*^*−/−*^ cells (Fig. 3f). Moreover, the silencing of ASNS reduced anchorage-independent tumour cell growth, while supplementation with asparagine promoted overall tumour cell growth and almost restored the numbers of *p53*^*+/+*^ colonies, with some restoration of *p53*^*−/−*^ colonies (Supplementary Fig. 5d). The failure of exogenous asparagine to restore the proliferation of ASNS-depleted *p53*^*−/−*^ cells does not seem to be attributed to the low capability of *p53*^*−/−*^ cells to take in environmental asparagine because supplementation with 0.1 mM asparagine sufficiently restored intracellular asparagine in both *p53*^*+/+*^ cells and *p53*^*−/−*^ cells with ASNS knockdown (Supplementary Fig. 5e). Furthermore, when cells were cultured with ^15^N-asparagine, ASNS silencing resulted in higher levels of intracellular ^15^N-asparagine in *p53*^*−/−*^ cells than in *p53*^*+/+*^ cells, and correspondingly, much lower levels of ^15^N-asparagine outside *p53*^*−/−*^ cells were found (Supplementary Fig. 5f). Therefore, the lack of ASNS suppresses *p53*^*−*/*−*^ cell proliferation through both asparagine-dependent and asparagine-independent mechanisms. Notably, when ASNS was present, *p53*^*−/−*^ cells had lower levels of ^15^N-asparagine than did *p53*^*+/+*^ cells (Supplementary Fig. 5f), suggesting that, under this condition, *p53*^*−/−*^ cells are more dependent on the de novo synthesis of asparagine, correlating with the findings that p53 depletion elevated ASNS expression (Fig. 2b–e and Supplementary Fig. 3). Taken together, these findings suggest that ASNS supports cell proliferation in a p53 context-dependent manner through asparagine.

### ASNS modulates p53-dependent cell senescence via Asn

p53 is a critical regulator of senescence. We noticed that ASNS silencing increased the number of cells expressing senescence-associated β-galactosidase in *p53*^*+/+*^ cells but not *p53*^*−/−*^ cells (Fig. 3g and Supplementary Fig. 5g). Notably, this ASNS knockdown-induced senescence could be reversed by asparagine treatment (Fig. 3h and Supplementary Fig. 5h). Similar results were obtained in the primary human diploid WI-38 cell line. ASNS knockdown caused a profound increase in the percentages of senescent cells in control siRNA-treated WI-38 cells but had little effect on cells without p53 (Fig. 3i). Again, asparagine addition reduced cell senescence (Fig. 3i).

We next further examined the effect of asparagine on other scenarios of p53-mediated senescence. Interestingly, asparagine addition inhibited the p53-dependent senescence induced by glutamine starvation (Supplementary Fig. 5i). In WI-38 cells, ASNS expression declined as senescence progressed, especially at the late stage (Fig. 3j). To test whether the decline in ASNS contributes to replicative senescence, we examined the replicative capacity of WI-38 cells treated with or without ASNS siRNA and/or asparagine. Compared with control cells, which could be cultured for extended passages, ASNS-depleted cells exhibited a greatly accelerated onset of senescence (Fig. 3k). Strikingly, the addition of asparagine was sufficient to delay senescence and could almost completely reverse the acceleration of senescence induced by ASNS depletion (Fig. 3k).

To elucidate the senescence-associated function of asparagine in the context of oncogenic signals, we investigated the effect of asparagine on HrasV12-induced premature senescence^20^. Although p53 depletion largely abolished HrasV12-induced senescence in U2OS cells (Supplementary Fig. 5j, k), asparagine did not (Supplementary Fig. 5j, l). Taken together, these results indicate that ASNS regulates p53-dependent, but not oncogene-induced, senescence through asparagine.

### ASNS and Asn maintain tumour cell survival

**Fig. 4:**
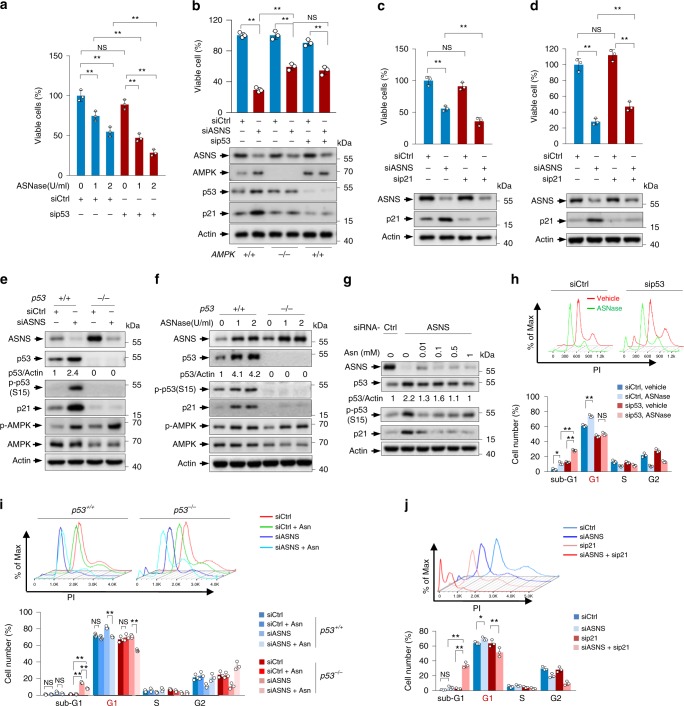
Asparagine maintains cell survival, and represses p53-dependent cell cycle checkpoint activation. **a** HCT116 cells transfected with control or p53 siRNA were cultured with 0, 1 or 2 U per ml ASNase for 48 h. Cell survival (% of viable cells) was determined by trypan blue exclusion assay. **b** Survival of and Protein expression in MEFs transfected with control, p53 siRNA and/or ASNS siRNA as indicated. **c**, **d** Survival of and protein expression in HCT116 cells (**c**) and MEFs (**d**) transfected with control, ASNS siRNA and/or p21 siRNA as indicated. **e** Protein levels of p53, p21 and phosphorylated p53 (S15) in *p53*^*+/+*^ and *p53*^*−/−*^ HCT116 cells transfected with control or ASNS siRNA for 3 days. **f** Protein expression in *p53*^*+/+*^ and *p53*^*−/−*^ HCT116 cells treated with 0, 1 or 2 U per ml ASNase for 48 h was analysed. **g** Protein expression in siCtrl and siASNS HCT116 cells cultured in medium containing increasing amounts of Asn for 3 days was analysed. **h** Cell cycle distribution of siCtrl and sip53 HCT116 cells treated with 0 or 2 U/ml ASNase for 48 h. The percentages of cells in the sub-G1, G1, S and G2 phases were determined by PI staining and flow cytometry analysis. **i** *p53*^*+/+*^ and *p53*^*−/−*^ HCT116 cells transfected with control or ASNS siRNA were cultured in medium containing 0 or 0.1 mM Asn for 3 days. Cell cycle distribution was determined. **j** Cell cycle distribution of HCT116 cells transfected with control, ASNS siRNA and/or p21 siRNA for 72 h. Data are mean ± s.d., unpaired two-tailed Student’s *t*-test, *p < 0.05, **p < 0.01, ***p < 0.001, NS not significant. Source data are provided as a Source Data file.

To gain further insight into the functions of asparagine in cell survival, we studied the effect of asparagine and ASNS on cell survival. ASNase treatment reduced the survival of both *p53*^*+/+*^ and *p53*^*−/−*^ HCT116 cells (Fig. 4a). These effects were also found in other tumour cell lines (Supplementary Fig. 6a–c). Similarly, l-Alb-mediated inhibition of ASNS diminished the survival of these cells (Supplementary Fig. 6b, c). Consistent with these observations in tumour cells, when primary MEFs were treated with ASNS siRNA, cell survival also declined, reflecting a physiological relevant effect of asparagine on cell survival (Fig. 4b).

We noticed that in the HCT116 cell line, ASNase treatment decreased the survival of sip53 cells compared to the control cells (Fig. 4a). In contrast, in the other cell lines examined, p53 depletion correlated with better cell survival in response to ASNase or ASNS siRNA treatment (Fig. 4b and Supplementary Fig. 6a, b). This discrepancy might be due to the higher expression of p21 in HCT116 cells (Supplementary Fig. 6d), as p21 depletion further decreased the survival of HCT116 cells, not MEFs and HepG2 cells (Fig. 4c, d and Supplementary Fig. 6e). Taken together, these findings demonstrate that asparagine maintains cell survival.

### ASNS impairs p53-dependent cell cycle arrest and apoptosis

Failure to activate the cell cycle checkpoint impairs senescence and induces abnormal proliferation or apoptosis under certain stress conditions^12,13,21^. The above findings that the alteration of asparagine metabolism influences various p53-dependent cell physiological events led us to ascertain the effect of ASNS/asparagine on cell cycle progression. As shown in Fig. 4e, silencing ASNS resulted in p53 activation, as indicated by increased p53 phosphorylation (ser-15) and p21 expression. Similar results were obtained in U2OS cells (Supplementary Fig. 7a). Likewise, treatment with ASNase remarkably activated p53 (Fig. 4f and Supplementary Fig. 7b). The induction of p21 expression was p53 dependent, as this effect was abrogated when p53 was absent (Fig. 4e, f and Supplementary Fig. 7a, b). To investigate whether these effects were mediated by asparagine, we added increasing amounts of asparagine to the medium of ASNS-KD cells. Notably, asparagine addition reduced the levels of p53 phosphorylation and p21 expression (Fig. 4g). Moreover, l-Alb treatment triggered p53 activation, and asparagine supplementation almost completely reversed it (Supplementary Fig. 7c). Additionally, asparagine addition could also reverse the enhancement of p53 phosphorylation and p21 expression induced by glutamine starvation, a metabolic stress that can induce a p53-dependent checkpoint for cell survival^15^ (Supplementary Fig. 7d). Together, these data demonstrate a physiological function of asparagine in suppressing p53.

We next investigated whether a p53/p21-dependent cell cycle checkpoint is initiated upon asparagine depletion. Indeed, in HCT116 cells, ASNase treatment increased the numbers of p53 wildtype cells in the G1 phase. In contrast, the number of p53-depleted cells in this phase was almost unaffected by ASNase treatment; instead, more sip53 HCT116 cells were in sub-G1 phase (apoptosis) than were their wildtype counterparts (Fig. 4h and Supplementary Fig. 11b). Likewise, in p53-expressing U2OS cells and HepG2 cells, ASNase supplementation provoked G1 arrest, whereas the depletion of p53 totally abrogated this effect and induced increased the number of cells in sub-G1 phase (Supplementary Figs. 7e, f and 11b). This effect of ASNase on apoptosis was further confirmed by flow cytometry (FACS) using Annexin V-FITC/PI staining. After 48 h of ASNase treatment, ~20% of p53-depleted cells and ~6% of control cells underwent apoptosis (Supplementary Figs. 7g and 11c).

Consistent with the ASNase data (Fig. 4h and Supplementary Fig. 7e, f), the knockdown of ASNS in multiple tumour cell lines substantially increased G1 arrest in wildtype p53-expressing cells but not in p53-depleted cells. Instead, higher percentages of p53-depleted cells in sub-G1 were found (Fig. 4i and Supplementary Fig. 8a–c). Intriguingly, in MEFs, silencing ASNS increased G1 arrest, yet no significant increase in the sub-G1 population of sip53 MEFs was observed (Supplementary Fig. 8d), indicating that p53 loss renders tumour cells more susceptible to ASNS depletion.

Nevertheless, asparagine addition abolished G1 arrest in *p53*^*+/+*^ cells and reduced the percentage of *p53*^*−/−*^ cells in sub-G1 phase (Fig. 4i). In accordance with the findings that asparagine removal induced p53/p21-dependent cell cycle arrest, ASNS knockdown failed to induce G1 arrest but increased the percentages of cells in sub-G1 when p21 was depleted (Fig. 4j). Moreover, similar to p53 deficiency, silencing p21 increased the apoptosis induced by ASNS siRNA (Supplementary Fig. 8e). Together, these data demonstrate that ASNS/asparagine depletion induces p53-dependent cell cycle arrest to protect cells from apoptosis.

### ASNS and Asn regulate p53 activity via AMPK

**Fig. 5:**
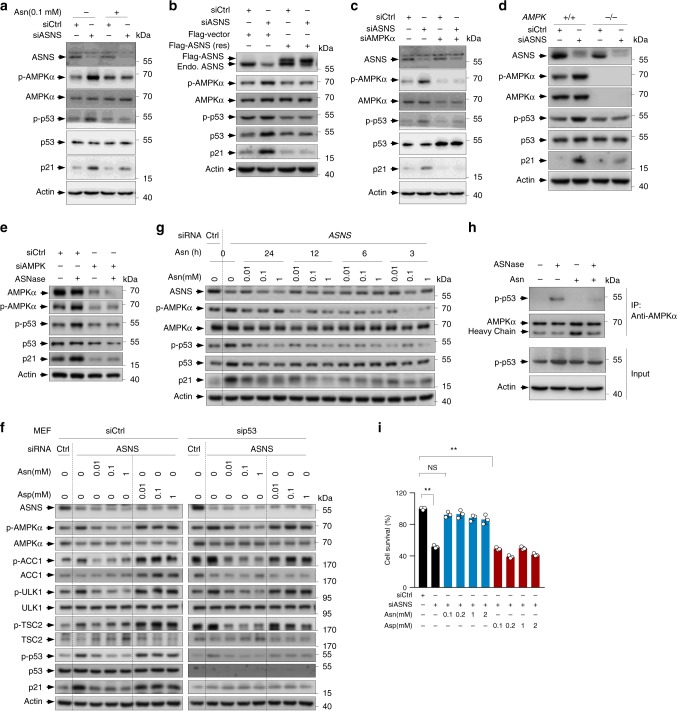
ASNS and asparagine regulate p53 through AMPK. **a** HCT116 cells transfected with control or ASNS siRNA were cultured in medium containing 0 or 0.1 mM Asn for 3 days. Protein expression was analysed by western blotting. **b** U2OS cells stably expressing Flag-tagged RNAi-resistant (res) ASNS or Flag vector control were transfected with control or ASNS siRNA for 72 days. Protein expression was analysed. **c** Expression of the indicated proteins in HCT116 cells transfected with control, ASNS siRNA and/or AMPKα siRNA as indicated for 3 days. **d** Western blot analysis of lysates from *AMPK*^*+/+*^ and *AMPK*^*−/−*^ MEFs transfected with control or ASNS siRNA as indicated for 3 days. **e** HCT116 cells transfected with control or AMPK siRNA were cultured in medium containing 0 or 2 U per ml ASNase for 48 h. Protein expression was analysed. **f** MEFs transfected with control, p53 siRNA or ASNS siRNA were treated with or without increasing amounts of Asn or aspartate Asp as indicated. Protein expression is shown. **g** MEFs transfected with control or ASNS siRNA were treated with or without increasing amounts of Asn for the indicated time points. The expression of the indicated proteins was analysed by western blotting. **h** HCT116 cells cultured in control medium or medium containing ASNase and/or 0.1 mM asparagine as indicated for 48 h. Cells were harvested and subjected to immunoprecipitation using anti-AMPK antibody, and bound proteins were analysed by western blotting. **i** MEFs transfected with control or ASNS siRNA were treated with or without increasing amounts of Asn or Asp as indicated. Relative cell viability (%) is shown. Data are mean ± s.d., unpaired two-tailed Student’s *t*-test, *p < 0.05, **p < 0.01, ***p < 0.001, NS not significant. Source data are provided as a Source Data file.

We next examined the mechanism by which asparagine regulates p53. AMP-activated protein kinase (AMPK) is an intracellular energy-sensing kinase that can activate p53 through phosphorylation^14,22^. Interestingly, ASNase treatment elevated AMPK phosphorylation in both *p53*^*+/+*^ and *p53*^*−/−*^ HCT116 cells (Fig. 4f) and U2OS cells expressing p53 or control shRNA (Supplementary Fig. 7b). Similarly, when ASNS was knocked down, AMPK was activated, as evidenced by the increase in phosphorylation of AMPK and its substrate ACC1 (Figs. 4e and 5a and Supplementary Figs. 7a and 9a). Moreover, the expression of RNAi-resistant ASNS almost completely abolished AMPK activation in ASNS-knockdown cells (Fig. 5b). Based on these findings, we tested whether AMPK is required for ASNS silencing-triggered p53 activation by knocking down AMPK in HCT116 cells and by comparing AMPK null and wildtype MEFs. In both situations, the loss of AMPK expression prevented ASNS depletion from activating p53 (Fig. 5c, d). Similarly, AMPK silencing attenuated ASNase-induced p53 phosphorylation (Fig. 5e).

To directly assess whether asparagine is involved in ASNS depletion-mediated AMPK activation, we cultured l-Alb-treated EL4 cells in medium containing asparagine or aspartate as a control. Interestingly, supplying cells with asparagine lessened the AMPK phosphorylation induced by l-Alb treatment (Supplementary Fig. 9b). Analogously, asparagine addition led to the decreased phosphorylation of AMPK and p53 in ASNS-depleted cells (Fig. 5a, f, g and Supplementary Fig. 9d), and this phenomenon could be observed shortly after asparagine treatment (Fig. 5g). Consistent with these findings, ASNase supplementation increased the level of AMPK-bound phosphorylated p53, whereas supplying cells with asparagine reversed this effect (Fig. 5h), suggesting that asparagine suppresses AMPK activity towards p53.

Aspartate supplementation increased overall cellular aspartate levels, particularly in ASNS-deprived cells (Supplementary Fig. 9c). Surprisingly, aspartate treatment did not reduce AMPK and p53 activation but instead, to some extent, activated AMPK (Fig. 5f and Supplementary Fig. 9d). Similar findings were obtained when cells were cultured in glutamine-free medium (Supplementary Fig. 9e). Consistently, asparagine addition restored cell survival in siASNS cells, while aspartate supplementation did not (Fig. 5i). Taken together, the results show that ASNS and asparagine regulate p53 through AMPK.

### Asn-Asp homeostasis dictates AMPK activity

**Fig. 6:**
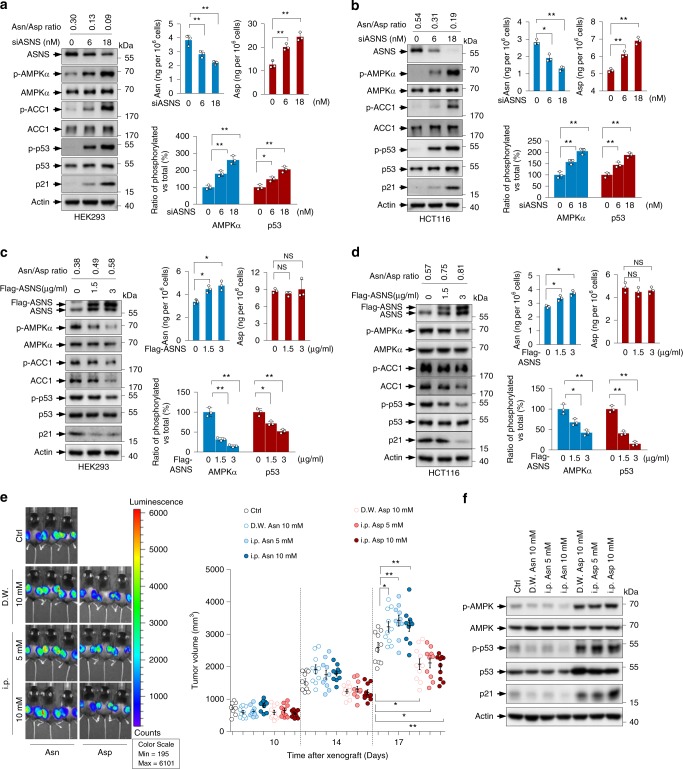
Changes in asparagine-aspartate homeostasis alter AMPK signalling. **a**, **b** HEK293 cells (**a**) and HCT116 cells (**b**) were transfected with increasing amounts of ASNS siRNA for 3 days. Cellular Asn and Asp were determined and quantified by LC-MS. The ratios of Asn/Asp are shown (top left panel). Protein expression was determined (left panel), and the ratios of phosphorylated AMPK, total AMPK, phosphorylated p53 and total p53 are shown (bottom right panel). **c**, **d** HEK293 cells (**c**) and HCT116 cells (**d**) were transfected with increasing amounts of Flag-ASNS plasmids as indicated for 3 days. Cellular Asn and Asp were determined and quantified by LC-MS (top right panel). The ratios of Asn/Asp (top left panel) and protein expression (left panel), and the ratios of phosphorylated AMPK, total AMPK, phosphorylated p53 and total p53 are shown (bottom right panel). **e**, **f** C57BL/6 J mice xenografted s.c. with 5 × 10^5^ EL4-Luc-GFP cells were administered vehicle, Asn or Asp by either oral absorption (from drinking water, D.W.) or intraperitoneal injection (i.p.) of 500 μl every two days as indicated. The whole-mouse bioluminescence analysis was performed at 18 days post-injection. **e** Representative images of the mice are shown. The colour scale represents the intensity of the emitted luminescence. Each group includes 5 mice. Tumour volumes were measured by a calliper at 10, 14 and 18 days post-xenograft. Each group includes 5 mice and 10 tumours. **f** Expression levels of AMPK, p-AMPK, p53, p-p53 and p21 in xenografted tumours were analysed by western blotting. Data are mean ± s.d., unpaired two-tailed Student’s *t*-test, *p < 0.05, **p < 0.01, ***p < 0.001, NS not significant. Source data are provided as a Source Data file.

Cellular aspartate is in equilibrium with asparagine. We therefore investigated the effect of the dynamic asparagine-aspartate ratio on AMPK and p53. Targeting ASNS by siRNA resulted in a decrease in the levels of cellular asparagine and a strong accumulation of aspartate, leading to a steep decline in the ratio of asparagine-aspartate (Fig. 6a, b). Consistent with this, AMPK and p53 were activated (Fig. 6a, b), which negatively correlated with the decreased asparagine-aspartate ratio. Similarly, lowering the asparagine-aspartate ratio by l-Alb correlated with increased AMPK and p53 activation (Supplementary Fig. 9f). In contrast, the forced expression of ASNS induced asparagine synthesis, and intriguingly, aspartate levels did not decline under these conditions (Fig. 6c, d). The failure of ASNS overexpression to affect aspartate amounts might be due the compensation of other pathways (such as alanine metabolism and the TCA cycle) that can produce aspartate. Nevertheless, the ratio of asparagine and aspartate remained significantly increased in a dose-dependent manner. Consistently, we found a dose-dependent reduction in the activation of AMPK and p53 under these conditions (Fig. 6c, d). Together with the findings that p53 suppresses ASNS, our findings may reveal a positive feedback loop between p53 and asparagine metabolism: the activation of p53 lessens ASNS expression, reduces asparagine-aspartate ratio and turns on AMPK, consequently leading to even higher p53 activation.

Asparagine addition was sufficient to block AMPK activation (Fig. 5a, f, g and Supplementary Fig. 9b, d). Next, we wanted to determine the effect of aspartate on AMPK-p53 signalling. In contrast to asparagine, the addition of aspartate stimulated AMPK signalling (Supplementary Fig. 9g, h). This effect was largely abolished when AMPK was absent (Supplementary Fig. 9h). In accordance with these observations, aspartate supplementation reduced cell proliferation (Supplementary Fig. 9i). Intriguingly, aspartate treatment elevated total AMPK levels in MEFs (Supplementary Fig. 9g, h), which is unlikely due to aspartate-mediated AMPK phosphorylation by LKB1 because AMPK phosphorylation rarely correlates with its protein stabilisation.

Next, we extended our studies to animals. Mice receiving asparagine through either intraperitoneal injection or oral consumption via drinking water had increased sizes of tumours derived from EL4 cells (Fig. 6e). In line with this observation, AMPK and p53 phosphorylation declined in these tumours (Fig. 6f). In contrast, aspartate administration impeded tumour growth (Fig. 6e) and activated AMPK and p53 (Fig. 6f). These results were strengthened by the findings that body weight remained unchanged (Supplementary Fig. 9j), and plasma asparagine and aspartate correspondingly increased following injection or oral administration (Supplementary Fig. 9k). Taken together, our findings reveal that LKB1-AMPK signalling may be the predominant mechanism by which asparagine and aspartate regulate p53.

### Asn and Asp directly bind to LKB1 and modulate its activity

**Fig. 7:**
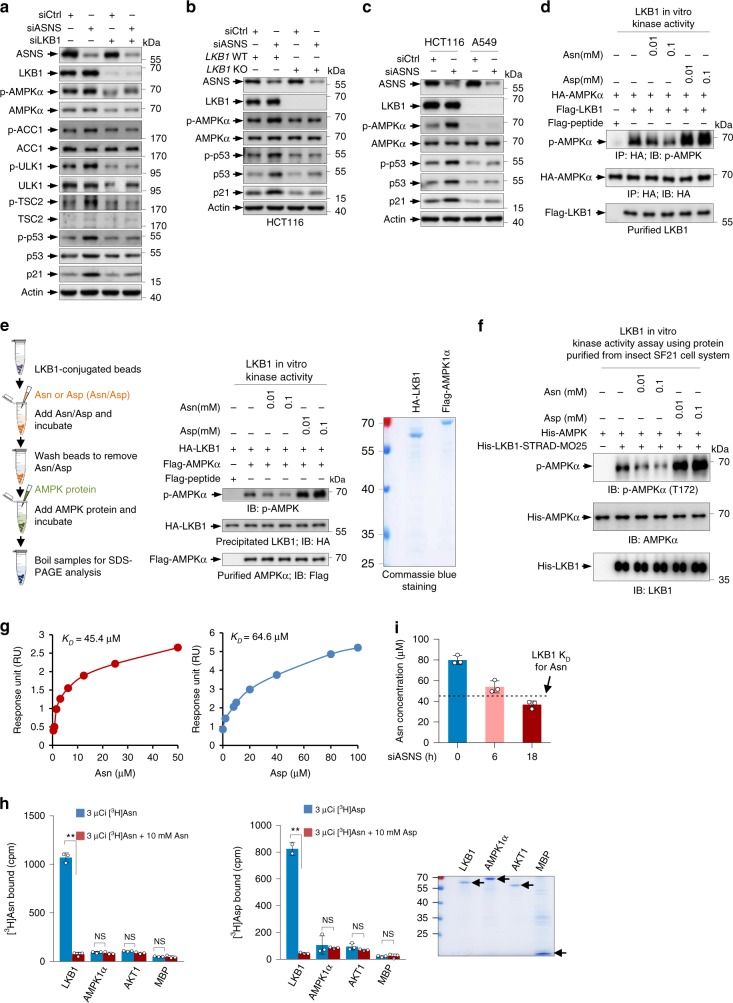
LKB1 is a natural sensor for Asn and Asp. **a** Protein expression in U2OS cells transfected with Control, ASNS siRNA and/or LKB1 siRNA as indicated. **b** Protein expression in LKB1 wildtype (WT) and knockout (KO) HCT116 cells treated with siCtrl or siASNS as indicated. **c** Western blot analysis of lysates from HCT116 cells and A549 cells transfected with control or ASNS siRNA. **d** In vitro LKB1 kinase activity assay using HEK-293 cell-purified proteins as indicated in the presence or absence of Asn or Asp at 30 °C for 30 min. **e** HEK-293 cell-Purified HA-LKB1 immobilised on anti-HA agarose beads (LKB1 beads) was stimulated by incubating with Asn or Asp for 30 °C for 30 min, and then unbound Asn/Asp was removed by washing and further incubated with LKB1 beads with purified Flag-AMPK protein for another 30 °C for 30 min. AMPK phosphorylation was determined. **f** In vitro LKB1 kinase activity was determined using SF21 cell-purified His-LKB1-STRAD-MO25 complex and His-AMPKα in the presence or absence of Asn or Asp at 30 °C for 30 min. **g** Surface plasmon resonance (BIAcore) measurement of the interaction between purified LKB1 and Asn (left) or Asp (right). Graphs of equilibrium response units (RU) and compound concentrations are shown. The estimated *K*_D_ values were 45.4 µM and 64.4 µM. **h** Binding of radiolabelled [^3^H]Asn and [^3^H]Asp to HEK-293 cell-purified proteins (see Methods for details). Unlabelled amino acids were added where indicated. **i** HCT116 cells were treated with ASNS siRNA for 0, 6 and 18 h as indicated before sample preparation for LC/MS-based analysis of the absolute amounts of asparagine. The dissociation constant *K*_D_ of LKB1 for asparagine is indicated. Data are mean ± s.d., unpaired two-tailed Student’s *t*-test, *p < 0.05, **p < 0.01, ***p < 0.001, NS not significant. Source data are provided as a Source Data file.

Next, we investigated the mechanism(s) by which asparagine and aspartate regulate AMPK. AMPK is regulated by LKB1-mediated phosphorylation^22,23^. Interestingly, ASNS depletion-induced AMPK activation (indicated by the increased phosphorylation of its substrates ACC1, ULK1, TSC2 and p53) was abrogated when LKB1 was knocked down (Fig. 7a and Supplementary Fig. 10a). Similar results were observed in LKB1-knockout cells (Fig. 7b). Analogously, the knockdown of ASNS failed to activate AMPK in LKB1-deficient A549 cells (Fig. 7c). Collectively, these findings suggest that LKB1 mediates the regulation of AMPK by ASNS.

We next investigated whether asparagine and/or aspartate act(s) directly on LKB1. When incubated with HEK293 cell-purified LKB1, asparagine dose-dependently abrogated LKB1 activity, while aspartate considerably augmented it (Fig. 7d). Similar results were obtained using Myelin Basic Protein (MBP), another LKB1 substrate (Supplementary Fig. 10b). In contrast, when LKB1 was absent, asparagine and aspartate did not affect AMPK and MBP phosphorylation (Supplementary Fig. 10c). To further verify these findings, we performed a sequential stimulation assay (Fig. 7e, left panel). HEK293 cell-purified LKB1 protein was immobilised on agarose beads and stimulated by incubation with asparagine or aspartate (Asn/Asp). After washing the beads to eliminate unbound Asn/Asp, the Asn/Asp-stimulated LKB1 (on beads) was further incubated with AMPK protein for the AMPK phosphorylation assay. Again, asparagine stimulation reduced LKB1 activity, whereas aspartate increased it (Fig. 7e). We next used insect SF21 cell-purified proteins to further confirm these findings. Likewise, asparagine reduced AMPK phosphorylation in the presence of LKB1, while aspartate enhanced LKB1-mediated AMPK phosphorylation, suggesting that asparagine and aspartate directly regulate LKB1 activity (Fig. 7f and Supplementary Fig. 10d).

The binding of AMPK to AMP causes conformational changes that promote the LKB1-mediated phosphorylation of AMPK at Thr172^14,22,24^. However, the addition of either asparagine or aspartate showed no effect on the LKB1-AMPK interaction in vitro (Supplementary Fig. 10e). Similarly, changing the cellular asparagine-aspartate ratio by knocking down ASNS only slightly enhanced the LKB1-AMPK interaction (Supplementary Fig. 10f, g). These data indicate that asparagine and aspartate may directly act on LKB1. Indeed, a real-time binding assay using surface plasmon resonance (SPR) (BIAcore) showed that both asparagine and aspartate were able to directly bind to LKB1 with a dissociation constant (K_D_) of 45.5 μM and 64.6 μM, respectively (Fig. 7g and Supplementary Fig. 10h). Moreover, an equilibrium binding assay^25^ using radioactive [^3^H]Asn revealed that [^3^H]Asn specifically bound to LKB1 but not AMPK, AKT1 or MBP. The binding between [^3^H]Asn and LKB1 proteins could be fully competed by excess nonradiolabelled Asn (Fig. 7h). Likewise, Asp bound to LKB1 but not to AMPK, AKT1 or MBP (Fig. 7h). In contrast to Asn and Asp, other amino acids used as controls, such as methionine (Met), leucine (Leu), glycine (Gly), alanine (Ala) and glutamate (Glu), did not bind to LKB1 (Supplementary Fig. 10i). Importantly, while ASNS silencing to some extent increased cellular aspartate levels, cellular asparagine concentrations decreased significantly upon ASNS siRNA treatment, decreasing from above the dissociation constant of LKB1 for asparagine to below it (Fig. 7i and Supplementary Fig. 10j), indicating that asparagine is a physiological modulator of LKB1 activity. Taken together, our findings suggest that asparagine and aspartate directly bind to LKB1 to oppositely modulate its activity.

## Discussion

In this work, we found that some tumour cells, in particular p53-null cells, have active ASNS expression and asparagine synthesis. Increased asparagine production helps cells proliferate and protects from senescence. Interestingly, ASNase treatment or ASNS knockdown reciprocally activates p53 to induce cell cycle arrest and protect cells from apoptosis, suggesting a therapeutic approach (e.g. use of ASNase) for the treatment of p53-null tumours by interfering with asparagine synthesis.

**Fig. 8:**
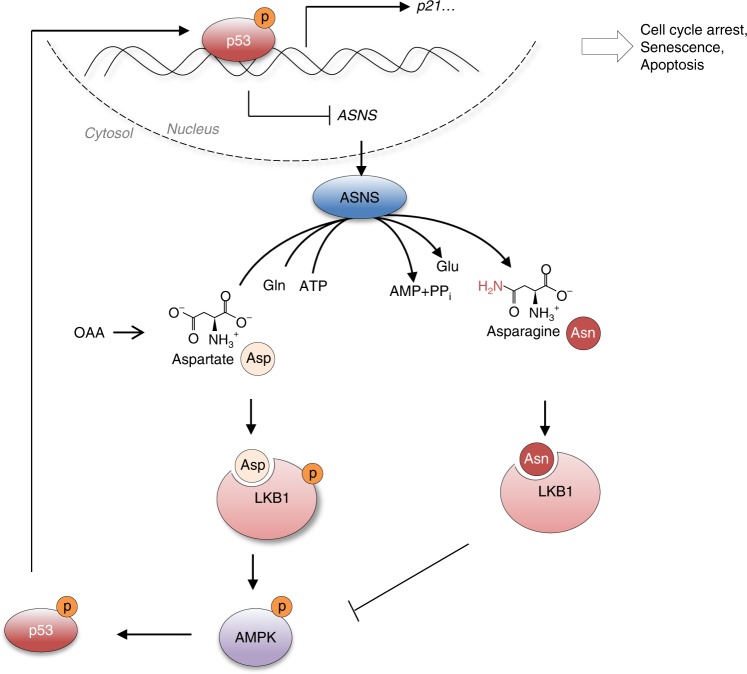
A model of the reciprocal regulation of p53 and ASNS in modulating metabolism and cell states that illustrates LKB1 as a natural sensor for Asp-Asn homeostasis. Briefly, p53 modulates asparagine metabolism and aspartate-asparagine homeostasis through transcriptionally repressing ASNS expression. Moreover, accumulation of asparagine inhibits LKB1 activity, and whereas, aspartate suppresses its activity through direct interaction. Asn asparagine, Asp aspartate.

In addition to changes in AMP or ADP, several metabolic stresses, such as the accumulation of reactive oxygen species (ROS) and the lack of fructose-1,6-bisphosphate (FBP) or ribulose-5-phosphate (Ru-5-P), have been proposed to be involved in AMPK activation by different means^26–28^. In this work, we found an alternative mechanism for the regulation of AMPK and identified a role for LKB1 in sensing cellular asparagine-aspartate homeostasis: LKB1 directly binds to asparagine and aspartate, and its activity is strongly suppressed by asparagine but enhanced by aspartate under certain condition (Fig. 8). Although we found asparagine maintains both lymphoma and human cell survival, the mechanistic study was carried out mainly in human tumour cells due to the limited transfection efficiency of mouse lymphoma tested. Thus, the molecular basis for the regulation and functions of asparagine in lymphoma may need further investigation.

In some types of tumour cells, aspartate can be directed to nucleotide biosynthesis to support proliferation under certain conditions^29–31^. Thus, it appears that both aspartate and asparagine promote proliferation, and their role in tumour growth could be context dependent. In p53 wildtype cells, the disruption of ASNS expression or activity decreases asparagine generation and accumulates aspartate, leading to the activation of LKB1-AMPK signalling and p53-dependent cell cycle arrest. When p53 is absent, the cell cycle continues, and abnormal proliferation and/or apoptosis are triggered. Nevertheless, increased asparagine synthesis in p53-deficient tumour cells implies that, compared to aspartate, asparagine may provide more advantages to these tumour cells. In addition, a long-standing mystery surrounding p53 is why p53-deficient mice predominantly develop lymphomas (~70% of all tumour types)^9,10^. Our findings presented here may provide an important clue to the understanding of this phenomenon. Specifically, the elevation in ASNS expression and asparagine production may directly contribute to lymphomagenesis in p53-null mice.

## Methods

### Antibodies and reagents

The antibodies against the following proteins/epitopes were purchased from the indicated sources: ASNS (Proteintech, 14681-1-AP), p21 (BD Biosciences, 556431), p53 (DO-1)-HRP (Santa Cruz, sc126-HRP), p53 (Pab 1801) (Santa Cruz, sc-98), Phosphorylated-p53(Ser15) (Cell signaling technology, 9284S), MDM2(SMP14) (Santa Cruz, sc965), AMPK (Cell signaling technology, 23A3), phospho-AMPK(Thr 172) (Cell signaling technology, 40H9), Acetyl-CoA Carboxylase (Cell signaling technology, C83B10), Phospho-Acetyl-CoA Carboxylase(Ser79) (Cell signaling technology, 3661), LKB1 (Cell signaling technology, 27D10), Phospho-(Ser/Thr) Phe (Cell signaling technology, 9631), HA(HA-7) (Sigma H3663), FLAG(M2) (Sigma F3165), Actin (Proteintech, 66009-1-LG), Goat anti-rabbit IgG-HRP (Santa Cruz, sc-2004) and Goat anti-mouse IgG-HRP (Santa Cruz, sc-2302), ULK1 antibody [EPR4885(2)] (Abcam, ab128859), Phospho-ULK1 (Ser555) (D1H4) (Cell signaling technology, 5869), Tuberin/TSC2 (Cell signaling technology, 3612), Phospho-Tuberin/TSC2 (Ser1387) (Cell signaling technology, 5584), ATM [2C1 (1A1)] (Abcam, ab78), phospho-ATM (S1981) [EP1890Y] (Abcam, ab81292), Chk1 (Abcam, ab47574), Phospho-Chk1 (Ser345) (Cell signaling technology, 2341), p70 S6 Kinase (49D7) (Cell signaling technology, 2708), Phospho-p70 S6 Kinase (Thr389) (108D2) (Cell signaling technology, 9234). The reagents were purchased from the following sources respectively: Nutlin-3α (Sigma, SML0580),Doxorubicin (Sigma, D1515), Etopside (Sigma, E1383), l-Asparagine monohydrate (Sigma, A8381), l-Aspartic acid (Sigma, A9256), l-Glutamic acid (Sigma, G1251), l-asparagine-(*amide*-^15^N) monohydrate (Sigma, 485896).ANTI-FLAG M2 affinity gel (Sigma, A2220), FLAG peptide (Sigma, F3290), HA peptide (Sino Biological, PP100028-1), Propidium iodide (Sigma, P4170), Anti-HA magnetic beads (Pierce, 88837), Protein A/G agarose (Pierce, 20421), D-Luciferin monosodium salt (Pierce, 88291), l-Asparaginase (ProSpec, ENZ-287), l-Albizziine (Goldbio, A-230-250), 0.4% Trypan blue solution (Amresco, K940). Lipofectamine 2000 (ThermoFisher Scientific, 12566014), RNAiMAX transfection agent (ThermoFisher Scientific, 13778075). Crystal violet (Solarbio, C8470-25).

### Plasmids

The coding sequences corresponding to the full-length human *ASNS*, *p53*, *AMPKα*, *LKB1* and *MBP* were amplified by polymerase chain reaction (PCR) from cDNA library of 293T cells and then cloned into pRK5 empty vector tagged with Flag or HA epitope as indicated. The cloning sequences are as follows: Human *ASNS*: F: 5′-ACGCGTCGACATGTGTGGCATTTGGGC-3′; R: 5′-AACTGCAGCTAAGCTTTGACAGCTGACTTGTAGTGGG-3′. Human *p53*: F: 5′-GCTCTAGAATGGAGGAGCCGCAGTCA-3′; R: 5′ CCGCTCGAGTCAGTCTGAGTCAGGCCC-3′. Human *AMPKα*: F: 5′- GCTCTAGAATGCGCAGACTCAGTTCCTG-3′; R: 5′-CCGCTCGAGTTATTGTGCAAGAATTTTAATTAGA-3′. Human *LKB1*: F: 5′-ACGCGTCGACATGGAGGTGGTGGACCCG-3′; R: 5′-CCCAAGCTTTCACTGCTGCTTGCAGGC-3′. Human *MBP*: F: 5′- ACGCGTCGACATGGCGTCACAGAAGAGACC-3′; R: 5′- CCCAAGCTTTCAGCGTCTAGCCATGGGTG-3′. The full-length luciferase was amplified by PCR from pGL3-Basic vector (Promega, E1751) using the sequences as follows: F: 5′-CCGCTCGAGATGGAAGACGCCAAAAACAT-3′; R: 5′-CGACGCGTTTACACGGCGATCTTTCCG-3′. The PCR product was then cloned into MSCV-IRES-EGFP vector (Addgene plasmid 20672) to construct MSCV-Luciferase-IRES-GFP plasmid. All amplifications were made by PCR and confirmed by DNA sequencing.

### Cell culture and gene knockdown with shRNA and siRNA

All cells were cultured in a 5% CO_2_ humidified incubator (ThermoFisher Scientific, USA) at 37 °C. 293T, HCT116, U2OS, A549, DU145, A431, MEF, L5178Y, EL4 and NCM460 cell lines were routinely maintained in standard dulbecco’s modified eagle’s medium (DMEM) (ThermoFisher Scientific, C11995500BT) with 10% fetal bovine serum (FBS) (ThermoFisher Scientific, 16000044). L1210 cells were cultured in DMEM (ThermoFisher Scientific, C11995500BT) with 10% horse serum (HS) (ThermoFisher Scientific, 16050122). HEK293, HepG2 and WI-38 cells were cultured in minimum essential medium (MEM) (Corning, 10-010-CVR) with 10% FBS (ThermoFisher Scientific, 16000044). Jurkat, U937, MOLT4, K652, H1299 and LNCaP cells were cultured in standard RPMI-1640 medium (ThermoFisher Scientific, 11875093) with 10% FBS (ThermoFisher Scientific, 16000044), unless indicated otherwise. All cells were cultured without the addition of penicillin-streptomycin and examined for mycoplasma contamination and cultured for no more than 2 consecutive months. Their morphologies were confirmed periodically to avoid cross-contamination or misuse of cell lines. None of the cell lines used in this study was listed in the ICLAC database.

shRNA-mediated knockdown of ASNS was performed using a specific targeting sequence shASNS#6 (5′-CCGGGCTCTGTTACAATGGTGAAATCTCGAGATTTCACCATTGTAACAGAGCTTTTTG-3′) or shASNS#10 (5′-CCGGGCTGTATGTTCAGAAGCTAAACTCGAGTTTAGCTTCTGAACATACAGCTTTTTG-3′). A non-specific ‘scrambled’ shRNA sequence (5′-CCGGCAACAAGATGAAGAGCACCAACTCGAGTTGGTGCTCTTCATCTTGTTGTTTTT-3′) was used as control. These sequences were individually cloned into the pLKO.1-puro vector (Sigma, SHC201), which was then co-transfected with the expression vectors containing gag/pol, rev and vsvg genes into 293T cells. The lentivirus was harvested 48 h after transfection and enriched with lentivirus concentration solution (GeneCopoeia, LPR-LCS-01) for 48 h at 4 °C, followed by being added to nearly confluent HCT116 cells with 4 µg/ml polybrene and cultured for 16-24 h to complete infection. The truly stable transfected cells were selected under the pressure of 2 µg/ml puromycin for 1–2 weeks.

siRNA-mediated knockdown was performed with Lipofectamine RNAiMAX transfection agent (ThermoFisher Scientific, 13778075) and siRNAs targeting human *TP53*, *ASNS*, *AMPKα*, *LKB1* and mouse *Asns*, individually or combined for the purpose of experiment. The targeting sequences were as follows: Human *TP53*: 5′-GACTCCAGTGGTAATCTAC-3′, Human *ASNS* 5′-TGTATGTTCAGAAGCTAAA-3′. Human *AMPKα* 5′-ACCAUGAUUGAUGAUGAAGCCUUAA-3′, Human *LKB1* 5′- GGACUGACGUGUAGAACAATT-3′, Human *p21* 5′- CUUCGACUUUGUCACCGAG-3′, human *ATF4* 5′-CAAGCACTTCAAACCTCAT-3′^5^, Human *p73* 5′-GAGCUCGGGAGG GACUUCAACGAAG-3′, Human *p63* 5′-GCACACAAUUGAAACGUACAGGCAA-3′, Human *ATM* 5′-CGTGTCTTAATGAGACTACAA-3′, and Mouse *asns* 5′-GGCUUACUUAGGCAUGAAATT-3′. The siRNA sequence (5′-CGUACGCGGAAUACUUCGATT-3′) targeting luciferase was used as control throughout this study. All siRNAs were used at a concentration of 20 nM. For co-transfection experiments, the total siRNA concentration was equalised under all conditions by control siRNA. The siRNA transfection procedures were performed according to manufacturer’s instructions.

### Semi-quantitative RT-PCR and quantitative RT-PCR

Briefly, total RNA was isolated from triplicate wells in each condition using Total RNA purification Kit (GeneMark, TR01) and 2 µg RNA of each sample was complementarily reversed to cDNA by First-strand cDNA Synthesis System (Thermo scientific, K1621). 0.04 µg cDNA product of each sample was used as template to conduct semi-quantitative or quantitative PCR. The primer pairs used in this study include: Human *ASNS*: F: 5′-GGAAGACAGCCCCGATTTACT-3; R: 5′-AGCACGAACTGTTGTAATGTCA-3′. Mouse *Asns*: F: 5′-GCAGTGTCTGAGTGCGATGAA-3′; R: 5′-TCTTATCGGCTGCATTCCAAAC-3′. Human *MDM2*: F: 5′-ATGGTGAGGAGCAGGC-3′; R: 5′-CTAGATGAGGTAGATGGTC-3′. Human *TP53*: F: 5′-ATGGAGGAGCCGCAGTCAGA-3′; R: 5′-GGCATTCTGGGAGCTTCATC-3′. Human *ACTB*: F: 5′-GACCTGACTGACTACCTCATGAAGAT-3′; R: 5′-GTCACACTTCATGATGGAGTTGAAGG-3′. Mouse *Actb*: F: 5′-ACTACATTCAATTCCATC-3′; R: 5′-CTAGAAGCACTTGCGGTG-3′. Human *p21*: F: 5′- CCGGCGAGGCCGGGATGAG-3′; R: 5′-CTTCCTCTTGGAGAAGATC-3′. Mouse *p21*: F: 5′-AACTTCGTCTGGGAGCGC-3′; R: 5′-TCAGGGTTTTCTCTTGCAGA-3′. Human *ABHD4*: F: 5′-TCACCCACTCTGTCCTTTCC-3′; R: 5′-GTGCAATCCCTTCACATCCT-3′. Human *PML*: F: 5′-CGCCCTGGATAACGTCTTTTT-3′; R: 5′-TCCACAATCTGCCGGTACAC-3′. Human *SIDT2*: F: 5′-GCCAAATTGCTGCTTTCTTC-3′; R: 5′-TCCCTTCCATCCTTCCTCTT-3′. Human H-*RAS*: F: 5′-GACGTGCCTGTTGGACATC-3′; R: 5′-CTTCACCCGTTTGATCTGCTC-3′. Semi-quantitative PCR was performed with Taq PCR StarMix (GeneStar, A112-100) in a thermal cycler (Bio-Rad, T100) according to a standard protocol as follows: 1 cycle at 95 °C for 3 min; 30 cycles at 94 °C for 45 sec, annealing for 45 sec, and 72 °C for 1 min; a final extension at 72 °C for 10 min; and holding at 4 °C. 10 μl PCR products were analysed by electrophoresis through 2% agarose gels with Gel-Red staining (Beyotime, D0139) and visualised by gel imaging analysis system (LIUYI, 130-1310). Quantitative RT-PCR was performed using CFX96 Real-Time PCR System (Bio-Rad, USA) and the amplifications were conducted using the SYBR Green qPCR Master Mix (Biotool, B21202) according to manufacturer′s instructions. The thermal cycling conditions were set as follows: 50 °C for 2 min followed by an initial de-naturation step at 95 °C for 10 min, 45 cycles at 95 °C for 15 s, 60 °C for 1 min, and a dissociation curve at 95 °C for 15 s and 60 °C for 15 s. All experiments were performed in triplicate. The fold changes of gene expression were calculated after being normalised to *ACTB*.

### Western blot analysis

Cells were lysed by using modified RIPA buffer containing 10 mM Tris-HCl at pH 7.5, 5 mM EDTA, 150 mM NaCl, 1% NP-40, 1% sodium deoxycholate, 0.025% SDS and proteinase inhibitors on ice for 10–20 min. Protein samples were quantified using BCA protein assay kit (Macgene, MPK002), boiled in 5 × loading buffer and resolved by SDS-PAGE and transferred onto nitrocellulose membrane. In all, 5% skimmed milk (BD Difco, 232100) in TBS supplemented with 0.1% Tween (TBST) was used to block the membrane before probing with indicated antibodies in TBST at 4 °C overnight. Membranes were washed with TBST and then incubated with HRP-conjugated anti-rabbit or anti-mouse secondary antibodies at room temperature for 1 h and developed with ECL Western Blotting Detection Reagent (ThermoFisher Scientific, 32132). Blot bands were quantified using ImageJ software. Uncropped scans of all the blots in this manuscript are shown in the Supplementary Information.

### Animals

Animal experiments were performed with male C57BL/6J mice (Jackson Laboratory, Jax 664), *p53*^*−/−*^ C57BL/6J mice (BIOCYTOGEN, BCG-DIS-0001) and BALB/c nude mice (Vital River Laboratory Animal Technology, 401). All mice were maintained under specific pathogen-free conditions, and used in accordance with protocols approved by the Institutional Animal Care and Use Committees of Tsinghua University for animal welfare. Mice were initially randomised by age and weight, and were 6–8 weeks of age at the time of injections. The maximum tumour size approved by IACUC protocol was 2 cm in diameter and this was not exceeded in all experiments.

### Establishment of EL4-Luc-GFP EL4 cell line

Retroviruses carrying MSCV-Luciferase-IRES-GFP plasmid were packaged with the Plate-E system. For retroviral infection, EL4 cells were spin-infected with viral solution at 1500 × *g* in the presence of 4 µg per ml polybrene for 2 h at 32 °C. Infected EL4 cells were expanded and GFP positive cells were sorted out by FACSArial II (BD Biosciences, USA) to obtain EL4-Luc-GFP cells. The purity of EL4-Luc-GFP cells was confirmed by a second sorting 1 week after cultural expansion. FACS gating strategies for the identification of EL4-Luc-GFP cells (GFP^+^) were described as in Supplementary Fig. 11a.

### Xenograft tumour models

For xenograft model established through i.v., viable EL4-Luc-GFP cells were washed twice with phosphate-buffered saline (PBS) and resuspended in PBS with the density of 5 × 10^6^ cells/ml, which were subsequently injected into the lateral tail vein in a volume of 0.1 ml. Mice were then treated i.p. with vehicle or ASNase (2 U per g of body weight) every 3 days for 3 weeks. During these days, mice observed with paralysis in hind legs were transferred into new cage and fed with jellylike fodder until the end of the experiment. In total, 3 weeks after xenograft, nearly 100 μl blood samples were collected from the tail vein. In total, 20 μl volume of blood samples were added into PBS with 10% FBS and anticoagulant, after the procedure of red blood cell lysis, the residual white cells were resuspended in PBS with 10% FBS and subjected to FACS analysis using LSR II cytometer (BD Biosciences, USA) to identify GFP positive cells. The rest equal volume of blood samples were centrifuged at 5600 × *g* for 5 min at 4 °C to obtain serum, and store at −80 °C for further analysis. After blood sampling, mice were anaesthetised and injected i.p. with d-Luciferin monosodium salt (6 mg per mouse) and imaged for luciferase activity by IVIS Lumina II multispectral imaging system (Caliper, USA). Mice were further fed to document survive status.

For xenograft model established through s.c., viable EL4-Luc-GFP cells were resuspended in PBS with the density of 1 × 10^7^ cells per ml, which were subsequently injected into the flank of hind legs in a volume of 0.1 ml on both sides. In total, 1 week later, the tumour volume was measured by caliper, then mice were anaesthetised and injected i.p. with d-Luciferin monosodium salt (6 mg per mouse) and imaged for luciferase activity by IVIS Lumina II multispectral imaging system. Next, mice received treatment i.p. with vehicle or ASNase (2 U per g of body weight) every 3 days for 3 weeks. The tumour volume measurement and whole-mouse bioluminescence imaging were conducted every week until the end of the experiment. In total, 3 weeks after xenograft, blood samples were collected and serum were obtained as mentioned before. After blood sampling, mice were killed and tumours were resected and weighted. For another test using s.c. xenograft model to examine the effects of amino acid on tumour growth, viable EL4-Luc-GFP cells were resuspended in PBS with the density of 5 × 10^6^ cells per ml, which were subsequently injected into the flank of hind legs in a volume of 0.1 ml on both sides. One day later, mice were fed with drinking water containing with or without 10 mM Asn or Asp, or intraperitoneally injected with or without 5 or 10 mM Asn or Asp at the end of the experiment. At 10, 14 and 17 days post-xenograft, tumour volume was measured by caliper, and then mice were anaesthetised and imaged in vivo as mentioned before. After imaging, blood serum were obtained and the levels of Asn and Asp were measured by LC-MS as mentioned before.

For xenograft model established in BALB/c nude mice, similarly, viable HCT116 cells transfected with control siRNA or ASNS siRNA were resuspended in PBS with the density of 1 × 10^7^ cells per ml, followed by injection s.c. into the flank of hind legs in a volume of 0.1 ml at single side. In total, 3 weeks after implantation, mice were killed and tumours were resected, weighted and pictured.

### Metabolic flux and LC-MS analysis

HCT116 cells were seeded in 6-cm dishes and cultured overnight. At next day, cells were washed twice with PBS and cultured in medium containing 4 mM ^15^N-aspartate for 48 h. Polar metabolites were then extracted from cells using appropriate volume of 100% acetonitrile. Then, the fully vortexed cell lysates were centrifuged twice at 14,000×*g* for 10 min to purify metabolites, and the soluble fractions were analysed by Multi-reaction monitoring (MRM) mode of UPLC-QQQ-MS/MS (Agilent 1290/6460 tandem mass spectrum, Agilent USA). An ACQUITY UPLC® BEH HILIC, 2.1 mm × 100 mm, 1.7 μm (Waters) was used for LC separation, using gradient elution with 0.1% formic acid acetonitrile as solvent A and 0.1% formic acid water as solvent B. The gradient programme is as follows: 0–1 min 80% A, 1–5 min 80% A to 50% A, 5–7 min 50% A, 7–7.1 min 50% A to 80% A, 7.1–10 min 80% A. The flow rate was set at 0.4 mL per min, and the injection volume was 10 μL. The total run time was 10 min for each sample.

Using a Jet Stream electrospray ESI ion source in positive ion mode was used to detect 5 kinds of amino acids, the nitrogen generator (PEAK Shanghai) was used for solvent removal and atomisation, and high purity nitrogen as colliding gas. Sheath Gas Temp is 350 °C, Sheath Gas Flow flows at 10 L per min, Gas Temp is 325 °C and Gas Flow is 8 L per min. Capillary voltage is 4000 V, Nebulizing Gas is 45 psi, Nozzle Voltage is 500 V.

**Table:** 

Compound	Precurosor ion (m/z)	Product ion (m/z)	Fragmentor	CE
Asn	133	74	60	10
Asp	134	74	60	10

### Chromatin immunoprecipitation

The Genomatix Promoter Inspector software (http://www.genomatix.de) was used to search for potential *TP53* response elements in *ASNS* gene with the consensus sequence 5′-RRRCWWGYYY-(0-13-base pair spacer)-RRRCWWGYYY-3′, where R is a purine, Y a pyrimidine and W either A or T. The sequences for the putative *TP53* response elements in *ASNS* genes are: ASNS-RE1, 5′- ATCATCTTGTGGAGGCAAGTTGACA-3′; ASNS-RE2, 5′-ATGCACTGAAACTGCCATGTCCAGT-3′; ASNS-RE3, 5′-AAGTTCATGTTTAGACTTGGGTCTC-3′.

For ChIP assay, cells were washed with PBS and crosslinked with 1% formaldehyde for 15 min at room temperature. The crosslinking reaction was stopped by the addition of glycine to 125 mM final concentration. Cell lysates were sonicated to generate DNA fragments with the average size below 1000 base pairs and followed by immunoprecipitation with indicated antibodies. Bounded DNA fragments were eluted and amplified by PCR. The used primer pairs were: ASNS-RE1, 5′-CCGCTCGAGATTTCTCAATTTATTTCGG-3′ and 5′-CCCAAGCTTCAAAATACATCAGTGGTC-3′; ASNS-RE2, 5′-CCGCTCGAGACCTGTCTGTAGTTGGTTA-3′ and 5′-CCCAAGCTTACTGTTCTTCCTACTCCAAC-3′; ASNS-RE3, 5′-CCCTTTGCTTTCTGATGGTTCCATGTATGC-3′ and 5′-GATTGAGTATCCCTTATCTGAAATGTTTGGAACCAG-3′; p21-RE, 5′-CCGCTCGAGGTGGCTCTGATTGGCTTTCTG-3′ and 5′-CCCAAGCTTCTGAAAACAGGCAGCCCAAG-3′; ACTB-RE, 5′-CTAGGCGGACTATGAC-3′ and 5′-GACTTGGGAGAGGACT-3′.

### Luciferase reporter assay

Briefly, the DNA fragment containing the potential p53-binding region was amplified by PCR with primers used in ChIP assay and was cloned into a pGL3-promoter vector (Promega). In all, 293T cells were plated 18 h before transfection in 24-well plates and transiently transfected with 500 ng of the reporter plasmid and 500 ng of the p53 expressing plasmid or vehicle using Lipofectamine 2000 reagent (ThermoFisher Scientific, 12566014). The luciferase activity was determined according to the manufacturer’s instructions (Promega). Transfection efficiency was normalised on the basis of the Renilla luciferase activity.

### Soft agar and colony formation assay

HCT116 cells were transfected with control siRNA or *ASNS* siRNA for 24 h and then suspended in 1 ml of DMEM medium supplemented with or without 0.1 mM asparagine plus 20% FBS containing a 0.3% agarose and plated on a firm 0.6% agarose base in 12-well plates (5,00 cells per well). For L1210 and EL4 cells, cells were suspended in 1 ml of DMEM medium supplemented with or without 50 or 150 µM asparagine plus 10% FBS containing a 0.3% agarose and plated on a firm 0.6% agarose base in 12-well plates (800 cells per well). Cells were then cultured in a 5% CO_2_ incubator at 37 °C for 2 weeks. Colonies were fixed with 25% formaldehyde and stained with 0.0125% crystal violet till colonies turned into blue. Colonies were then quantified by counting and images were obtained.

For colony formation assay, 3000 viable cells were seeded in 6-well plate in triplicate with 2 ml culture medium in each well. Every 2 days, the medium were replaced with fresh medium until the end of the experiment. The experiment was ceased when the colonies were clearly visible even without microscopic observation. At last, cellular colonies were fixed with 4% paraformaldehyde solution and stained with 0.05% crystal violet till colonies turned into blue. The ImageJ software was used to analyse the area covered by colonies in each well.

### Analysis of cell-cycle disruptions

Cells were washed twice with PBS and fixed in 75% ethanol overnight at 4 °C. Cells were then incubated in the solution containing 0.1% Triton X-100, 100 µg/ml RNase A and 50 µg/ml propidium iodide for 30 min at 37 °C in the dark. Cell cycle distribution was analysed using a LSR II cytometer (BD Biosciences, USA). The data were analysed using FlowJo software (TreeStar). FACS gating strategies for analysing PI-stained cells in cell cycle distribution analysis were described as in Supplementary Fig. 11b.

### Senescence-associated SA-β-gal activity

The SA-β-gal activity in cultured cells was determined using a Senescence Detection Kit (BioVision, K320-250) according to the manufacturer’s instructions. After staining, cells were imaged and recorded under an inverted microscope (Olympus, IX70) in bright-field. Percentages of cells that stained positive were calculated by counting 1000 cells in random fields per cell line.

### Apoptosis detection

The TUNEL staining was performed using the DeadEnd Fluorometric TUNEL system according to the manufacturer’s instructions (Promega, G3250). After staining, cells were then observed under a confocal laser scanning microscope LSM710 (Zeiss, Germany), and a nucleus displaying bright green fluorescence was recorded as a TUNEL-positive cell.

For annexin V/PI double staining, cells were harvested and washed twice and resuspended in PBS. Apoptotic cells were identified by double-stained with FITC-conjugated annexin-V monoclonal antibody and PI dye for 30 min by using the Annexin V-FITC Apoptosis Detection kit (BD Biosciences, 556547). After staining and washing, cells were analysed by LSR II cytometer. Apoptotic cells were defined as annexin-V^+^PI^−^ cells. FACS gating strategies were described as in Supplementary Fig. 11c.

### Cell proliferation and viability

For leukaemia cell lines, 1 × 10^5^ cells were plated in 12-well plate with 1.5 ml culture medium as indicated and supplemented with or without compound treatment. In total, 2 days later, cells were washed with PBS and stained with 0.4% trypan blue for 2 min and counted by TC20 Automated Cell Counter (Bio-Rad, USA). Viable cells and dead cells were recorded. In co-culture assay, 1 × 10^6^ HCT116 cells were plated in 6-well plate with 2 ml culture medium, 12 hours later, culture medium were replaced with fresh medium containing 1 × 10^5^ leukaemia cells, and further co-cultured for 2–3 days. The viable cells and dead cells were determined as mentioned above. For adherent cell lines, 2 × 10^6^ cells were plated in 6-well plate with 2 ml culture medium with or without treatment as indicated, Culture medium were replaced with fresh medium every 2 days, if needed. At the end of experiment, the viable cells and dead cells were determined.

### In vitro measurement of ASNS enzyme activity

Cells were collected in 1.5 ml tubes and 1/15 of them were prepared for protein quantification by BCA assay, and the rest were washed three times with PBS and placed on ice. Each sample was resuspended in 400 μl sample buffer (50 mM Tris HCl, pH8.0, 0.5 mM EDTA, 1 mM EGTA, 1 mM DTT and 1 mM PMSF), frozen in liquid nitrogen and dissolved in room temperature for 4 times. Cell extracts were centrifuged at 12,000 × *g* for 30 min at 4 °C, and the supernatants were applied to analyse the enzymatic activity of ASNS by reacting with a substrate mixture (85 mM Tris HCl, pH 8.0, 50 mM NaCl, 8.33 mM MgC1_2_, 5 mM ATP, 10 mM aspartic acid and 5 mM glutamine). In each reaction, 40 μl of enzyme solution were added to 20 μl substrate mixture and incubated with slight oscillation at 37 °C for 60 min. Each reaction was done in triplicate. The reaction system was placed on ice and each was mixed with three times the volume of acetonitrile, followed by centrifuging twice at 14,000 × *g* for 10 min to extract metabolites. Asn and Glu were identified and quantified by a Triple Quadrupole LC/MS System (Agilent, 1290/6460) according to calibration curve. ASNS activity was obtained by calculating the production of Asn by cell extracts with determined amount of protein in certain period. And the unit for ASNS activity is μg of Asn per minute per μg total protein (μg min^−1^ μg per protein).

### LC-MS analysis of metabolites

For fluid samples, metabolites in mouse serum or 200 μl supernatants collected from culture medium were extracted by 100% acetonitrile by the ratio of 1:3. For cell samples, adhesive cells were harvested and washed twice with PBS, the viable number of cells was recorded prior to metabolite extraction. The minimum 1 × 10^5^ cells were required for LC/MS analysis. Metabolites in cells were extracted by appropriate volume of 100% acetonitrile. For tissue samples, ultrasonication was utilised to obtain tissue extractions, which were centrifuged at 14,000 × *g* for 20 min at 4 °C. The supernatants were collected and quantified for protein level by BCA assay. Metabolites in these supernatants were extracted by 100% acetonitrile by the ratio of 1:3. Then, the mixtures were centrifuged twice at 14,000 × *g* for 10 min to extract metabolites, and the soluble fractions were analysed at original concentration or diluted in 80% acetonitrile by a Triple Quadrupole LC/MS System (Agilent, 1290/6460).

### Protein expression and purification

pRK5 plasmids tagged with Flag or HA epitope coding the full-length human AMPKα, LBK1 and MBP were transfected into HEK-293T cells respectively. In total, 36 h after transfection, cells were harvested and lysed by sonification in lysis buffer (50 mM Tris HCl, pH 7.4, with 150 mM NaCl, 1 mM EDTA, and 1% Triton X-100), and then centrifuged at 13,000 × *g* for 10 min at 4 °C. FLAG M2 or HA beads were used to immunoprecipitate tagged proteins in supernatants according to the manufacturer’s standard procedures. Beads were washed 3–5 times with wash buffer (20 mM Tris, pH 7.5, 150 mM NaCl, 1% TritonX-100) at 4 °C on a rotator, followed by competitive elution with appropriate synthetic FLAG or HA Peptide in TBS or PBS solution depending on experimental purpose. Purified proteins were used immediately or stored at −80 °C.

### Measurement of LKB1 kinase activity

Active Flag-tagged LKB1 was immunoprecipitated from cell extracts of 293T cells using the FLAG M2 beads. Beads were washed three times in lysis buffer and twice in kinase buffer (50 mM Tris, pH 7.5, 10 mM MgCl_2_, 1 mM DTT, 100 µM ATP), and then resuspended in kinase buffer containing increasing concentrations of Asn or Asp for nearly 1 hour at 4 °C on a rotator. HA-MBP or HA-AMPKα bound on HA beads were equally allocated into each reaction in 0.2 ml 8-Strip Tubes. These HA beads were resuspended by kinase buffer containing Flag-tagged LKB1 and kinase reaction was performed for 30 min at 30 °C on a rotator. Samples were subjected to 8% SDS-PAGE, followed by Western blot.

### In vitro pull-down assay

For in vitro AMPKα and LKB1 interaction, Flag-tagged LKB1 and HA-tagged AMPKα proteins were expressed in 293 T cells. Cells were lysed and proteins were immunoprecipitated using the FLAG M2 or HA beads according to the manufacturer’s standard procedures. The Flag-tagged LKB1 proteins were purified and dissolved in the binding buffer (20 mM Tris pH 7.5, 150 mM NaCl) with or without different concentrations of Asn or Asp for nearly 1 h at 4 °C on a rotator. The HA beads bound with HA-tagged AMPKα were equally allocated into 0.2 ml 8-Strip Tubes and washed with the wash buffer. These HA beads were then resuspended with binding buffer containing Flag-tagged LKB1 proteins and incubated for 4 h at room temperature, washed by the wash buffer for five times on a rotator, and subjected to 8% SDS-PAGE, followed by Western blot.

### Surface plasmon resonance analysis

SPR analysis was conducted at 25 °C with a Biacore T200 instrument (GE, USA) according to the manufacturer’s instructions. Purified LKB1 was immobilised on the surface of a Series S Sensor chip CM7 (GE, 28-9538-28) in 10 mM sodium acetate buffer (pH 4.5) and resulted in nearly 8000 response units. A reference surface was used as a blank to correct for instrumental and buffer effects without protein injection. The amount of protein bound to the sensor chip was monitored by the change in refractive index. Asn or Asp was dissolved in PBS and diluted with gradient concentrations and run across each sensor surface at eight different concentrations in a running buffer of PBS at a flow rate of 30 µl per min for 90 s (contact phase), followed by 120 s of buffer flow (dissociation phase). Dissociation constants from eight serial dilutions of each compound and other kinetic parameters were calculated using the Biacore T200 Evaluation software Version 1.0.

### CRISP/Cas9-mediated knockout of LKB1

To generate a *LKB1*-knockout HCT116 cell line, a lentiviral CRISPR/Cas9 plasmid targeting *LKB1* was constructed by cloning the annealed sgRNA into pLenti-CRISPRv2 vector as previously described^19^. The sgRNAs were designed by CRISPER Design tool (crispr.mit.cn), and the sequences are: 5′-CACCGAGCTTGGCCCGCTTGCGGCG-3′ and 5′-AAACCGCCGCAAGCGGGCCAAGCTC-3′. In all, 293T cells were cotranfected with pLenti-CRISPRv2, pVSVg and psPAX2 to produce lentiviruses, which were used to infect HCT116 cells for 24 h, and then *LKB1*-knockout cell line were obtained by culturing in medium containing 2 μg/ml puromycin for a week.

### Radiolabeled amino acid binding assay

Around five million 293T cells were seeded in a 10-cm plate, and the next day, cells were transfected with 10 µg pRK5-FLAG-based cDNA expression plasmids individually as indicated in the experiment via Lipofectamine 2000. Forty-eight hours after transfection, cells were rinsed one time with ice-cold PBS and immediately lysed for 20 min with Triton lysis buffer (1% Triton, 10 mM β-glycerol phosphate, 10 mM pyrophosphate, 40 mM Hepes pH 7.4, 2.5 mM MgCl_2_ and proteinase inhibitors). The cell lysates were cleared by centrifugation at 16,200 × *g* at 4 °C in a microcentrifuge for 10 min. For anti-FLAG-immunoprecipitation, the FLAG-M2 affinity beads were blocked by rotating in 1 µg per µl bovine serum albumin (BSA) for 1 h at 4 °C and subsequently washed for three times with lysis buffer. In total, 30 µl of a 50/50 slurry of the affinity beads were then added to clarified cell lysates and incubated with rotation for overnight at 4 °C. Following immunoprecipitation, the beads were washed one time with lysis buffer and three times with lysis buffer containing 500 mM NaCl. If multiple samples of the same type were represented in the experiment, the beads were equally distributed amongst the relevant tubes to ensure equal protein amounts across samples of the same type. For the binding assays, the beads was incubated for 1 h on ice in cytosolic buffer (0.1% Triton, 40 mM HEPES pH 7.4, 10 mM NaCl, 150 mM KCl, 2.5 mM MgCl_2_) with 3 µCi [^3^H]-labelled amino acids with or without 10 mM corresponding cold amino acids. During incubation, tubes were flicked every five minutes. At the end of one hour, the beads were briefly spun down, aspirated dry, and rapidly washed three times with binding wash buffer (0.1% Triton, 40 mM HEPES pH 7.4, 150 mM NaCl). The beads were aspirated dry again and resuspended in 50 µl of binding wash buffer. With a cut tip, each sample was mixed well and three 10 µl aliquots were separately added into each well of Isoplate-96 and quantified using a MicroBETA2 scintillation counter (PerkinElmer). This process was repeated in pairs for each sample, to ensure similar incubation and wash times for all samples analysed across different experiments. After reading, beads were denatured by the addition of 50 µl of sample buffer and boiling for 5 min, resolved by 10% SDS-PAGE, and analysed by Coomassie blue staining to detect the immunoprecipitated proteins.

### Statistical analysis

All the data are presented as mean ± standard deviation of the mean (s.d.) unless indicated otherwise. An unpaired two-tailed Student’s *t*-test was used to calculate the *P* values, except specified otherwise. *P* < 0.05 was considered significant. *p < 0.05, **p < 0.01, ***p < 0.001, NS not significant.

### Reporting summary

Further information on research design is available in the Nature Research Reporting Summary linked to this article.

## Supplementary information

Supplementary Information

Reporting Summary

## Data Availability

The source data underlying Figs. 1b, c, e–i, 2b, f, h–p, 3a, c, d, f–k, 4a–d, h–j, 5i, 6a–e and 7i, g, h, and Supplementary Figs. 1b–e, g, h, 2a–g, i–l, 3i, l, 4a–c, 5a, c–f, i, j, 6a, b, e, 7e–g, 8b–e, 9c, f, i–k and 10i, j are provided as a Source Data file. All other data are within the paper and its Supplementary Information files.

## Source data

Source Data
