# Supplementary material for: p53-mediated control of aspartate-asparagine homeostasis dictates LKB1 activity and modulates cell survival
Source: Nat Commun. 2020 Apr 9;11:1755. doi: 10.1038/s41467-020-15573-6 (PMC7145870; doi:10.1038/s41467-020-15573-6)
Supplement: Supplementary file 1 — Supplementary Information [file 41467_2020_15573_MOESM1_ESM.pdf]

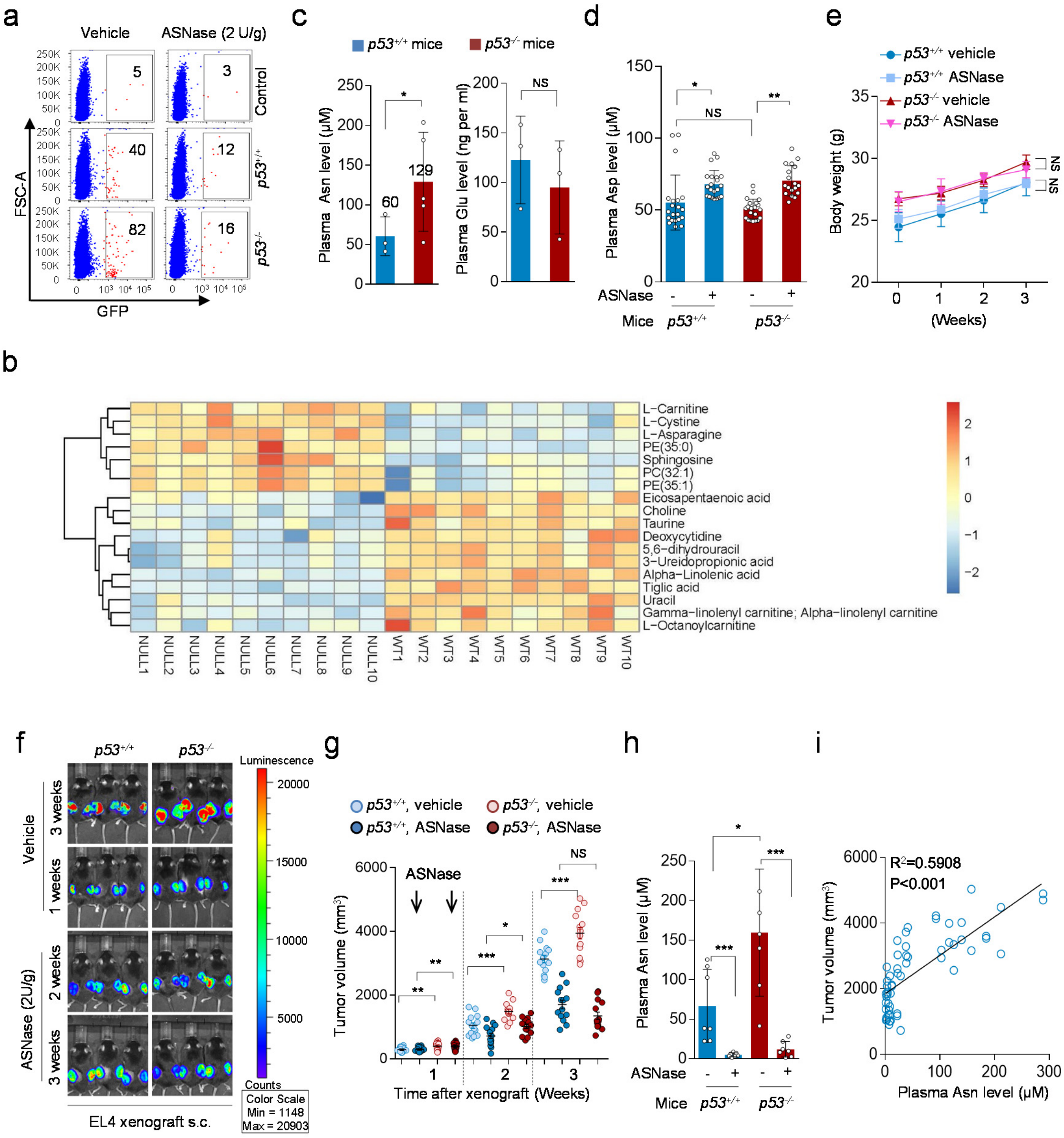

Supplementary Figure 1| p53 deficiency increases plasma asparagine and promotes lymphoma cell proliferation in vivo. a, Related to **Fig. 1a, b.** Flow cytometry analysis of the frequencies of EL4-Luc-GFP cells (GFP^+) in peripheral blood in recipient mice 3 weeks post-injection as depicted in Fig. 1**a**. Peripheral blood of normal mouse without injection or treatment was used as control. Representative flow cytometry plots is shown. The value indicates the absolute number of GFP^+ cells per 10^5 white cells. Each group includes 9 mice. **b**, Heatmap of metabolites at significantly different levels in serum from p53-null (NULL) group and p53-wildtype (WT) group. Each replicate sample is plotted. n=9 mice for each group. **c**, Plasma asparagine (Asn) and glutamate (Glu) concentrations in *p53*$^{+/+}$ and *p53*$^{-/-}$ C57BL/6J mice were measured by LC-MS. Each group contains 5 mice. The mean value of Asn was shown. **d**, Plasma aspartate (Asp) concentrations in *p53*$^{+/+}$ and *p53*$^{-/-}$ C57BL/6J mice treated as in **Figure**

1a measured by LC-MS after 3 weeks of treatment. n=9 mice for each group. **e**, Body weight of *p53*$^{+/+}$ and *p53*$^{-/-}$ C57BL/6J mice treated as in Figure 1a. Each group includes 9 mice. **f**, *p53*$^{+/+}$ and *p53*$^{-/-}$ C57BL/6J mice were xenografted s.c. with 1×10^6 EL4-Luc-GFP cells in the flank of hind legs. One week later, mice were treated i.p. with vehicle or ASNase (2 U per g of body weight) as indicated every 3 days for 3 weeks. The whole-mouse bioluminescence was performed at indicated time points post-injection. Representative images of the mice are shown and the color scale represents the intensity of emitted luminescence. n=7 mice for each group. **g**, Volumes of xenografted from (**f**) were measured by caliper at 1, 2 and 3 weeks post-xenograft and calculated with the formula W $\times$ $L^2/2$. Each group includes 7 mice and 14 tumors. **h**, Plasma Asn concentrations in *p53*$^{+/+}$ and *p53*$^{-/-}$ C57BL/6J mice treated as in (**f**) were measured by LC-MS after 3 weeks of xenografting. Each group includes 7 mice. **i**, Linear regression analysis of mouse plasma Asn concentrations and volumes of xenografted tumors. Data from total 28 mice and 56 tumors were pooled and analyzed. The values of R^2 and P are shown. Data are mean ± s.d., unpaired two-tailed Student's t-test, *p<0.05, **p<0.01, ***p <0.001, NS, not significant. Source data are provided as a Source Data file.

Supplementary Fig. 2

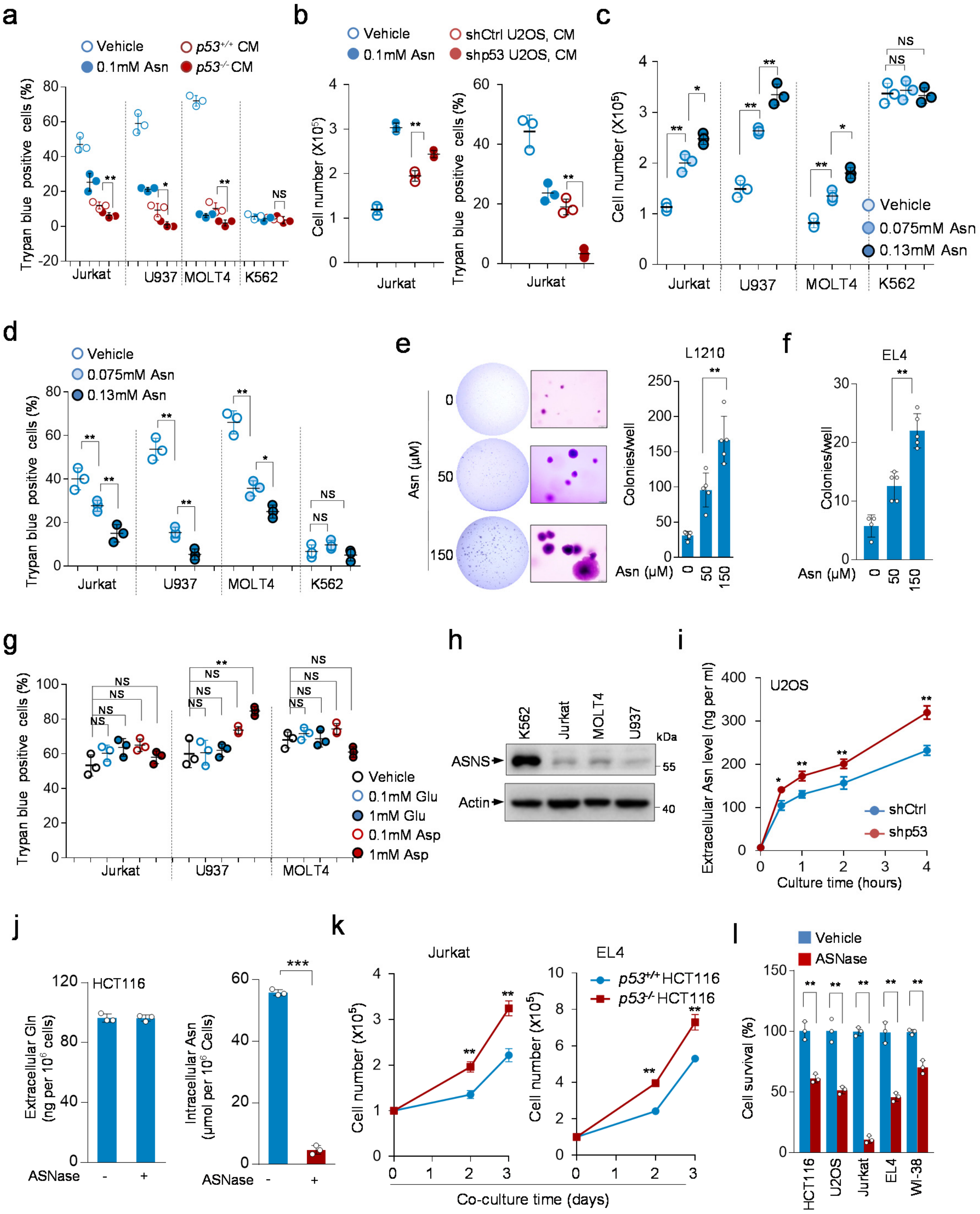

Supplementary Figure 2 | Asparagine is critical for tumor cell survival and proliferation. a, Jurkat, U937, MOLT4 and K562 cell lines were cultured for 48 hours in DMEM medium containing no asparagine (Asn) or 0.1mM asparagine, or cultured in *p53*$^{+/+}$ or *p53*$^{-/-}$ HCT116 cell conditioned-medium(CM) as indicated. Relative cell death (% of trypan blue positive cells) were calculated. Data are mean ± s.d. (n=3). **b**, Proliferation and relative cell death (% of trypan blue positive cells) of Jurkat cells cultured in DMEM media with or without 0.1mM Asn, or cultured in conditioned

medium (CM) from U2OS cells expressing control shRNA (shCtrl) or p53 shRNA (shp53 as indicated. Data are mean ± s.d. (n=3). **c** and **d**, Proliferation (**c**) and relative cell death (% of trypan blue positive cells, **d**) of Jurkat, U937, MOLT4 and K562 cell cultured in DMEM medium containing no asparagine (Asn), 0.075 mM or 0.13 mM asparagine for 48 hours. Data are presented as mean ± s.d. of triplicates and representative of three biological replicates (n=3). **e** and **f**, L1210 (**e**) and EL4 (**f**) cells were plated (1000 cells per well) in soft agar containing 0, 50 or 150 μM Asn and cultured for 2 weeks. Colonies with a diameter greater than 20 μm were quantified. Data are mean ± s.d. (n=3). **g**, Relative trypan blue positive cells (%) of Jurkat, U937 and MOLT4 cell lines cultured in DMEM containing 0, 0.1 or 1 mM Glutamate (Glu) or Asp for 48 hours. Data are mean ± s.d. (n=3). **h**, Western blot analysis of ASNS expression in human leukemia cell lines. β-Actin was used as loading control. **i**, Asparagine (Asn) concentrations in culture medium from shCtrl or shp53 U2OS cells at indicated culture time points were measured by LC-MS. Data are mean ± s.d. (n=3). **j**, HCT116 cells were treated with or without ASNase for 48 hours. Medium glutamine (Gln) and cellular asparagine (Asn) were determined by LC-MS. Data are mean ± s.d. (n=3). **k**, Proliferation of Jurkat (left) and EL4 (right) cells co-cultured with $p53^{+/+}$ or $p53^{-/-}$ HCT116 cells for indicated time points. Data are mean ± s.d. (n=3). **l**, Cells were treated with vehicle or 2 U per ml ASNase for 48 hours, and percentages of cell survival were determined by trypan blue exclusion assay. Data are mean ± s.d. (n=3). *p<0.05, **p<0.01, ***p <0.001, NS, not significant (Student's t-test). Source data are provided as a Source Data file.

Supplementary Fig. 3

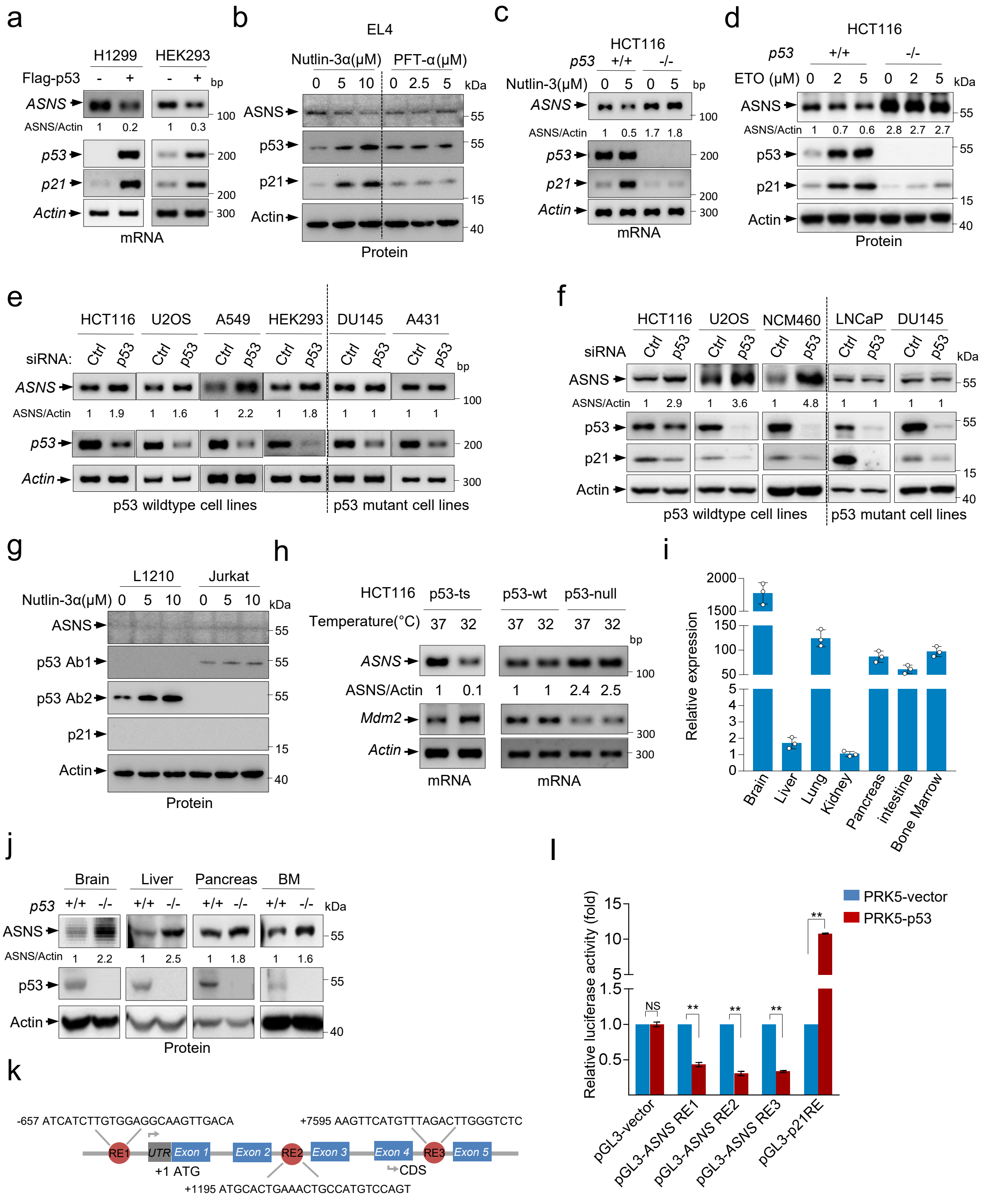

Supplementary Figure 3 | Identification of ASNS is a target for p53. a, mRNA levels of ASNS in H1299 and HEK293 cells transfected with Flag vector control or Flag-p53 as indicated for 48 hours. Relative ASNS/Actin ratios are shown. **b**, protein expression in EL4 cells treated with increasing amounts of Nutlin-3α or PFT-α was analyzed by western blot. **c**, mRNA expression of *ASNS* in *p53*$^{+/+}$ and *p53*$^{-/-}$ HCT116 cells treated with 0 or 5 μM Nutlin-3 for 36 hours. **d**, Protein expression of ASNS in *p53*$^{+/+}$ and *p53*$^{-/-}$ HCT116 cells treated with 0, 2, or 5 μM Etopside (ETO) for 36 hours. Relative

ASNS/Actin ratios are shown. **e**, Semi quantitative RT-PCR analysis of ASNS expression in cell lines with wild-type *p53* (HCT116, U2OS, A549 and HEK293) or mutant *p53* (DU145 and A431) transfected with control siRNA targeting luciferase (Ctrl) or p53 siRNA for 48 hours. Relative ASNS/Actin ratios are shown blow. **f**, Wild-type p53 cell lines (HCT116, U2OS, NCM460) and cell lines harboring mutant *p53* gene (LNCaP and DU145) were transfected with control siRNA targeting luciferase (Ctrl) or p53 siRNA for 48 hours. ASNS expression was determined by western blot. Relative ASNS/Actin ratios are shown. **g**, protein expression in L1210 and Jurkat cells treated with increasing amounts of Nutlin-3α for 36 hours was analyzed by western blot using indicated antibodies. **h**, mRNA levels of ASNS in $p53^{+/+}$ HCT116 cells or $p53^{-/-}$ HCT116 cells expressing vector control, or $p53^{-/-}$ HCT116 cells expressing p53A138V mutant cultured at 32 °C or 37 °C for 24 hours. Relative ASNS/Actin ratios are given. **i**, ASNS mRNA levels in brain, liver, lung, pancreas, kidney, intestine and bone marrow (BM) from C57BL/6J mice. Data are relative to that in pancreas tissue (% of pancreatic expression of ASNS), mean ± s.d. (n=5). **j**, Western blot analysis of ASNS expression in brain, liver, pancreas and bone marrow (BM) tissues from $p53^{+/+}$ and $p53^{-/-}$ C57BL/6J mice. n=5 mice for each group. Relative ASNS/Actin ratios are given. **k**, The schematic exon/intron organization and three putative p53 response elements (RE1, RE2 and RE3) within the human *ASNS* gene. **l**, Luciferase reporter constructs containing the p53 response element (*ASNS*-RE1, RE2, RE3 and *p21*-RE constructs) were transfected into 293T cells together with Flag-vector or Flag-p53 plasmids. Renilla vector pRL-CMV was used as a transfection internal control. Relative levels of luciferase are shown. Data are mean ± s.d. (n=3). *p<0.05, **p<0.01, ***p <0.001, NS, not significant (Student's t-test). Source data are provided as a Source Data file.

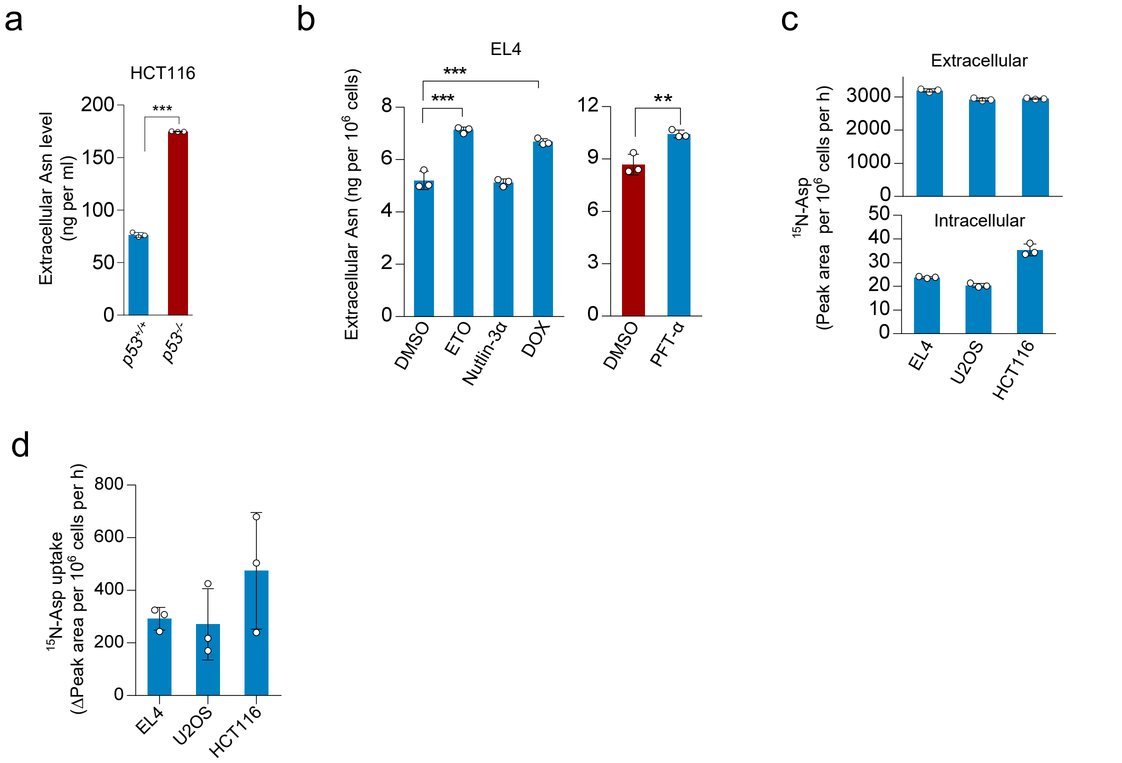

Supplementary Figure 4 | effect of p53 on asparagine production. a, Asparagine levels in medium cultured from *p53*$^{+/+}$ and *p53*$^{-/-}$ HCT116 cells. Data are mean ± s.d. (n=3). **b**, Asparagine levels in medium from EL4 cells treated with DMSO, etoposide(ETO), Nutlin-3α or PFT-α as indicated for 24 hours. Data are mean ± s.d. (n=3). **c**, Extracellular (medium) and intracellular amounts of ^{15}N-Asp in EL4, U2OS and HCT116 cells cultured in medium containing ^{15}N-Asp for 24 hours. **d**, EL4, U2OS and HCT116 cells were cultured in medium containing ^{15}N-Asp for 24 hours. The uptake of ^{15}N-Asp was measured by LC-MS. *p<0.05, **p<0.01, ***p <0.001, NS, not significant (Student's t-test). Source data are provided as a Source Data file.

Supplementary Fig. 5

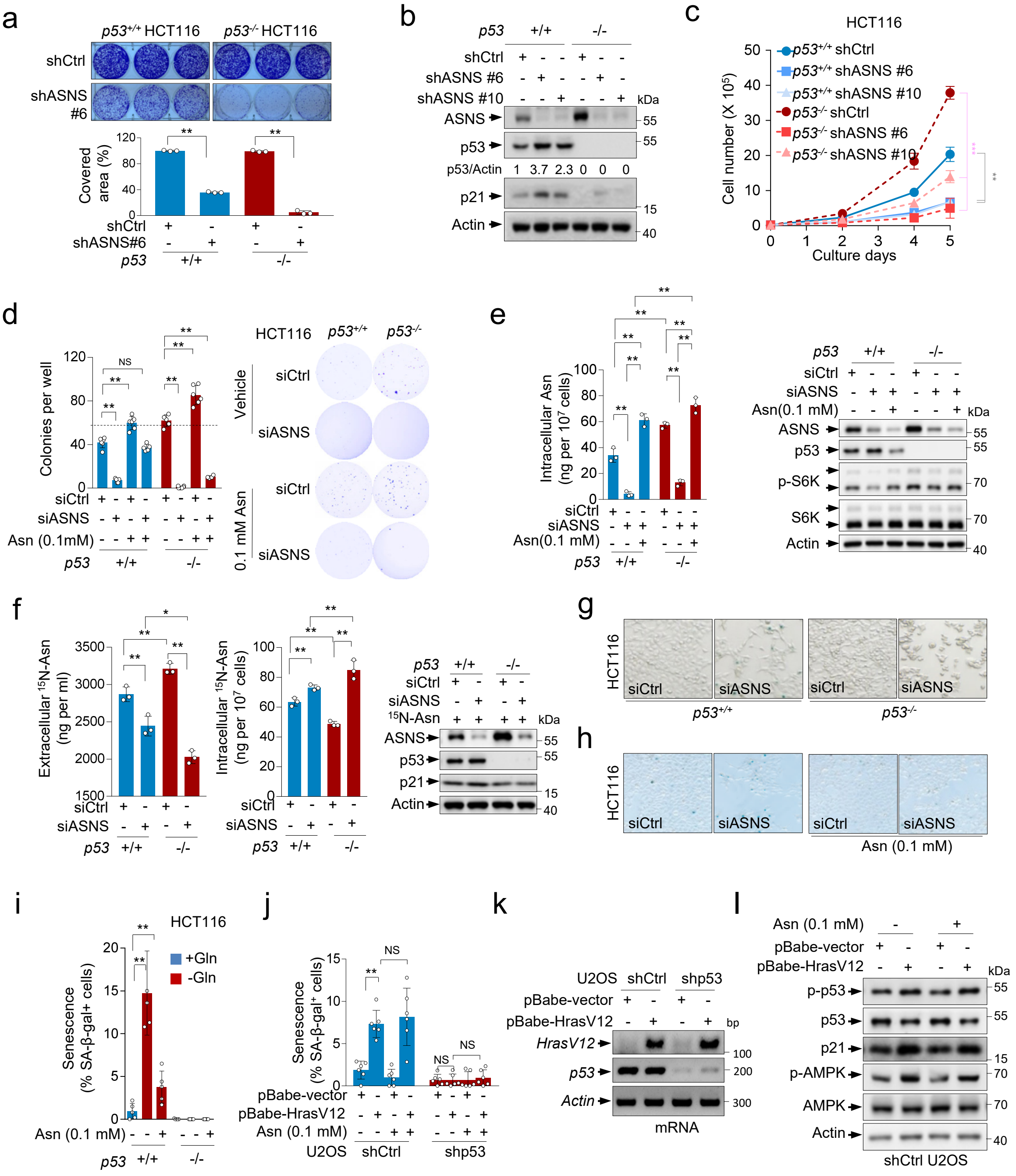

Supplementary Figure 5 | Depletion of *ASNS* inhibits tumor cell proliferation and accelerates senescence through asparagine. a, Proliferation of *p53*$^{+/+}$ and *p53*$^{-/-}$ HCT116 cells stably expressing shCtrl or shASNS #6. Representative images of cells stained with crystal violet at day 12 (up panel). The covered area was quantified (bottom panel). Data are mean ± s.d. (n=3). **b**, Lysates from *p53*$^{+/+}$ and *p53*$^{-/-}$ HCT116 cells stably expressing shCtrl or different sets of *ASNS* shRNAs (shASNS #6 and shASNS #10) were analyzed by western blot. The expressions of ASNS, p53 and p21 are shown, and relative p53/Actin ratios are given. **c**, Proliferation of *p53*$^{+/+}$ and *p53*$^{-/-}$ HCT116 cells stably expressing shCtrl, shASNS #6 or shASNS #10. Cell number was counted

at indicated time points. Data are mean ± s.d. (n=3). **d**, *p53*$^{+/+}$ and *p53*$^{-/-}$ HCT116 cells transfected with control siRNA (siCtrl) or ASNS siRNA (siASNS) were plated (1000 cells/well) in soft agar with or without of 0.1 mM Asn. Cells were cultured for 2 weeks and colonies with a diameter greater than 20 µm were quantified (left panel). Data are mean ± s.d. (n=3). Representative images of colonies stained with crystal violet at day 14 (right panel). **e**, *p53*$^{+/+}$ and *p53*$^{-/-}$ HCT116 cells transfected with control siRNA (siCtrl) or ASNS siRNA (siASNS) were cultured in medium containing 0.1 mM Asn for 3 days. Intracellular Asn levels were determined by LC-MS analysis. Expressions of ASNS, p53, p-S6K and S6K are shown. Data are mean ± s.d. (n=3). **f**, *p53*$^{+/+}$ and *p53*$^{-/-}$ HCT116 cells transfected with control siRNA (siCtrl) or *ASNS* siRNA (siASNS) were cultured in medium containing 0.1 mM ^{15}N-Asn for 3 days. Both extracellular (left panel) and intracellular (middle panel) Asn levels were determined by LC-MS analysis. Protein expression is shown (right panel). Data are mean ± s.d. (n=3). **g**, Representative images of β-galactosidase (SA-β-gal) staining of *p53*$^{+/+}$ and *p53*$^{-/-}$ HCT116 cells transfected with siCtrl or siASNS for 5 days. **h**, Representative images of SA-β-gal staining of HCT116 cells transfected with siCtrl or siASNS for 5 days in the presence or absence of 0.1 mM Asn. **i**, *p53*$^{+/+}$ and *p53*$^{-/-}$ HCT116 cells cultured in medium containing no or 3 mM glutamine (Gln) for 48 hours in the presence or absence of 0.1 mM Asn. Cell senescence (% of SA-β-gal positive cells) were determined. Data are mean ± s.d. (n=3). **j** and **k**, U2OS cells stably expressing control shRNA (shCtrl) or p53 shRNA, and/or HrasV12 as indicated were cultured in medium with or without 0.1 mM asparagine (Asn) for 5 days. Percentages of senescence-associated β-galactosidase (SA-β-gal)-positive cells (**j**). Expressions of p53 and HrasV12 were analyzed by PCR (**k**). Data are mean ± s.d. (n=3). **l**, shCtrl U2OS cells expressing HrasV12 or vector control were cultured in medium containing no asparagine or 0.1 mM asparagine for 5 days. Expressions of p53, AMPK, p53 phosphorylation and p21 were determined by western blot analysis. *p<0.05, **p<0.01, ***p <0.001, NS, not significant (Student's t-test). Source data are provided as a Source Data file.

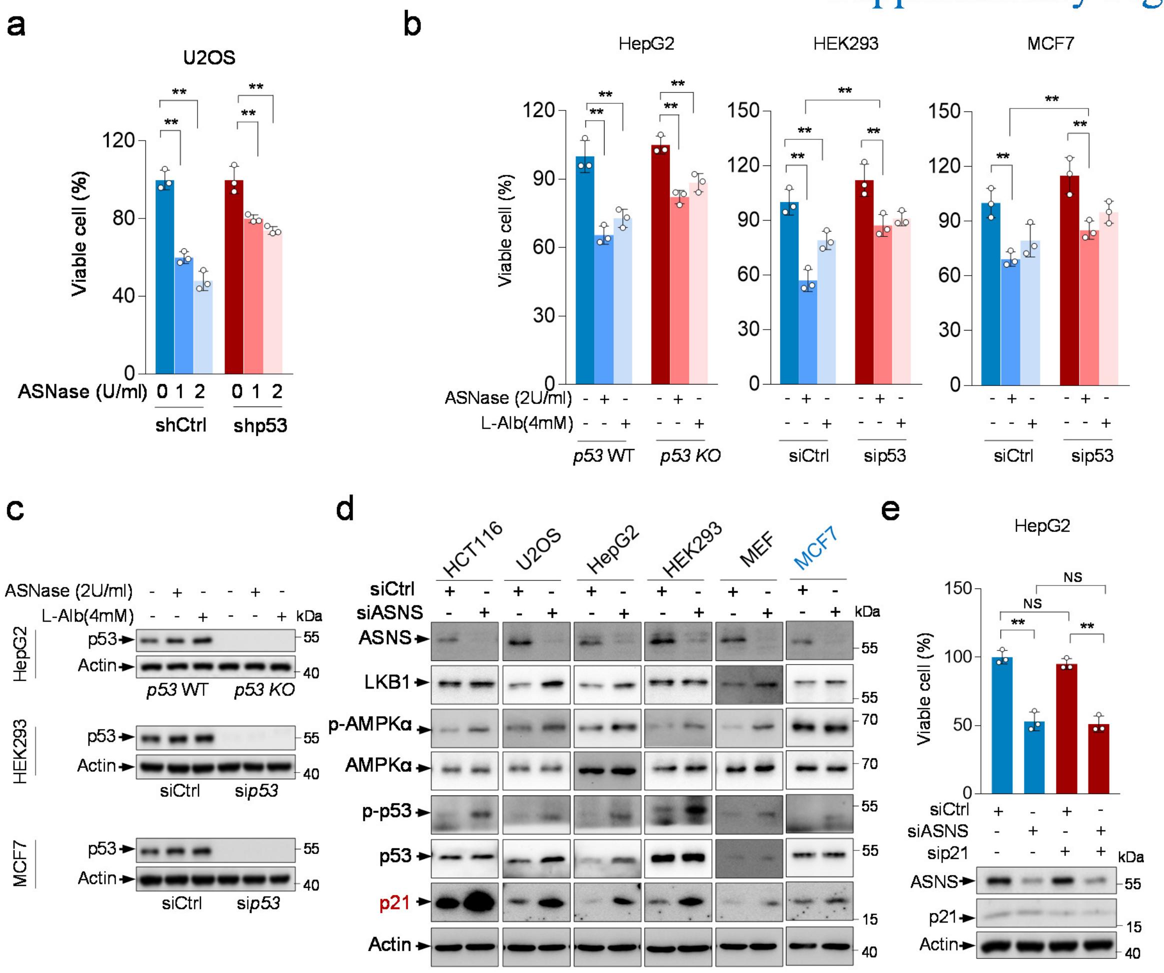

Supplementary Figure 6 | Depletion of asparagine reduces cell survival. a, U2OS cells stably expressing shCtrl or shp53 were treated with increasing amounts of ASNase for 2 days. Cells were stained with trypan blue and percentages of viable cells were calculated. Data are mean ± s.d. (n=3). **p<0.01. **b** and **c**, p53-wildtype (WT) and p53-knockout (KO) HepG2 cells, siCtrl and sip53 HEK293 cells, or siCtrl and sip53 MCF7 cells were cultured in medium containing 2U per ml ASNase or 4mM L-Alb as indicated for 2 days. Percentages of viable cells were determined by trypan blue exclusion assay (**b**). Protein expressions of p53 and Actin were analyzed by western blotting (**c**). Data are mean ± s.d. (n=3). **d**, Western blot analysis of equal lysates from multiple cell lines transfected with control siRNA (siCtrl) or ASNS siRNA (siASNS) for 2 days. **e**, HepG2 cells were transfected with control siRNA, ASNS siRNA and/or p21 siRNA as indicated. Percentages of viable cells were calculated and protein expressions are assayed by western blotting. Data are mean ± s.d. (n=3). *p<0.05, **p<0.01, ***p <0.001, NS, not significant (Student's t-test). Source data are provided as a Source Data file.

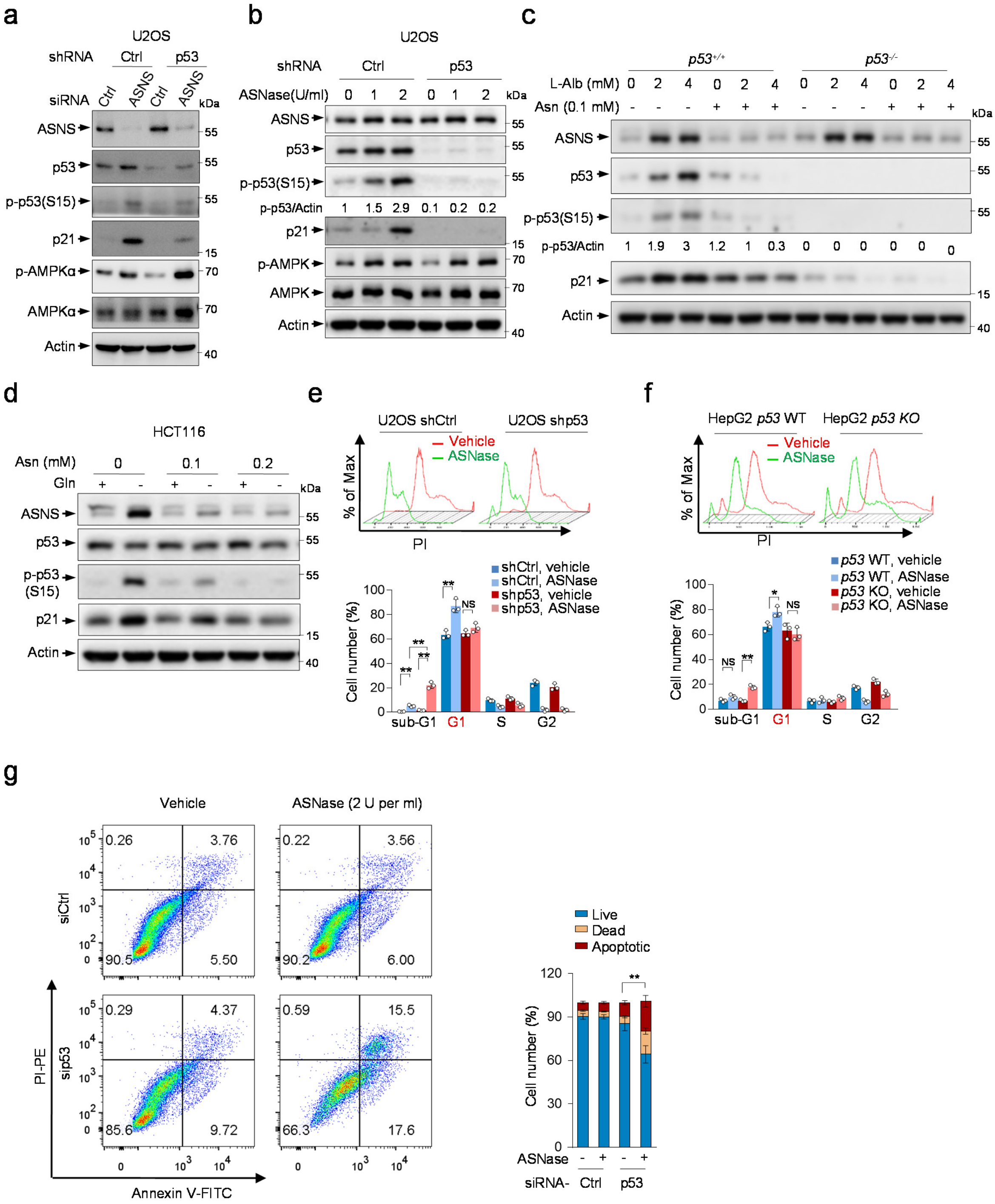

Supplementary Figure 7 | Asparagine depletion triggers p53 activation, p53-dependent cell cycle arrest and induces apoptosis in *p53*-depeleted cells. a, U2OS cells stably expressing control shRNA or p53 shRNA were transfected with control siRNA (siCtrl) or ASNS siRNA (siASNS) as indicated for 3 days. Protein expression was analyzed by western blot. Relative p-p53/Actin ratios are shown blow. **b**, U2OS cells stably expressing control shRNA or p53 shRNA were treated with increasing amounts of ASNase for 2 days. Protein expression was analyzed by western blot. **c**,

Western blot analysis of *p53*$^{+/+}$ and *p53*$^{-/-}$ HCT116 cells treated with increasing amounts of L-Albizziine (L-Alb) (0, 2 or 4 mM) in the presence or absence of 0.1 mM asparagine (Asn) for 48 hours. Relative p53/Actin ratios are shown. **d**, Protein levels of ASNS, p53, p21 and phosphorylated p53 (S15) in HCT116 cells cultured in complete medium or glutamine (Gln)-free medium for 48 hours in the presence or absence of 0.1 or 0.2 mM asparagine (Asn) as indicated. **e** and **f**, Cell cycle distribution of shCtrl and shp53 U2OS cells (**e**), or p53-wildtype and p53-knockout HepG2 cells (**f**) in the presence or absence of 2 U per ml ASNase for 48 hours. Percentages of cells in the sub-G1, G1, S, and G2 phases were determined by PI staining and flow cytometry analysis. Data are mean ± s.d. (n=3). **g**, HCT116 cells transfected with control siRNA (siCtrl) or p53 siRNA (sip53) were cultured in medium with or without 2 U per ml ASNase for 48 hours. Apoptosis was analyzed by Flow cytometry analysis with annexin V/PI double staining. Representative flow cytometry plots (left) and statistically quantitative results of live, dead and apoptotic cells (right) are shown. Data are mean ± s.d. (n=3). *$p<0.05$, **$p<0.01$, ***$p <0.001$, NS, not significant (Student's t-test). Source data are provided as a Source Data file.

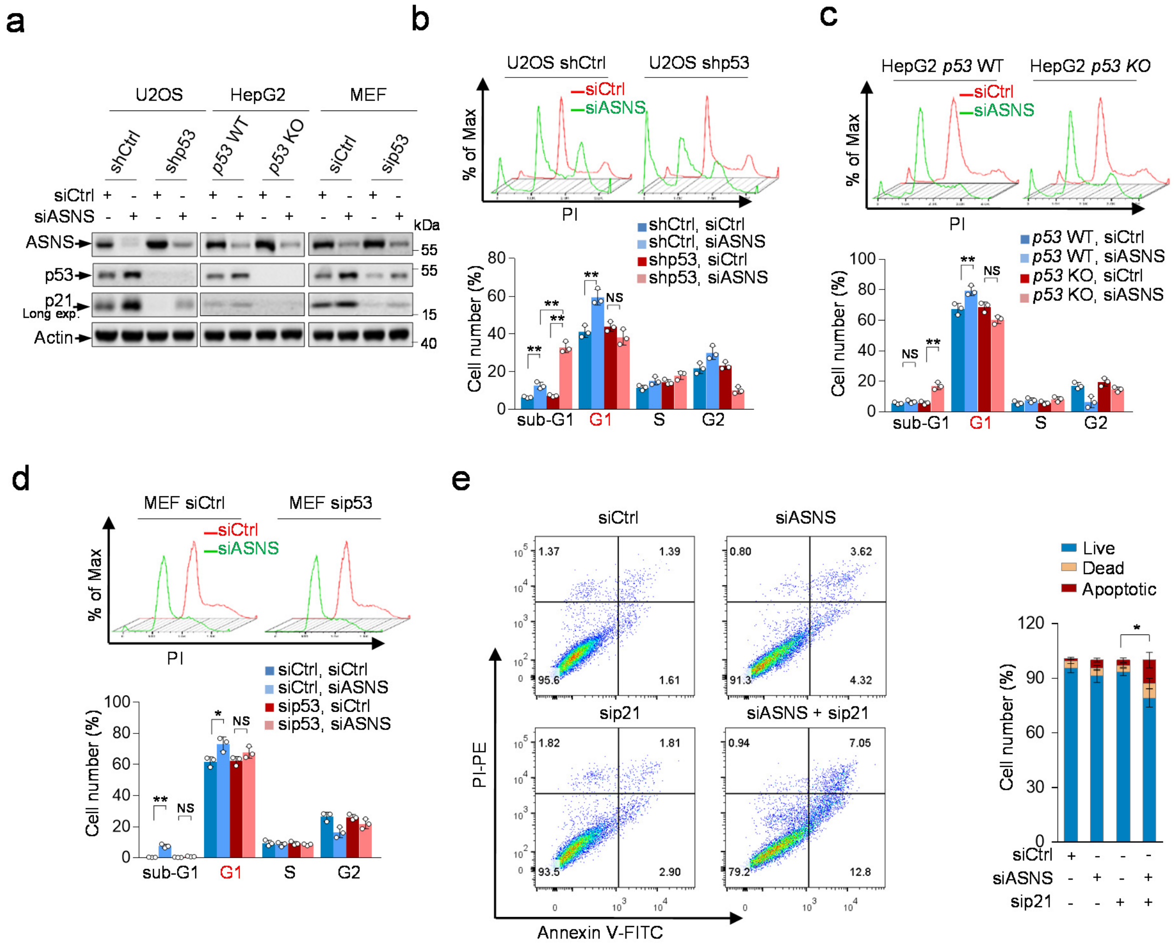

Supplementary Figure 8 | *ASNS* knockdown induces p53-dependent cell cycle arrest and promotes apoptosis in p53/p21-depeted cells. a-d, shCtrl and shp53 U2OS cells (**b**), p53-wildtype and p53-knockout HepG2 cells (**c**), or siCtrl and sip53 MEF cells (**d**) were transfected with control siRNA (siCtrl) or ASNS siRNA (siASNS) for 72 hours. Cell cycle distribution was determined and quantified (%) by PI staining and flow cytometry analysis. Data are representative of three independent experiments. Data are mean ± s.d. (n=3). Protein expression was determined by western blot analysis (**a**). **e**, HCT116 cells transfected with siCtrl, siASNS, and/or sip21 as indicated for 3 days were stained with annexin V and PI. Apoptosis was analyzed by flow cytometry analysis. Representative flow cytometry plots (left) and statistically quantitative results of live, dead and apoptotic cells (right) are shown. Data are mean ± s.d. (n=3). *p<0.05, **p<0.01, ***p <0.001, NS, not significant (Student's t-test). Source data are provided as a Source Data file.

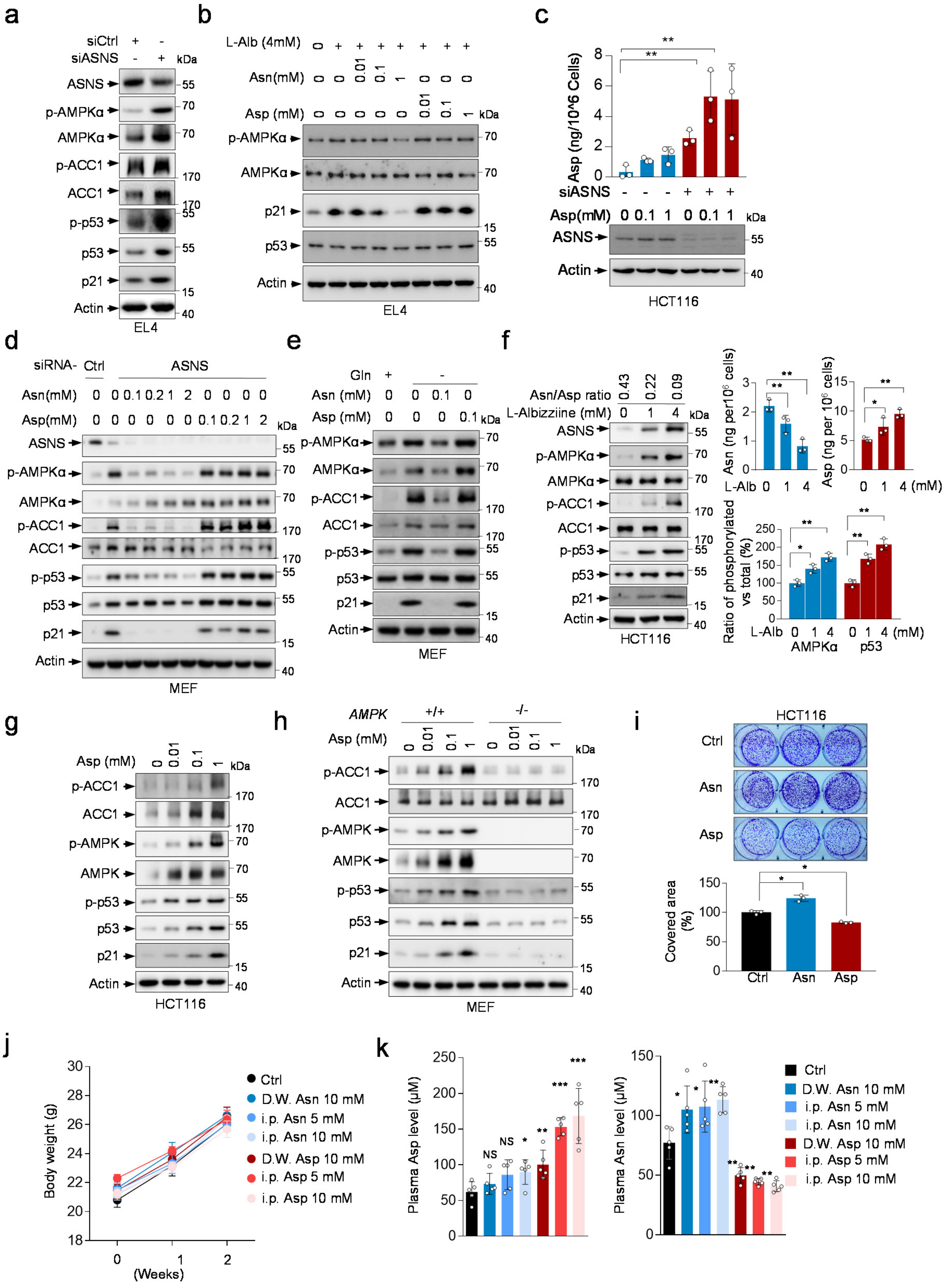

Supplementary Figure 9 | ASNS-mediated asparagine and aspartate homeostasis dictates AMPK signaling. a, Western blot analysis of EL4 cells transfected with siCtrl or siASNS for 3 days. Protein expression are indicated. **b**, EL4 cells treated with 4 mM L-Alb were cultured in medium containing increasing amounts of asparagine (Asn) or

aspartate (Asp) for 48 hours. Cell lysates were prepared and subjected to western blot analysis.**c**, HCT116 cells transfected with control siRNA (-) or ASNS siRNA were cultured in medium containing increasing amounts of aspartate (Asp) for 48 hours. Cellular aspartate levels and protein expression were measured. Data are mean ± s.d. (n=3). **d**, MEF cells transfected with control siRNA (siCtrl) or ASNS siRNA (siASNS) were treated with or without increasing amounts of asparagine (Asn) or aspartate (Asp) for 48 hours as indicated. Protein expressions are shown. **e**, Western blot analysis of lysates from MEF cells cultured in medium containing 3 mM glutamine, or in glutamine-free medium with or without 0.1 mM asparagine or aspartate for 48 hours. Protein expressions were analyzed by western blotting. **f**, HCT116 cells were treated with increasing amounts of L-Albizziine (L-Alb) for 2 days. Cellular asparagine (Asn) and aspartate (Asp) were determined and quantified by LC-MS individually (left right panel). The rations of Asn/Asp are shown. Protein expressions were determined by western blot analysis (left panel) and ratios of phosphorylated AMPK and total AMPK, and phosphorylated p53 and total p53 are shown (bottom right panel).. Data are mean ± s.d. (n=3). **g**, Lysates from HCT116 cells treated with increasing concentrations of Asp for 48 hours were analyzed by western blot. Data are representative of three independent experiments.**h**, Western blot analysis of $AMPK^{+/+}$ and $AMPK^{-/-}$ MEF cells treated with increasing concentrations of aspartate (Asp) for 48 hours. **i**, Proliferation of HCT116 cells cultured in Medium containing Asn or Asp for 5 days. Data are mean ± s.d. (n=3). **j**, Body weight of C57BL/6J mice treated as in Figure 6**e**. **k**, Levels of Plasma asparagine (Asn) and aspartate (Asp) in of C57BL/6J mice treated as in Figure 6**e** were determined and quantified by LC-MS. Each group includes 5 mice and 10 tumors. Data are mean ± s.d. *p<0.05, **p<0.01, ***p <0.001, NS, not significant (Student's t-test). Source data are provided as a Source Data file.

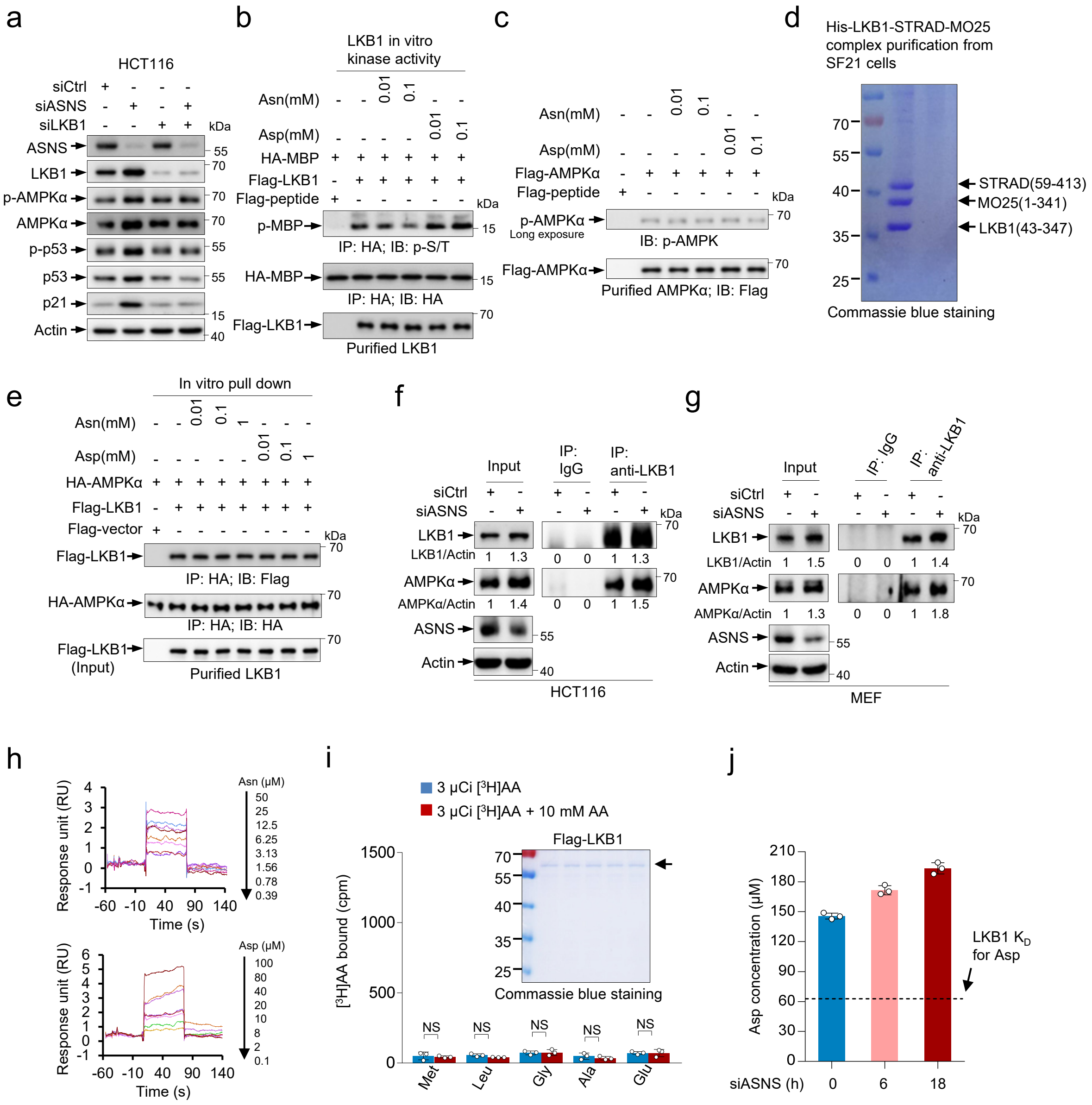

Supplementary Figure 10 | Asparagine and aspartate physically bind to LKB1. a, HCT116 cells transfected with siCtrl, siASNS and/or siLKB1 as indicated for 3 days. Cell lysates were prepared for western blot analysis. **b**, In vitro LKB1 kinase activity was determined using purified Flag-LKB1 incubated with purified HA-MBP as substrates in the presence or absence of increasing concentrations of Asn or Asp at 30℃ for 30 min. LKB1, AMPKα and MBP were purified from HEK-293 cells transiently expressing the indicated proteins separately. Samples were immunoprecipitated with anti-HA agarose, and analyzed by western blot. **c**, Flag-AMPKα were purified form HEK-293T cells transiently transfected with pRK5-Flag-AMPKα construct, and incubated with increasing amounts of as asparagine (Asn) or aspartate (Asp) at 30 ℃ for 30 min. AMPK phosphorylation and total AMPK levels were determined by western blot analysis. **d**, Purified His-LKB1-STRAD-MO25 complex from SF21 cells were

subjected to SDS-PAGE analysis and commassie blue staining. **e**, Flag-LKB1 and HA-AMPKα proteins were purified separately and mixed together in vitro in the presence or absence of increasing concentrations of asparagine (Asn) or aspartate (Asp) at room temperature for 4 hours, followed by immunoprecipitation of HA-AMPKα using anti-HA agarose and western blot to detect co-immunoprecipitated Flag-LKB1. **f** and **g**, Cell lysates from HCT116 cells (**f**) and MEF cells (**g**) transfected with control siRNA (siCtrl) or ASNS siRNA (siASNS) for 3 days were immunoprecipitated with an anti-LKB1 antibody and an isotype control antibody (IgG). Immunoprecipitated proteins and 5% input were analyzed by western blot. Relative LKB1/Actin and AMPKα/Actin ratios are shown. **h**, Surface plasmon resonance measurement of the interaction between purified LKB1 and Asn (up panel) or Asp (bottom panel). Graphs of equilibrium response unit responses (RU) and compound concentrations are plotted. Related to **Figure 7g**. **i**, LKB1 were purified from HEK-293T cells transiently transfected the Flag-tagged LKB1 construct and incubated with indicated radiolabeled amino acids individually. Unlabeled amino acid was added where indicated. Please see Methods for details. Data are mean ± s.d. (n=3). Purified HA-LKB1 protein was analyzed by SDS-PAGE followed by Coomassie Blue staining. **j**, HCT116 cells were treated with ASNS siRNA for 0, 6 and 18 hours as indicated before sample preparation for liquid chromatography/mass spectrometry (LC/MS)–based analysis of the absolute amounts of aspartate. The dissociation constant K_D of LKB1 for asparagine is indicated. *p<0.05, **p<0.01, ***p <0.001, NS, not significant (Student's t-test). Source data are provided as a Source Data file.

Supplementary Fig. 11

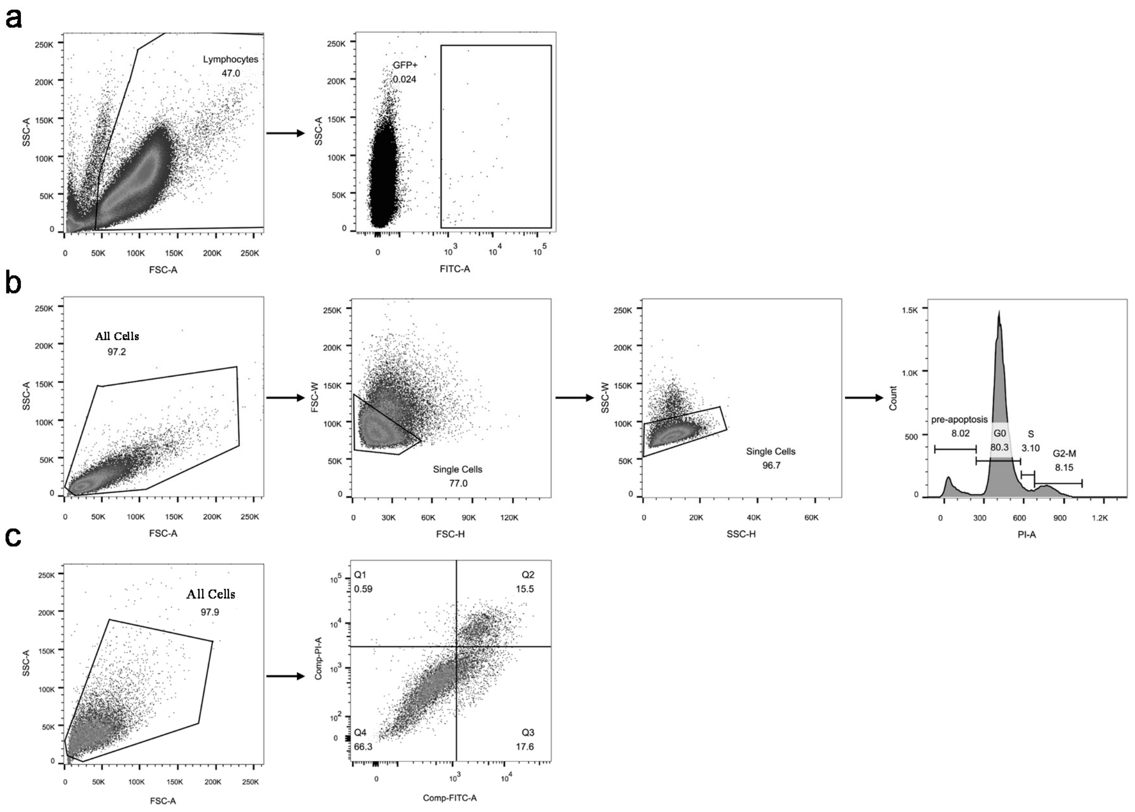

Supplementary Figure 11 | Gating strategies used in FACS analysis. a, Gating strategy to identify EL4-Luc-GFP cells (GFP^+) in peripheral blood in recipient mice presented on Fig. 1b and Supplemental Fig. 1a. **b**, Gating strategy to analyze PI-stained cells in cell cycle distribution analysis presented on Fig. 4h-j, Supplemental Fig. 7e-f and 8b-d. **c**, Gating strategy to analyze annexin V/PI double-stained cells in apoptosis analysis presented on Supplementary Fig. 7g and 8e.

Figure 2C

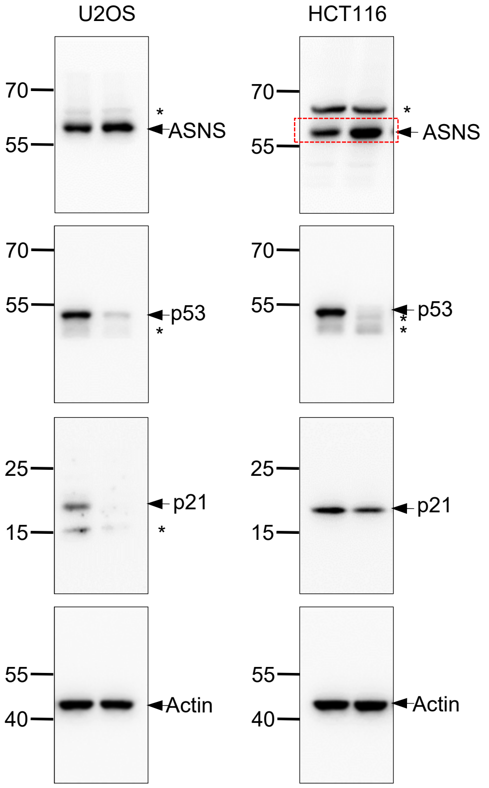

Figure 2D

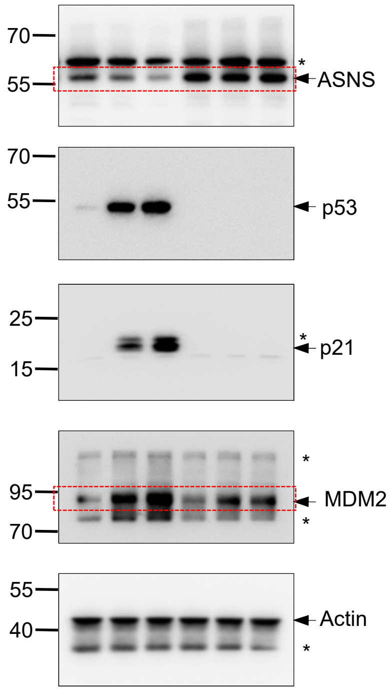

Figure 2G

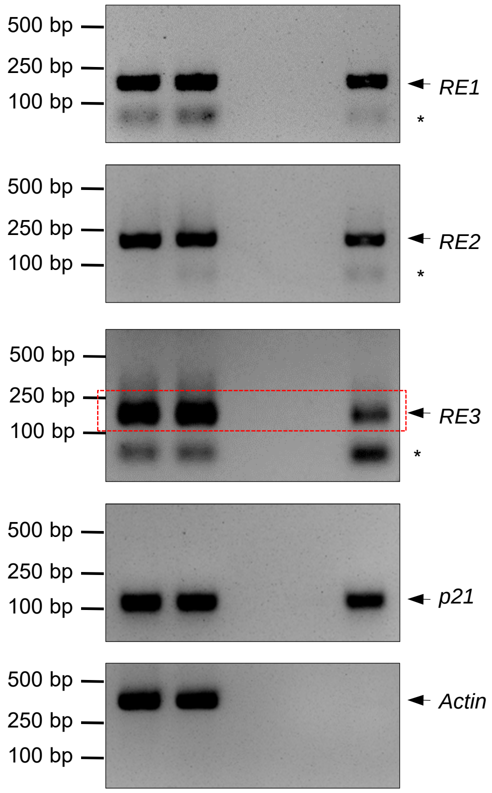

Figure 2E

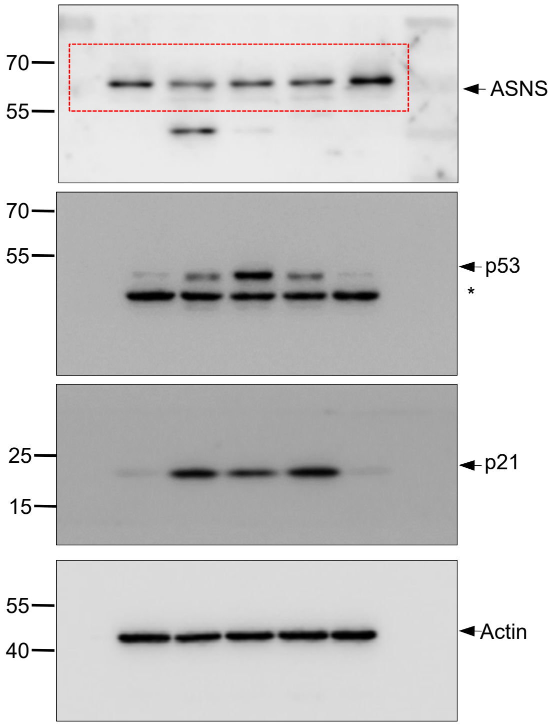

* Non-specific bands

Figure 3C

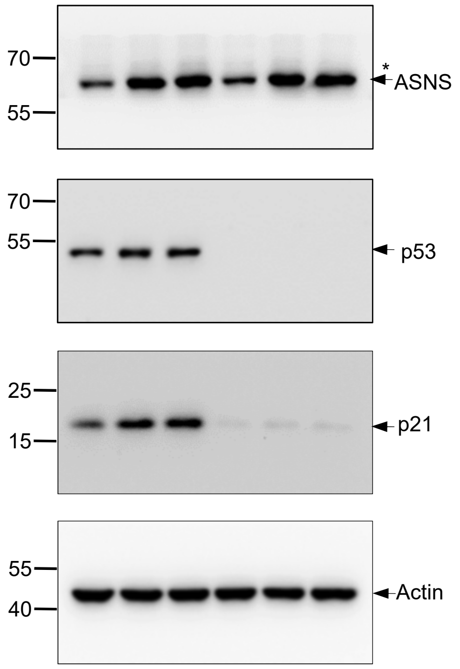

Figure 3E

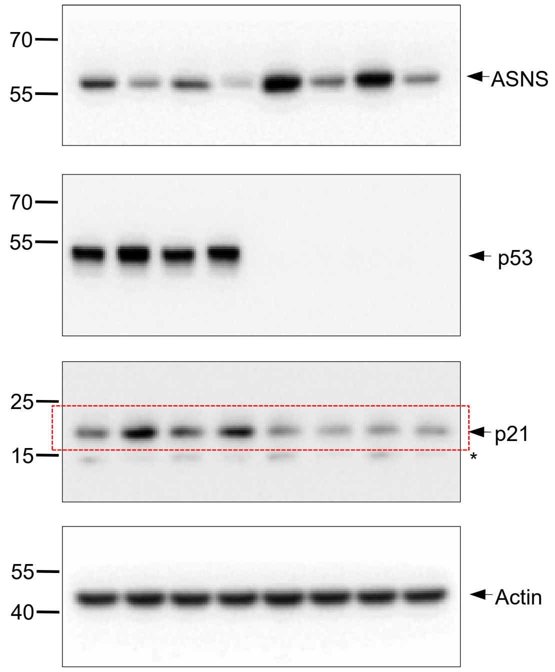

Figure 3I

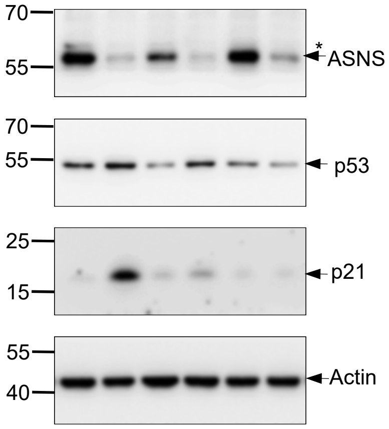

Figure 3J

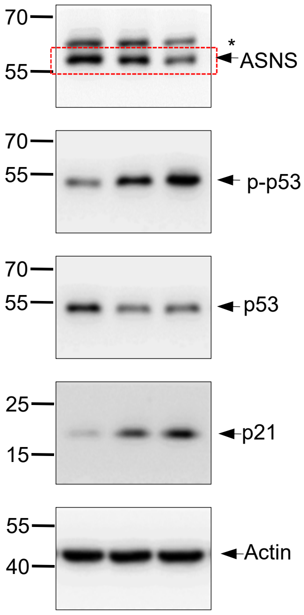

* Non-specific bands

Figure 4B

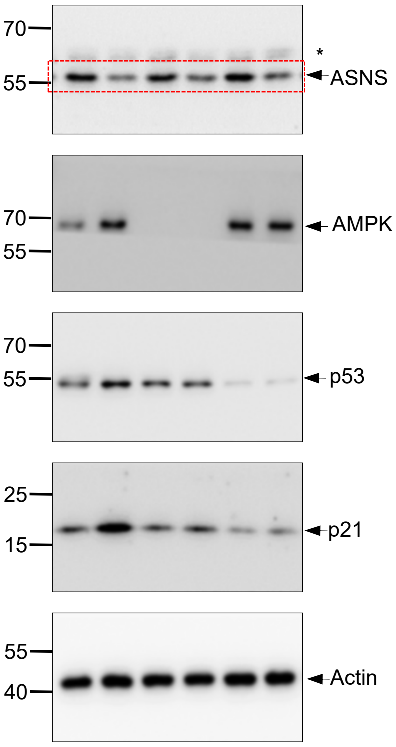

Figure 4C

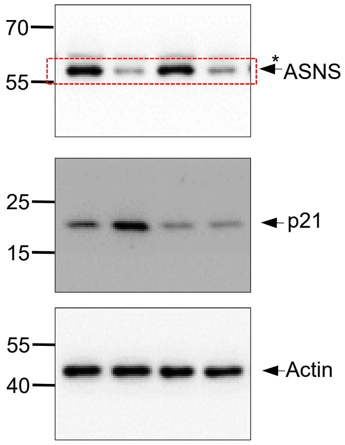

Figure 4D

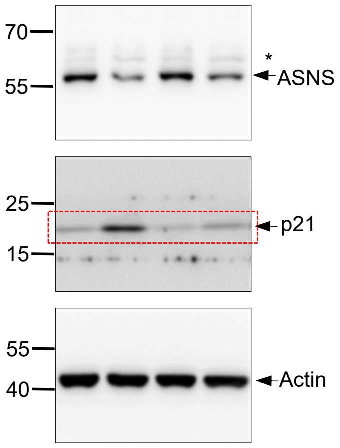

* Non-specific bands

Figure 4E

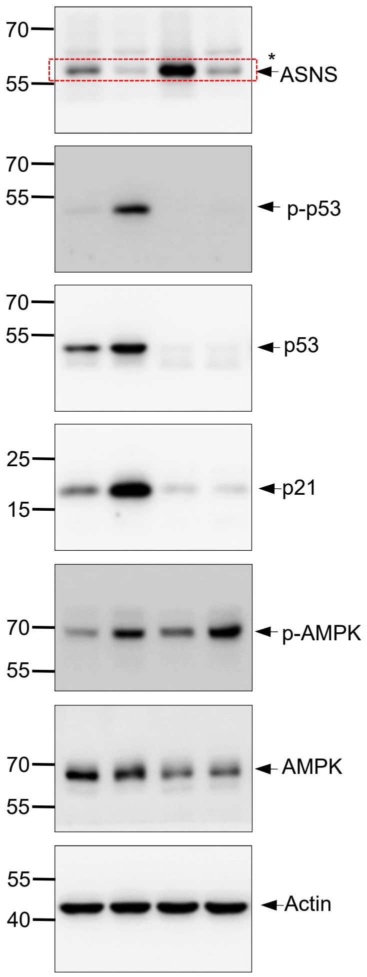

Figure 4F

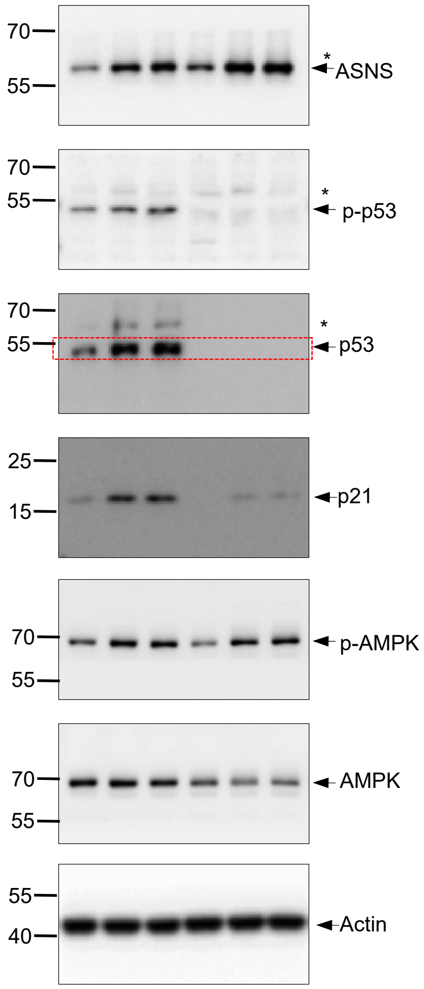

Figure 4G

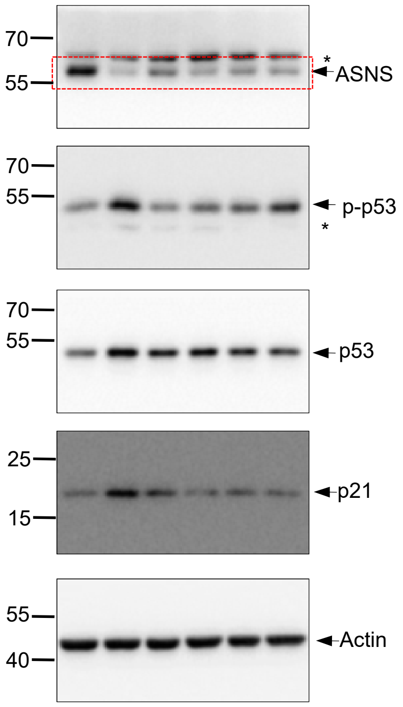

* Non-specific bands

Figure 5A and C

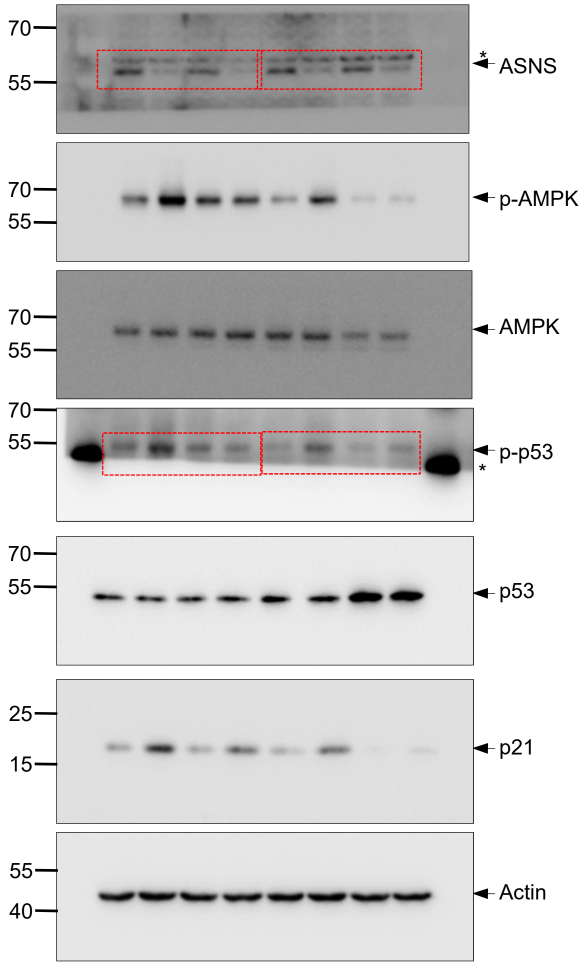

Figure 5B

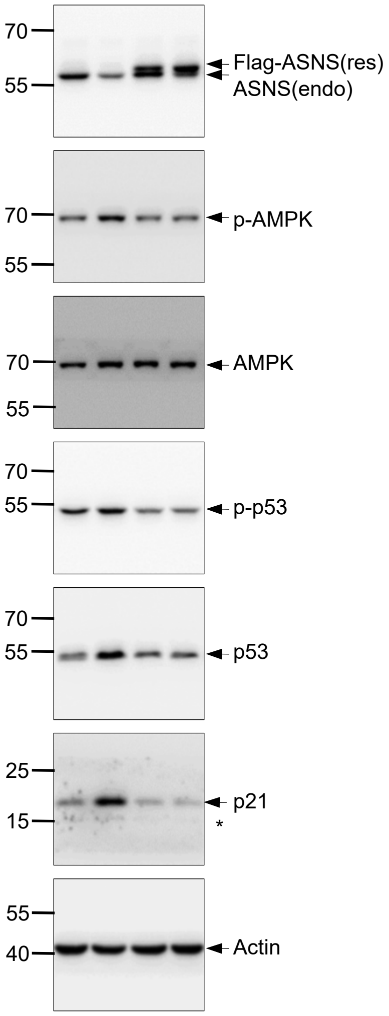

Figure 5D

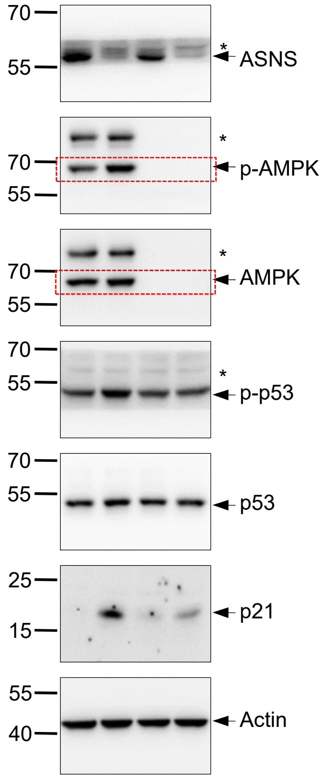

Figure 5E

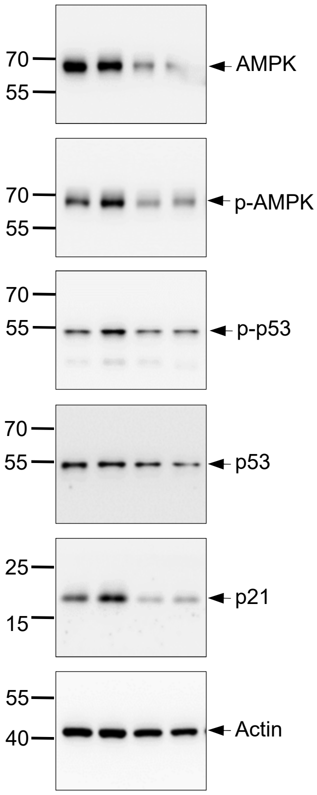

* Non-specific bands

Figure 5G

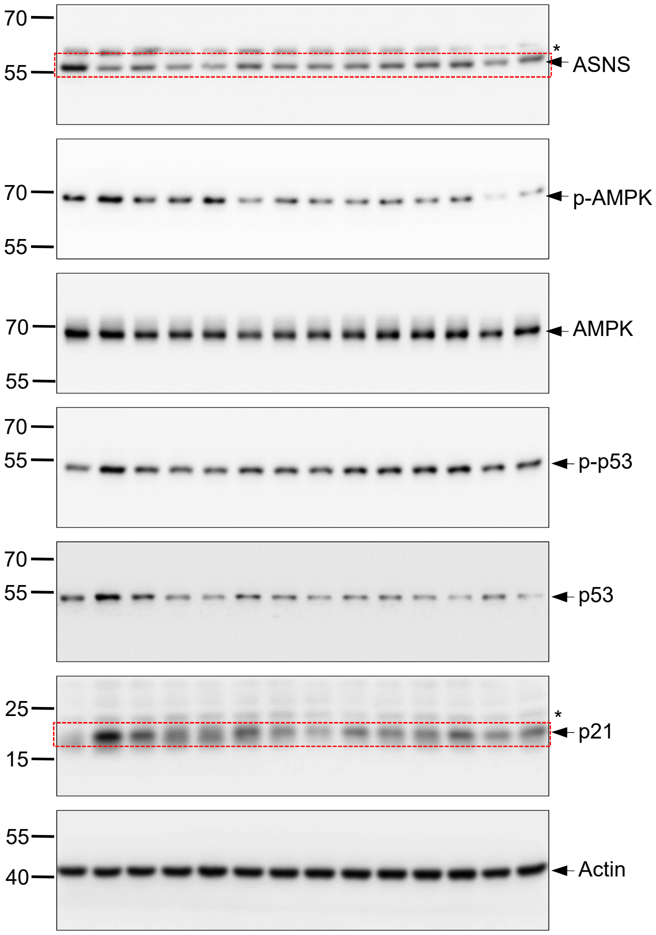

Figure 5H

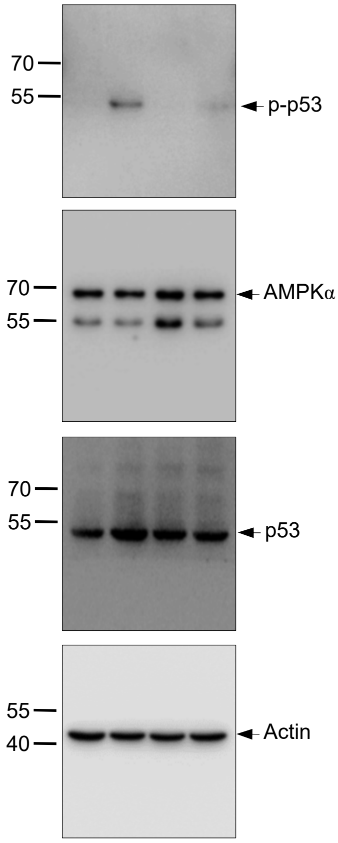

* Non-specific bands

Figure 5F

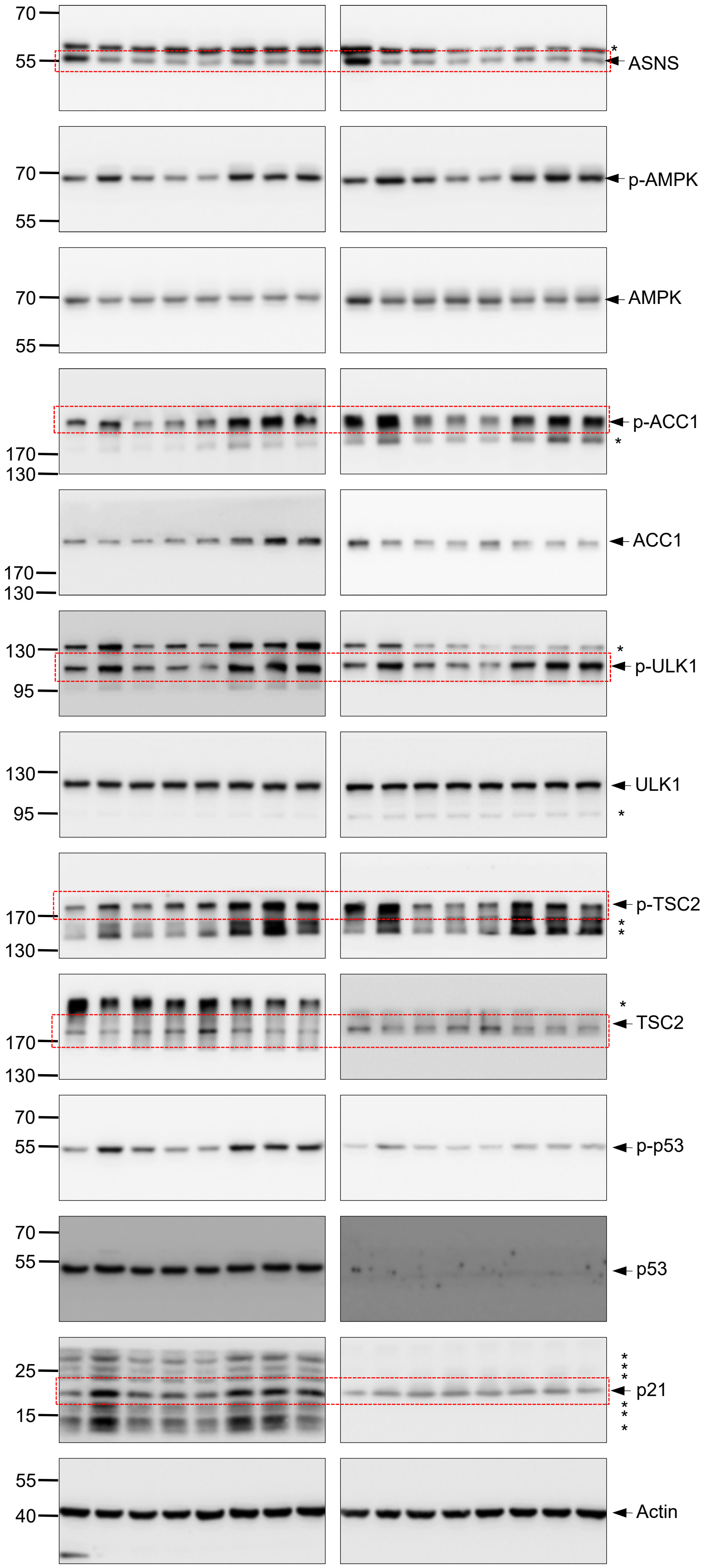

* Non-specific bands

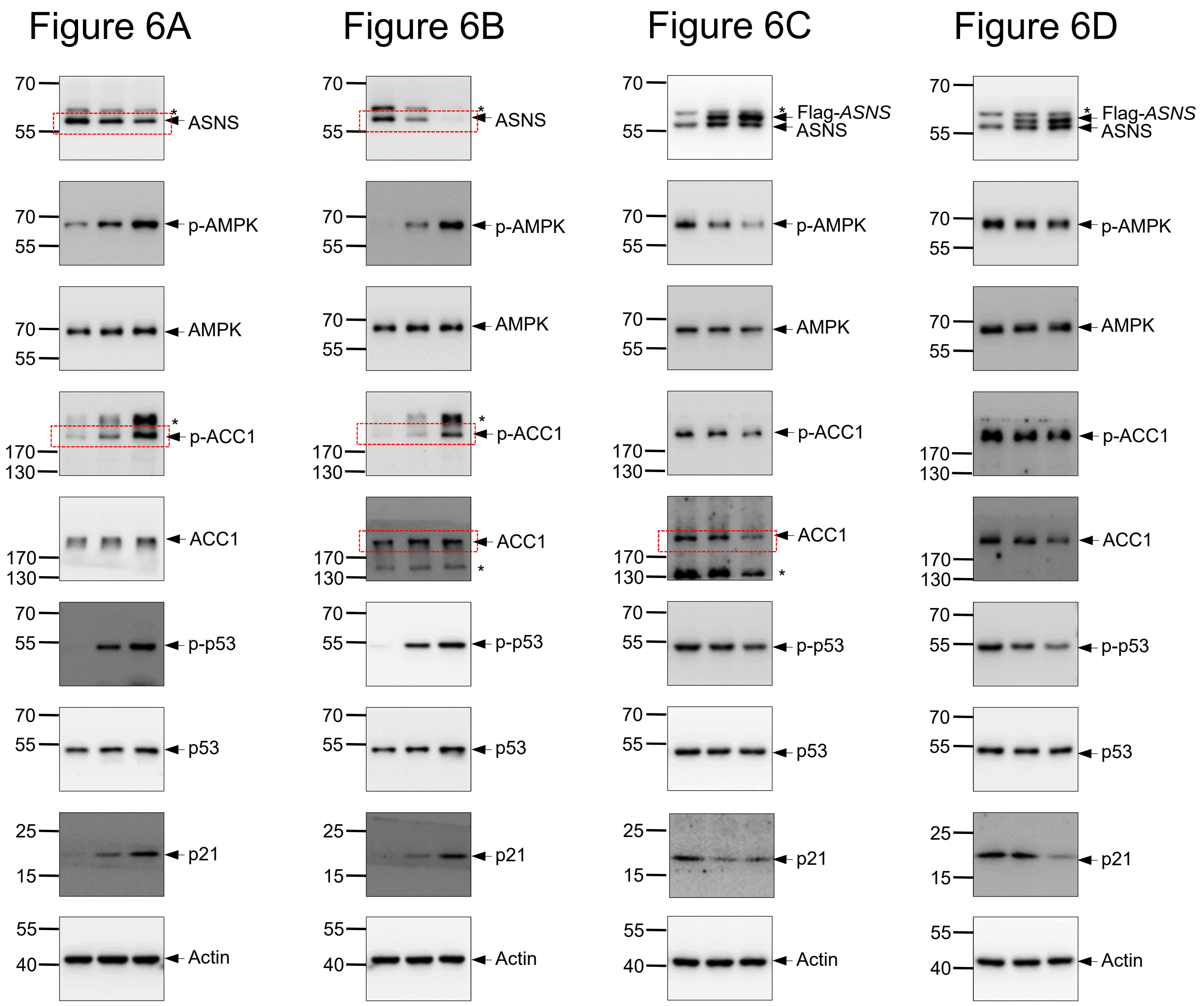

* Non-specific bands

Figure 6F

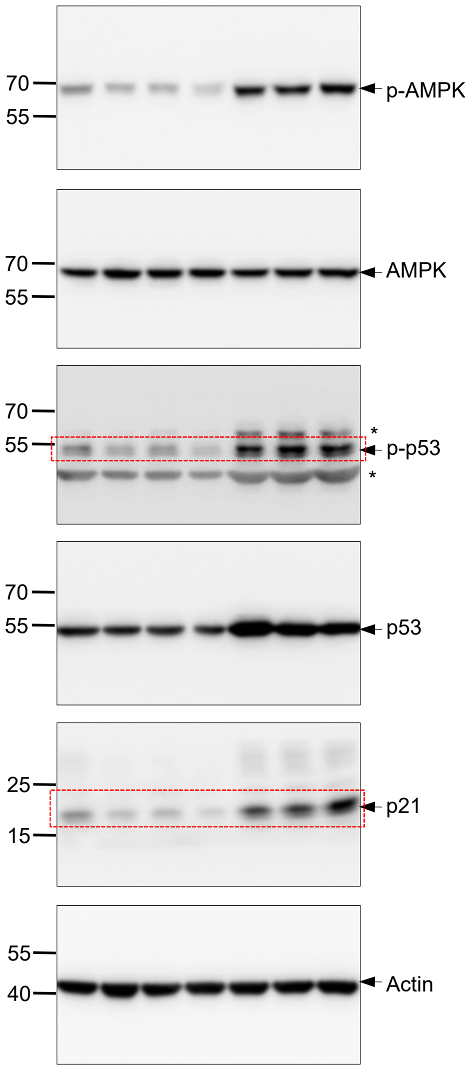

Figure 7A

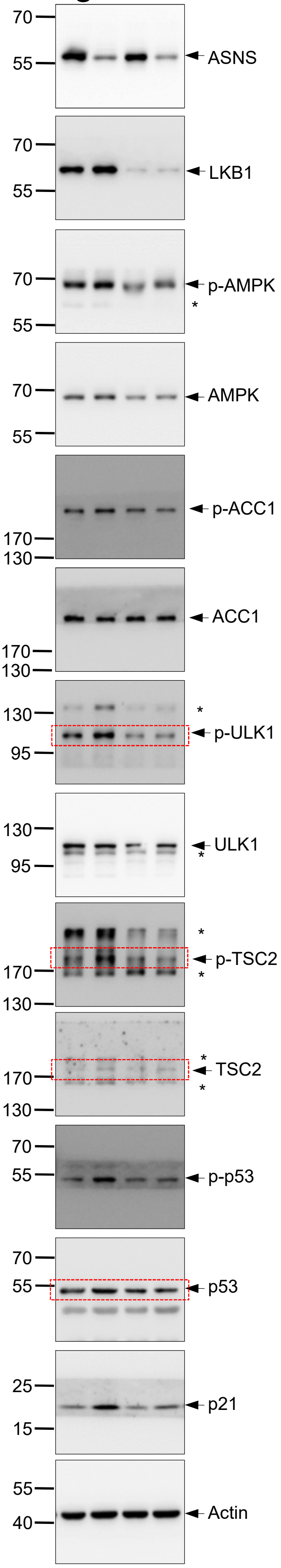

Figure 7B

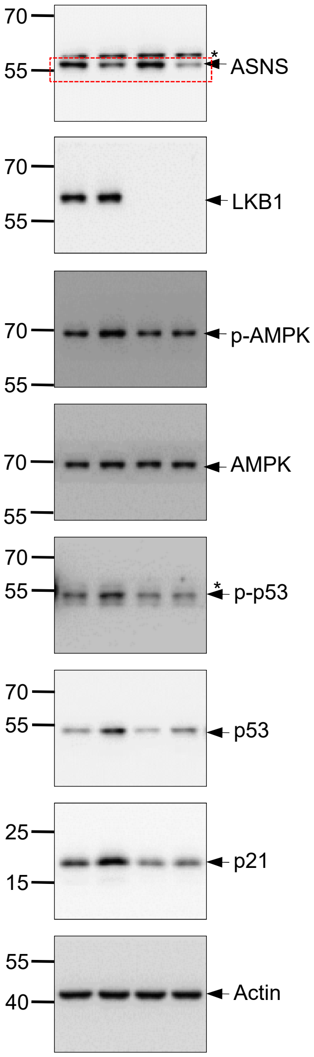

* Non-specific bands

Figure 7C

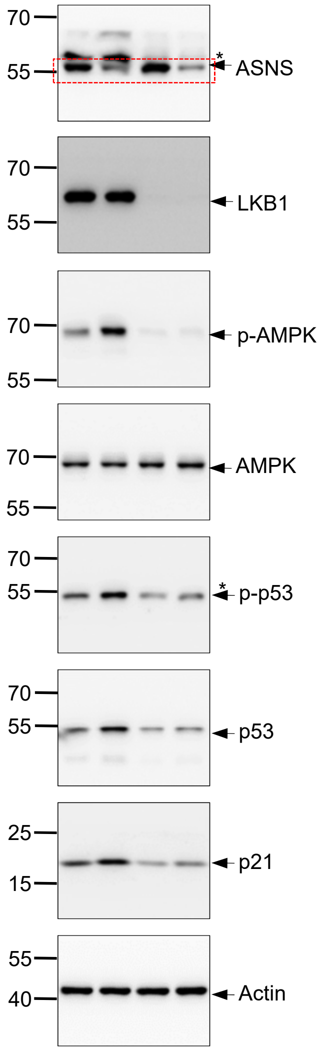

Figure 7D

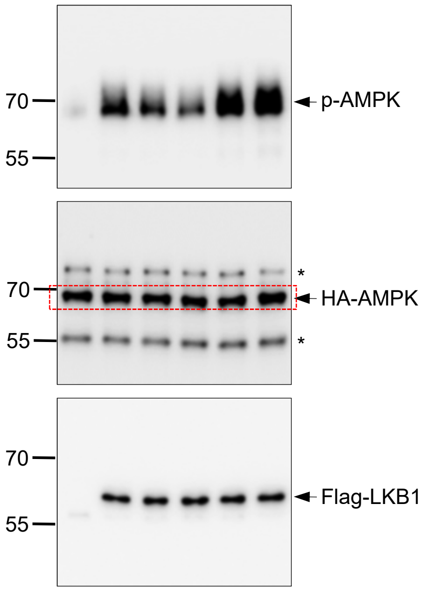

Figure 7E

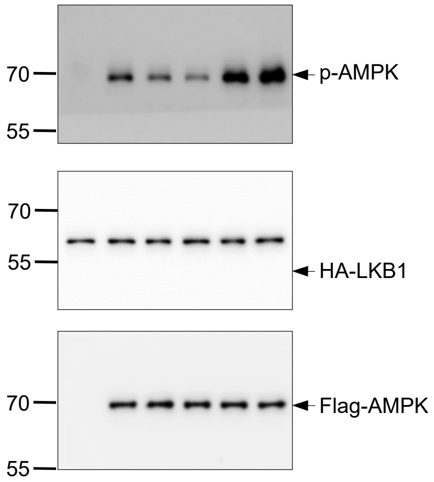

Figure 7F

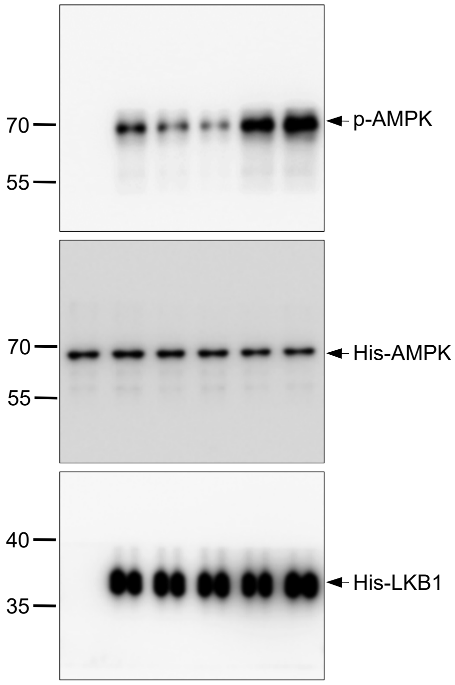

* Non-specific bands

Figure S2H

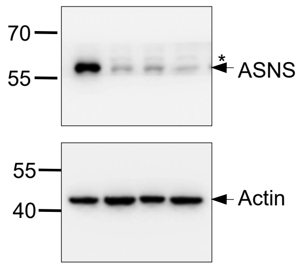

Figure S3A

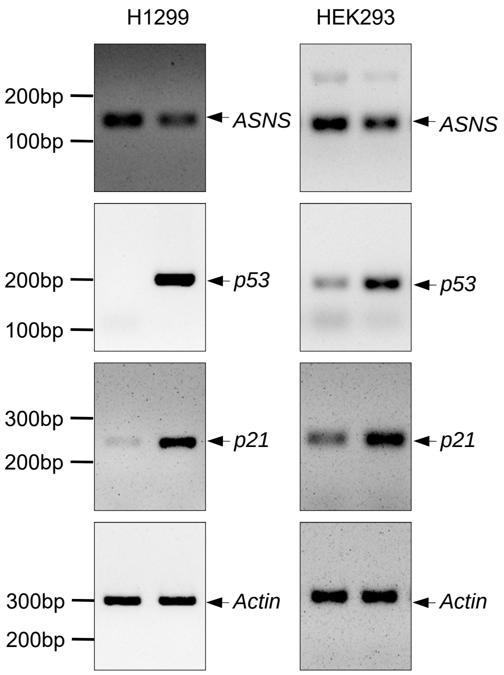

Figure S3B

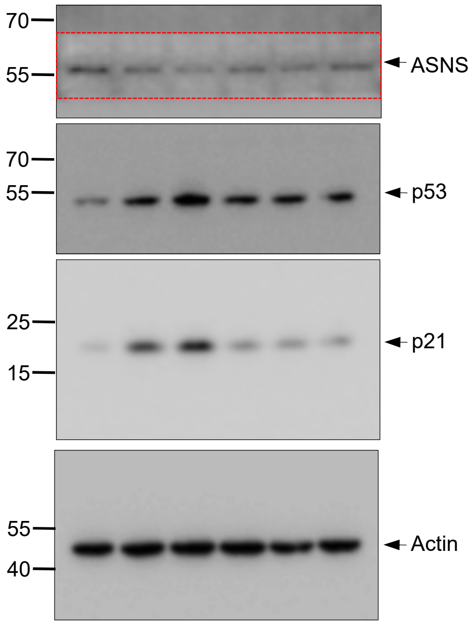

Figure S3C

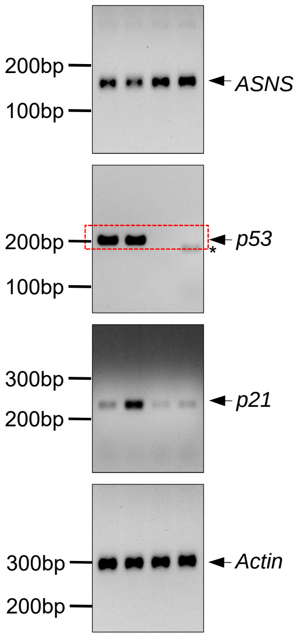

Figure S3D

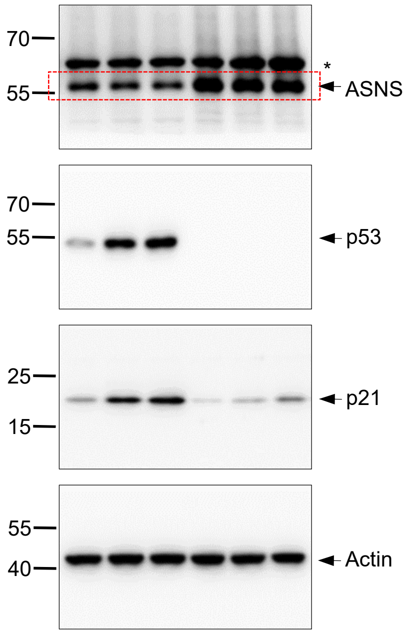

* Non-specific bands

Figure S3E

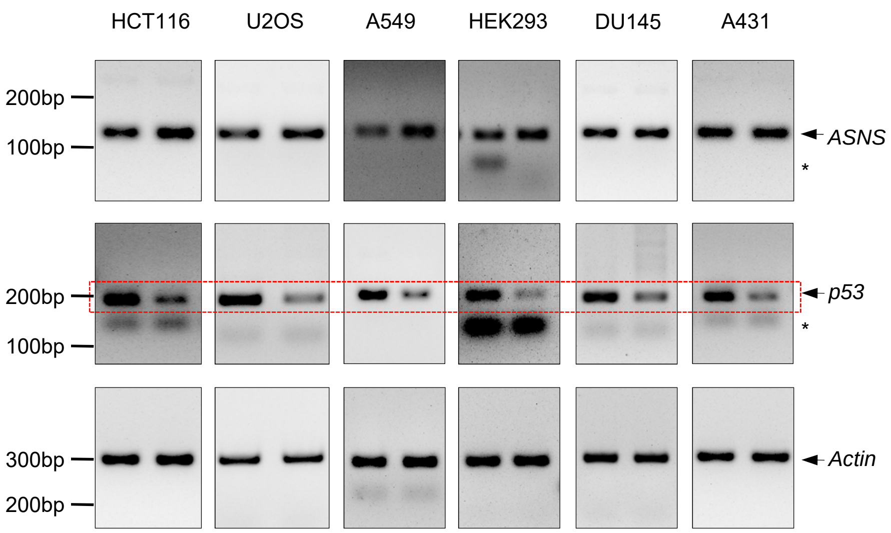

Figure S3F

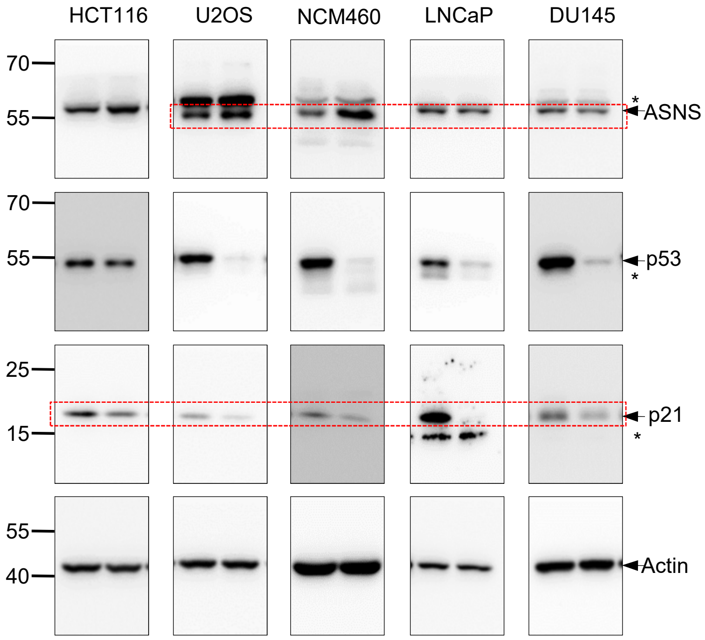

* Non-specific bands

Figure S3G

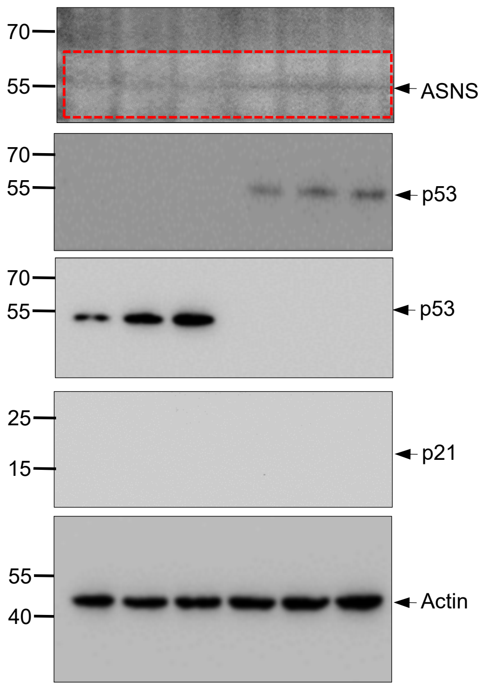

Figure S3H

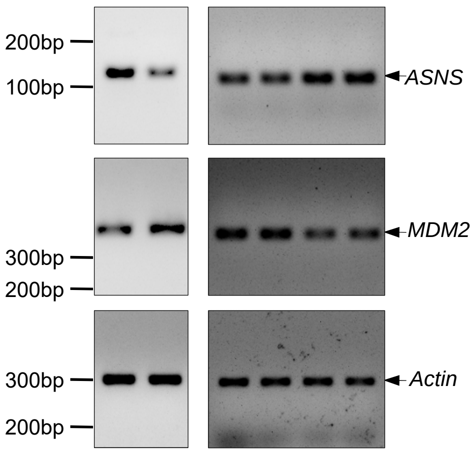

Figure S3J

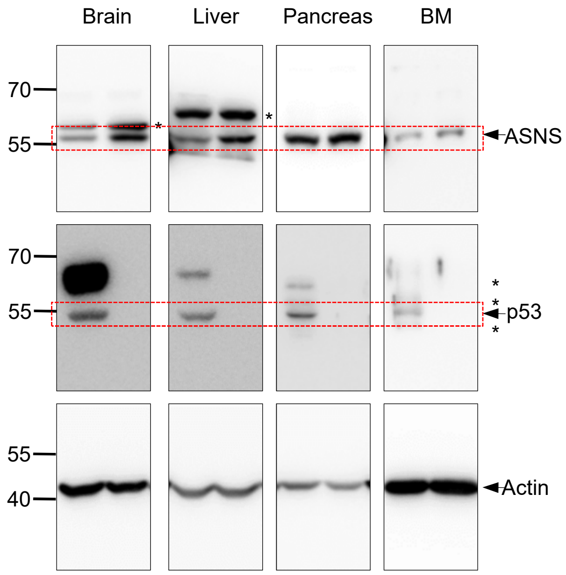

Figure S5B

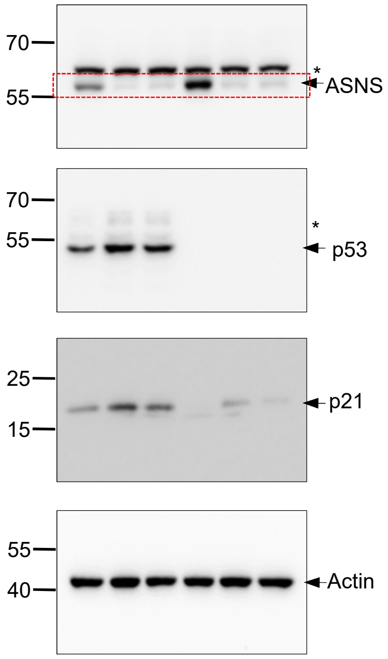

* Non-specific bands

Figure S5E

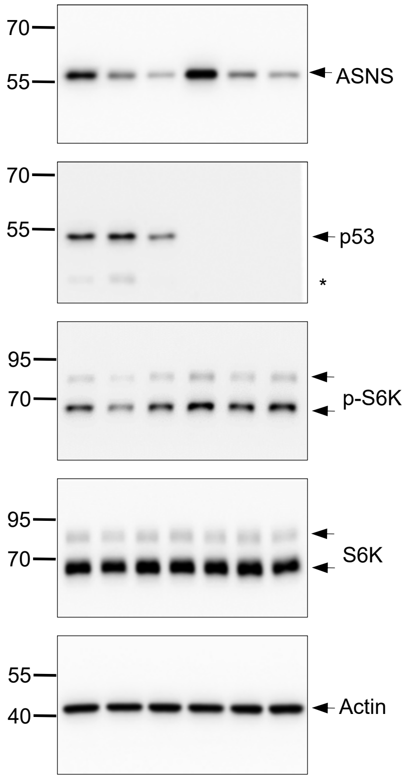

Figure S5F

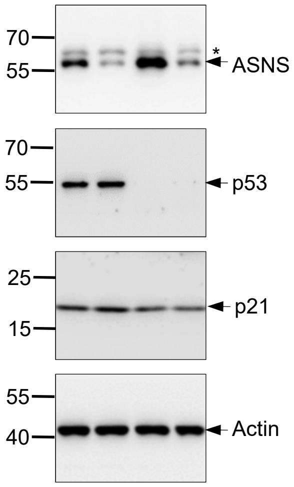

Figure S5K

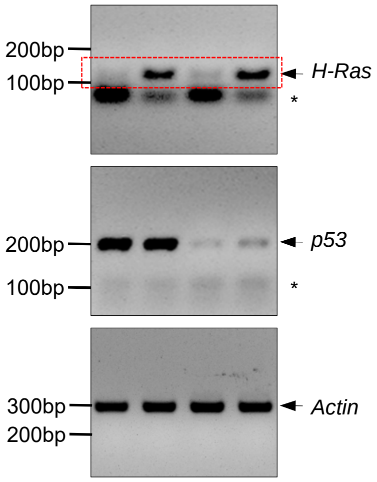

Figure S5L

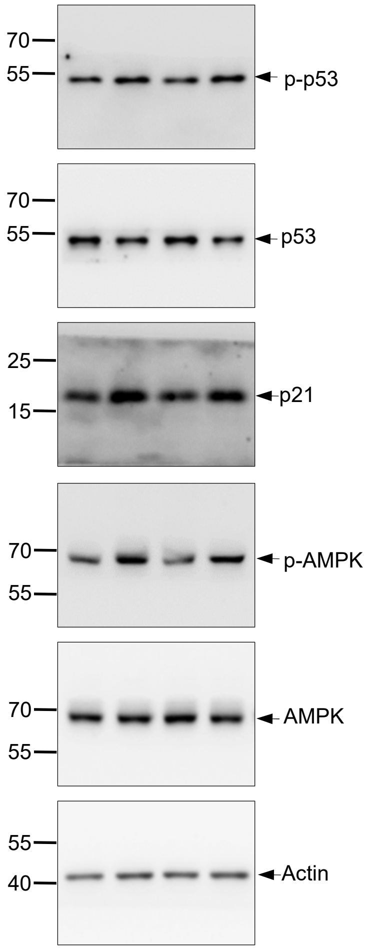

Figure S6C

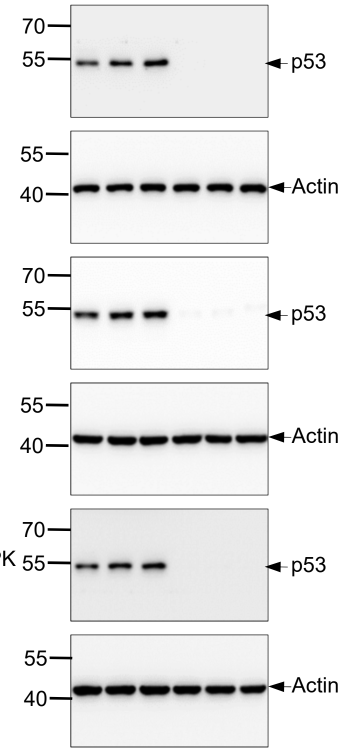

* Non-specific bands

Figure S6D

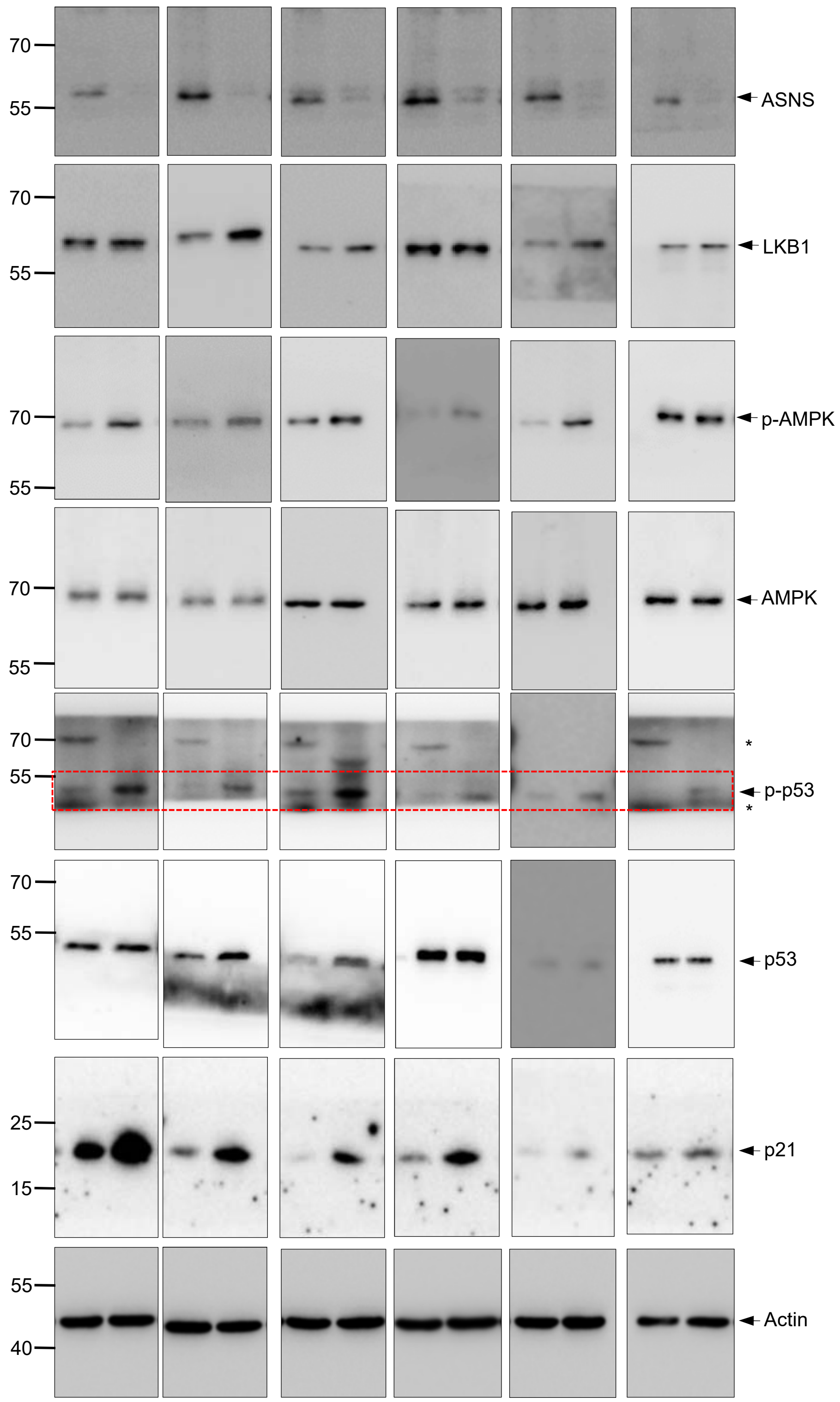

Figure 6E

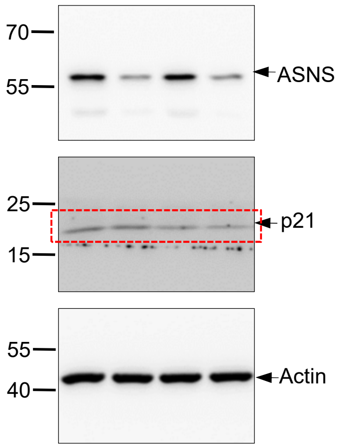

* Non-specific bands

Figure S7A

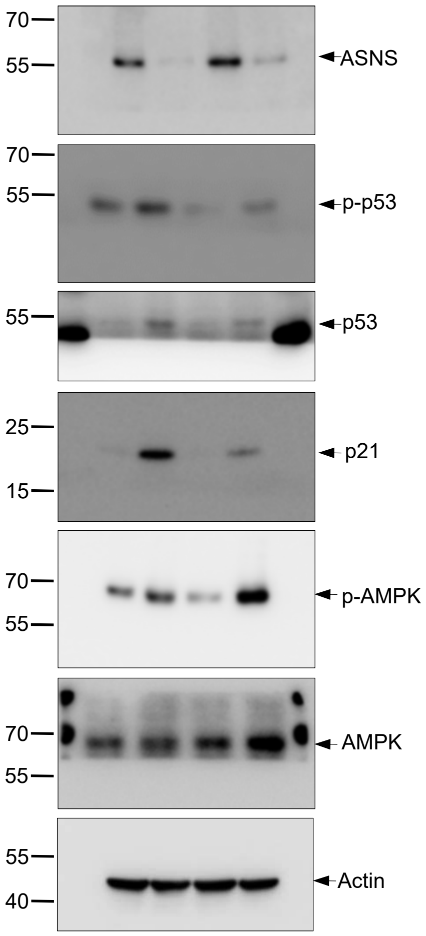

Figure S7B

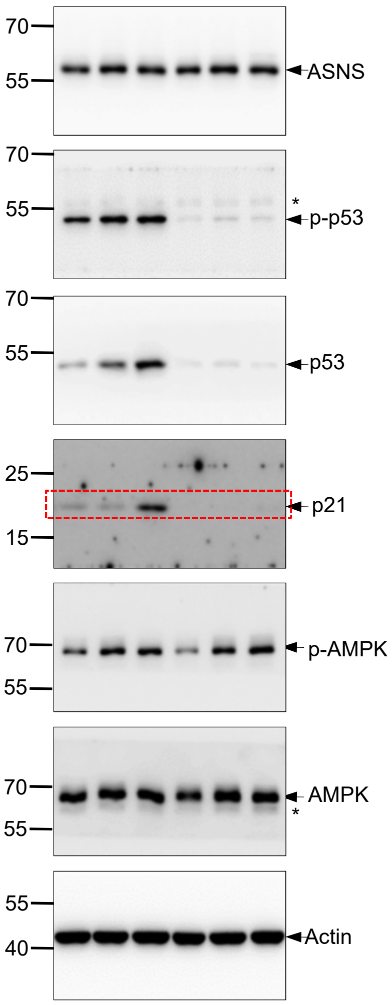

Figure S7C

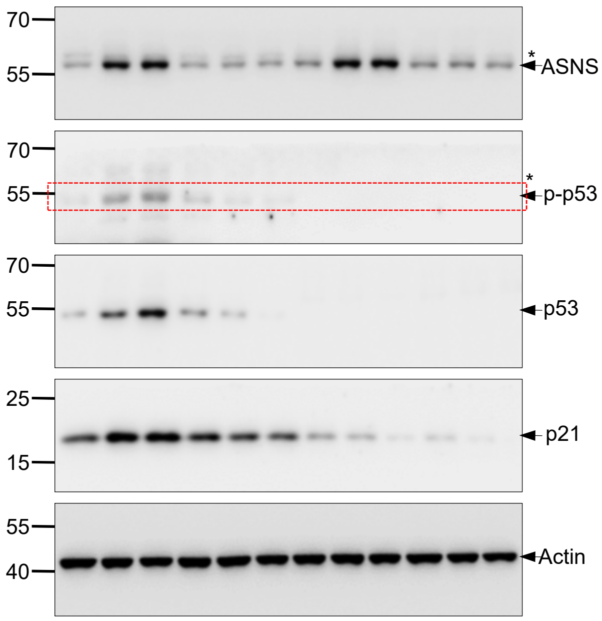

Figure S7D

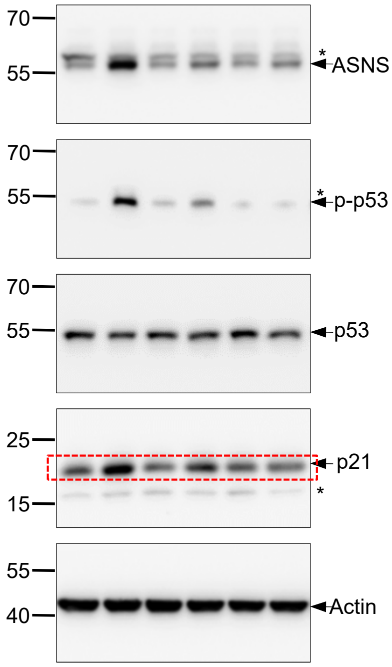

Figure S8A

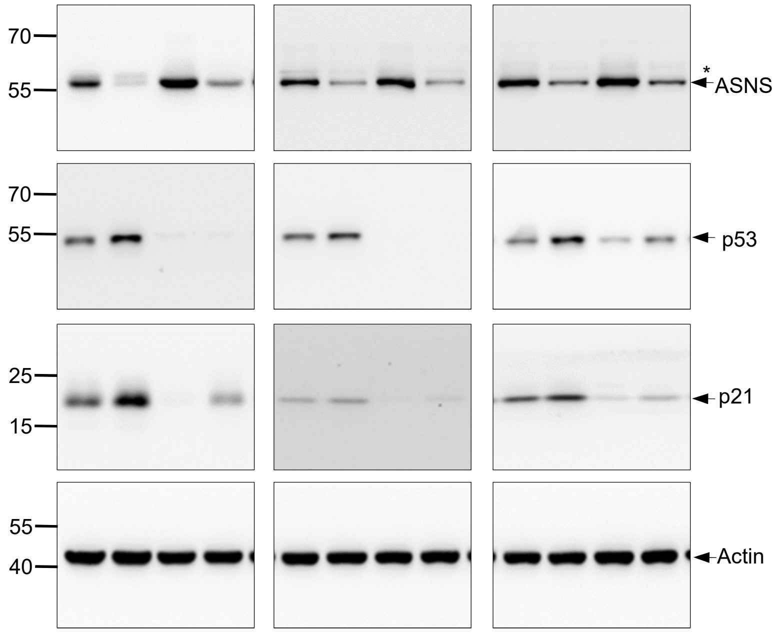

* Non-specific bands

Figure S9A

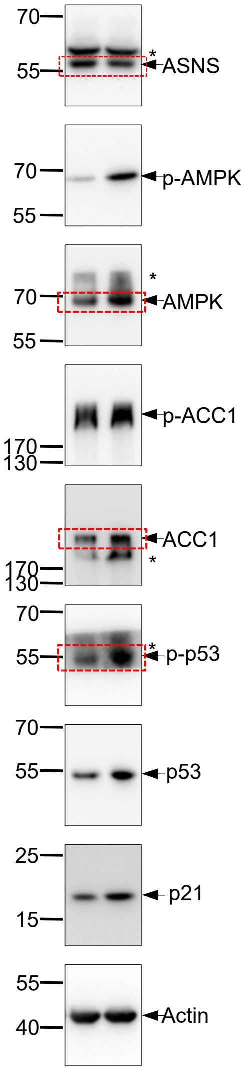

Figure S9B

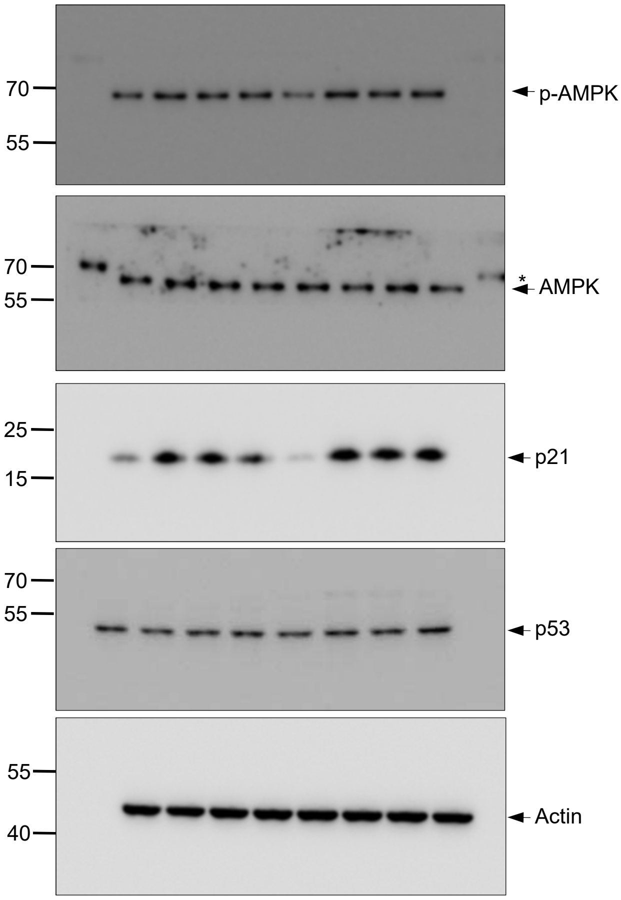

Figure S9C

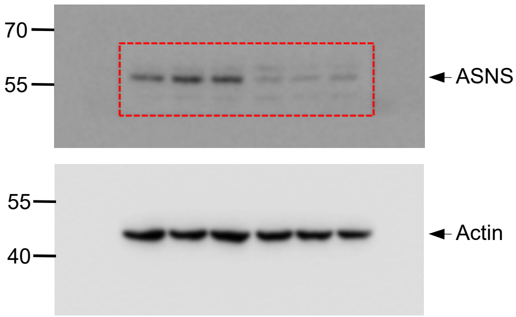

* Non-specific bands

Figure S9D

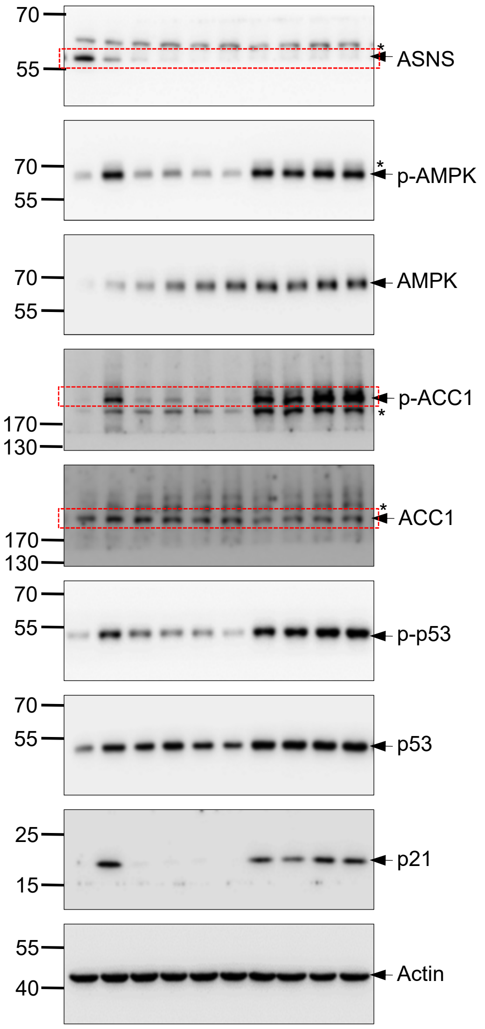

Figure S9E

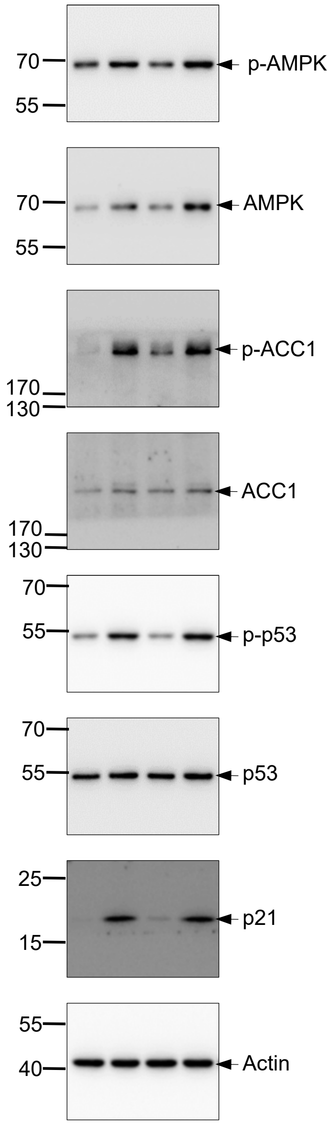

Figure S9F

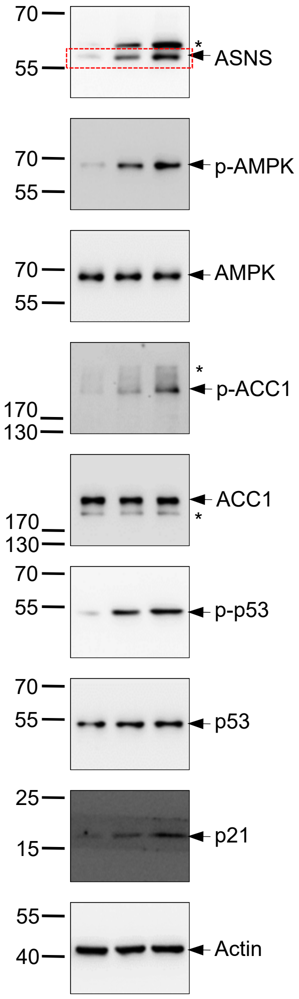

* Non-specific bands

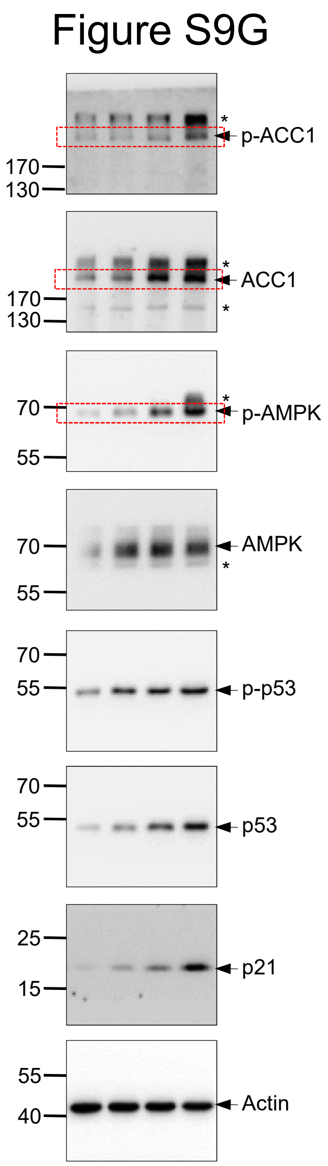

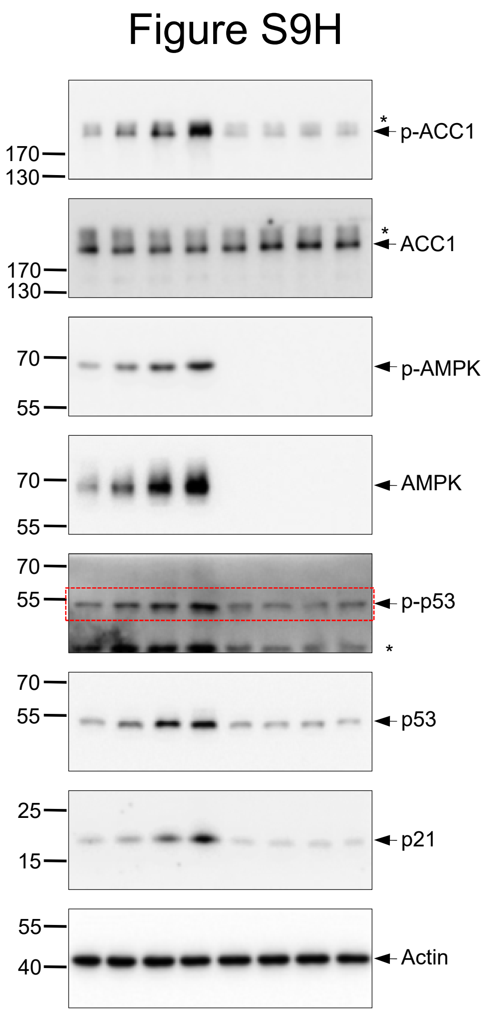

* Non-specific bands

Figure S10A

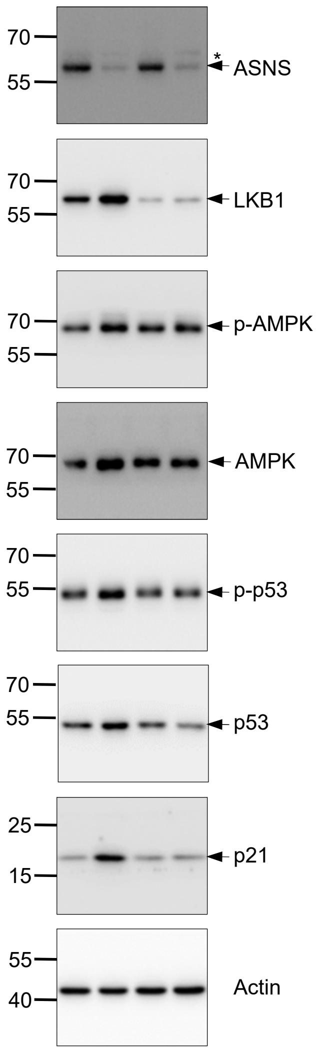

Figure S10B

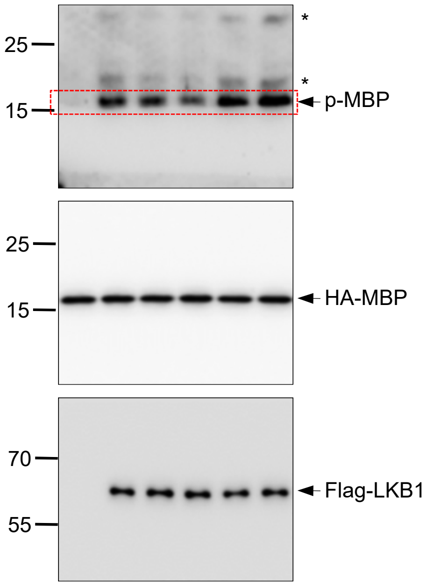

Figure S10C

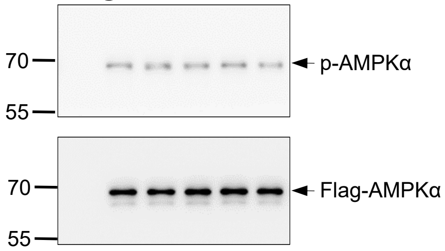

Figure S10D

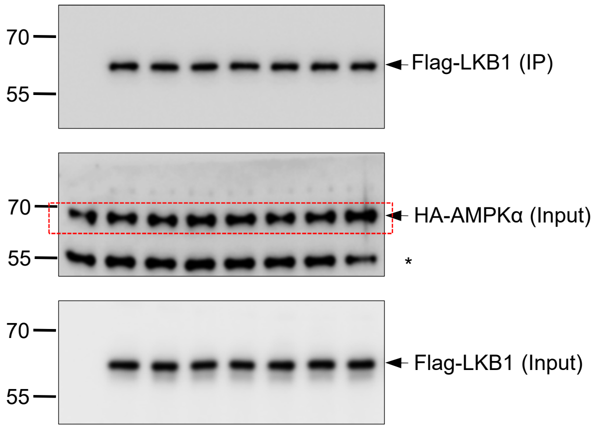

Figure S10F

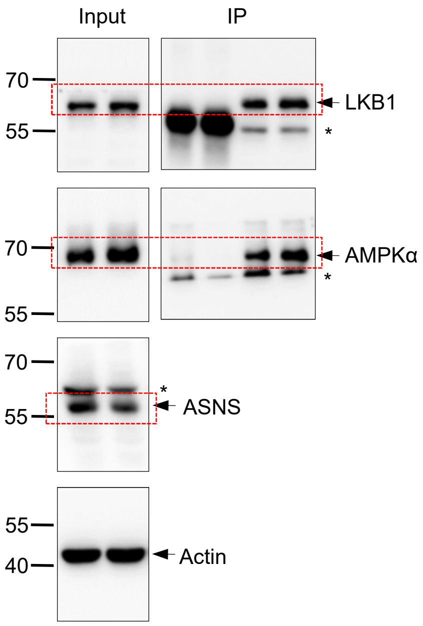

Figure S10G

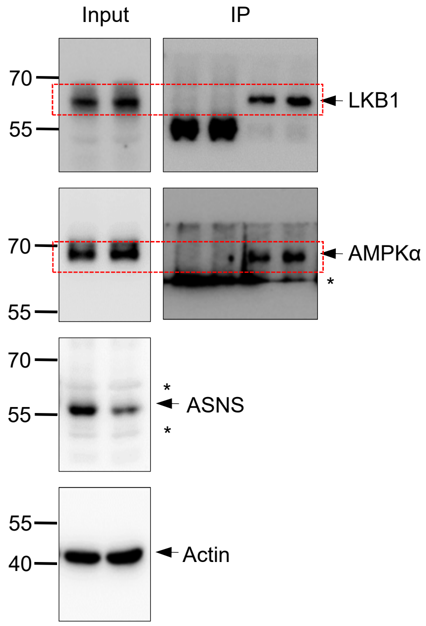

* Non-specific bands
