# Supplementary material for: p53-mediated control of aspartate-asparagine homeostasis dictates LKB1 activity and modulates cell survival
Source: Nat Commun. 2020 Apr 9;11:1755. doi: 10.1038/s41467-020-15573-6 (PMC7145870; doi:10.1038/s41467-020-15573-6)
Supplement: Supplementary file 2 — Reporting Summary [file 41467_2020_15573_MOESM2_ESM.pdf]

nature research

Corresponding author(s): Peng Jiang

Last updated by author(s): Feb 6, 2020

Reporting Summary

Nature Research wishes to improve the reproducibility of the work that we publish. This form provides structure for consistency and transparency in reporting. For further information on Nature Research policies, see Authors & Referees and the Editorial Policy Checklist .

Statistics

For all statistical analyses, confirm that the following items are present in the figure legend, table legend, main text, or Methods section.

n/a	Confirmed	
[ ]	[x]	The exact sample size (*n*) for each experimental group/condition, given as a discrete number and unit of measurement
[ ]	[x]	A statement on whether measurements were taken from distinct samples or whether the same sample was measured repeatedly
[ ]	[x]	The statistical test(s) used AND whether they are one- or two-sided<br>*Only common tests should be described solely by name; describe more complex techniques in the Methods section.*
[x]	[ ]	A description of all covariates tested
[ ]	[x]	A description of any assumptions or corrections, such as tests of normality and adjustment for multiple comparisons
[ ]	[x]	A full description of the statistical parameters including central tendency (e.g. means) or other basic estimates (e.g. regression coefficient) AND variation (e.g. standard deviation) or associated estimates of uncertainty (e.g. confidence intervals)
[ ]	[x]	For null hypothesis testing, the test statistic (e.g. *F*, *t*, *r*) with confidence intervals, effect sizes, degrees of freedom and *P* value noted<br>*Give P values as exact values whenever suitable.*
[x]	[ ]	For Bayesian analysis, information on the choice of priors and Markov chain Monte Carlo settings
[x]	[ ]	For hierarchical and complex designs, identification of the appropriate level for tests and full reporting of outcomes
[ ]	[x]	Estimates of effect sizes (e.g. Cohen's *d*, Pearson's *r*), indicating how they were calculated

Our web collection on statistics for biologists contains articles on many of the points above.

Software and code

Policy information about availability of computer code

Data collection	We used the Genomatix Promoter Inspector software (http://www.genomatix.de) to search for potential TP53 response elements in ASNS gene. The sgRNAs used in this study were designed by CRISPER Design tool (crispr.mit.cn).
Data analysis	Cell cycle distribution was obtained using a LSR II cytometer (BD Biosciences, USA), and the data were analyzed using FlowJo software (TreeStar). Certain western blot bands were quantified using ImageJ software. All statistical analysis and P values were obtained using the GraphPad Prism software 7.0 (GraphPad Software, Inc. USA).

For manuscripts utilizing custom algorithms or software that are central to the research but not yet described in published literature, software must be made available to editors/reviewers. We strongly encourage code deposition in a community repository (e.g. GitHub). See the Nature Research guidelines for submitting code & software for further information.

Data

Policy information about availability of data

All manuscripts must include a data availability statement. This statement should provide the following information, where applicable:
- Accession codes, unique identifiers, or web links for publicly available datasets
- A list of figures that have associated raw data
- A description of any restrictions on data availability

All data are available from the corresponding author upon reasonable request.

Field-specific reporting

Please select the one below that is the best fit for your research. If you are not sure, read the appropriate sections before making your selection.

☒ Life sciences ☐ Behavioural & social sciences ☐ Ecological, evolutionary & environmental sciences

For a reference copy of the document with all sections, see nature.com/documents/nr-reporting-summary-flat.pdf

Life sciences study design

All studies must disclose on these points even when the disclosure is negative.

Sample size	No statistical test was used to determine sample size; Sample size was chosen based on literature.
Data exclusions	No data exclusion.
Replication	All replication attempts were successful.
Randomization	Randomization was not required for biochemical and in vitro experiments. In in vivo experiments, mice were randomly selected and allocated.
Blinding	The investigators were not blinded to allocation during experiments and data analysis, as objective quantitative assays were used when we generated the data.

Reporting for specific materials, systems and methods

We require information from authors about some types of materials, experimental systems and methods used in many studies. Here, indicate whether each material, system or method listed is relevant to your study. If you are not sure if a list item applies to your research, read the appropriate section before selecting a response.

Materials & experimental systems

n/a	Involved in the study	
☐	☒	Antibodies
☐	☒	Eukaryotic cell lines
☒	☐	Palaeontology
☐	☒	Animals and other organisms
☒	☐	Human research participants
☒	☐	Clinical data

Methods

n/a	Involved in the study	
☒	☐	ChIP-seq
☐	☒	Flow cytometry
☒	☐	MRI-based neuroimaging

Antibodies

Antibodies used	The information of the antibodies was listed below: ASNS (Proteintech, 14681-1-AP), p21 (BD Biosciences, 556431), p53 (DO-1)-HRP (Santa Cruz, sc126-HRP), p53 (Pab 1801) (Santa Cruz, sc-98), Phosphorylated-p53(Ser15) (Cell signaling technology, 9284S), AMPK (Cell signaling technology, 23A3), phospho-AMPK(Thr 172) (Cell signaling technology, 40H9), Acetyl-CoA Carboxylase (Cell signaling technology, C83B10), Phospho-Acetyl-CoA Carboxylase(Ser79) (Cell signaling technology, 3661), LKB1 (Cell signaling technology, 27D10), HA(HA-7)(Sigma H3663), FLAG(M2) (Sigma F3165), Actin (Proteintech, 66009-1-LG), Goat anti-rabbit IgG-HRP (Santa Cruz, sc-2004) and Goat anti-mouse IgG-HRP (Santa Cruz, sc-2302), ULK1 antibody [EPR4885(2)] (Abcam, ab128859), Phospho-ULK1 (Ser555) (D1H4) (Cell signaling technology, 5869), Tuberin/TSC2 (Cell signaling technology, 3612), Phospho-Tuberin/TSC2 (Ser1387) (Cell signaling technology, 5584), ATM [2C1 (1A1)] (Abcam, ab78), phospho-ATM (S1981) [EP1890Y] (Abcam, ab81292), Chk1 (Abcam, ab47574), Phospho-Chk1 (Ser345) (Cell signaling technology, 2341), p70 S6 Kinase (49D7) (Cell signaling technology, 2708), Phospho-p70 S6 Kinase (Thr389) (108D2) (Cell signaling technology, 9234).
Validation	All antibodies used in this work were purchased from companies, and validated by the manufacturers and by extensive use in published work.

Eukaryotic cell lines

Policy information about cell lines

Cell line source(s)	p53+/+HCT116 and p53-/- HCT116 cells were kindly gifted by Dr. Bert Vogelstein at John Hopkins University. other cell lines used in this study were purchased from ATCC, and National infrastructure of cell line resource (Bejing, China) respectively. In addition, all cell lines were cultured for no more than 2 months and their morphology was confirmed periodically to avoid crosscontamination or misuse of cell lines.

Authentication	The 293T cells were authenticated. To authenticate cell lines, we used short tandem repeat (STR) profiling method as exhaustively described by Dr. Asadi Jahanbakhsh and his/her colleagues (Khosravi Ayyoob et al, Tumor Biol. (2016) 37:3197–3204). No cells were listed in the commonly misidentified category.
Mycoplasma contamination	All cell lines were free of mycoplasma contamination (tested by MycoAlert kit, Lonza).
Commonly misidentified lines (See ICLAC register)	No commonly misidentified cell lines were used.

Animals and other organisms

Policy information about studies involving animals; ARRIVE guidelines recommended for reporting animal research

Laboratory animals	For xenograft experiment, 6- to 7-week-old male athymic Balb-c nu/nu male mice were used. Trp53+/+ and Trp53-/- mice were purchased from Beijing Biocytogen Co., Ltd. 6- to 8-week-old male Trp53+/+ and Trp53-/- mice (C57/BL6) were used as indicated in the figure legends. Experimental procedures were approved by the Animal Care and Use Committee of Tsinghua University.
Wild animals	This study did not involve these subjects.
Field-collected samples	This study did not involve these samples.
Ethics oversight	All animals were treated and used with approval of he Animal Care and Use Committee of Tsinghua University.

Note that full information on the approval of the study protocol must also be provided in the manuscript.

Flow Cytometry

Plots

Confirm that:

- [x] The axis labels state the marker and fluorochrome used (e.g. CD4-FITC).
- [x] The axis scales are clearly visible. Include numbers along axes only for bottom left plot of group (a 'group' is an analysis of identical markers).
- [x] All plots are contour plots with outliers or pseudocolor plots.
- [x] A numerical value for number of cells or percentage (with statistics) is provided.

Methodology

Sample preparation	For retroviral infection, EL4 cells were spin-infected with viral solution at 1,500 g in the presence of 4 μg/ml polybrene for 2 hours at 32 °C. Infected EL4 cells were expanded and GFP positive cells were sorted out by FACSArial II (BD Biosciences, USA) to obtain EL4-Luc-GFP cells.<br>For xenograft model established through i.v., viable EL4-Luc-GFP cells were washed twice with PBS and resuspended in PBS with the density of 5×106 cells/ml, which were subsequently injected into the lateral tail vein in a volume of 0.1 ml, and mice were then subjected to treatments as indicated in the methods. After xenograft, blood samples were collected from the tail vein. 20 μl volume of blood samples were added into PBS with 10% FBS and anticoagulant, after the procedure of red blood cell lysis, the residual white cells were resuspended in PBS with 10% FBS and subjected to FACS analysis.<br>Other stainings were performed on live cells according to manufacturer's protocol.
Instrument	FACSArial II (BD Biosciences, USA); LSR II cytometer (BD Biosciences, USA)
Software	Flowjo was used to collect the data
Cell population abundance	For GFP positive-sorted cells, the purity was determined by the percentage of GFP cells during sorting.
Gating strategy	For sorting GFP-postive cells, Positive GFP control: GFP expressing cells. Negative GFP control: non-infected cells. Gate was applied excluding the GFP-negative cells and including the GFP-positive cells only. For AnnexinV and /or PI staining, positive AnnexinV and/or PI was defined for cells incubated with AnnexinV-Alex680 and/or PI.<br>Like positive control, Negative AnnexinV control was defined for cell not incubated with AnnexinV-Alex680 and/or PI. A detailed description was provided in Supplementary Figure 11.

- [x] Tick this box to confirm that a figure exemplifying the gating strategy is provided in the Supplementary Information.
